# Decreased degree of adipocyte differentiation in visceral adipose tissue contributes to metabolic dysfunction-associated steatotic liver disease

**DOI:** 10.1038/s41467-026-73660-6

**Published:** 2026-06-03

**Authors:** Kyla Z. Gelev, Seung Hyuk T. Lee, Marcus Alvarez, Rosellina M. Mancina, Federica Tavaglione, Oveis Jamialahmadi, Umberto Vespasiani-Gentilucci, Asha Kar, Zitian Wang, Dorota Kaminska, Minna U. Kaikkonen, Ville Männistö, Sini Heinonen, Tuure Saarinen, Anne Juuti, Kirsi H. Pietiläinen, Jussi Pihlajamäki, Stefano Romeo, Päivi Pajukanta

**Affiliations:** 1 Department of Human Genetics, David Geffen School of Medicine at UCLA, Los Angeles, CA USA; 2 Department of Life Science, Health, and Health Professions, Link Campus University, Rome, Italy; 3 Centre for Reproduction, Metabolism and Molecular medicine (CeRM), Department of Medicine (H7), Karolinska Institute, Huddinge, Sweden; 4 Department of Molecular and Clinical Medicine, University of Gothenburg, Gothenburg, Sweden; 5 Research Unit of Clinical Medicine and Hepatology, Department of Medicine and Surgery, Università Campus Bio-Medico di Roma, Rome, Italy; 6 MASLD Research Center, Division of Gastroenterology and Hepatology, University of California at San Diego, La Jolla, California USA; 7 Operative Unit of Clinical Medicine and Hepatology, Fondazione Policlinico Universitario Campus Bio-Medico, Rome, Italy; 8 Bioinformatics Interdepartmental Program, UCLA, Los Angeles, CA USA; 9 Division of Cardiology, Department of Medicine, UCLA, Los Angeles, CA USA; 10 Institute of Public Health and Clinical Nutrition, University of Eastern Finland, Kuopio, Finland; 11 A. I. Virtanen Institute for Molecular Sciences, University of Eastern Finland, Kuopio, Finland; 12 Institute of Clinical Medicine, Internal Medicine, University of Eastern Finland, Kuopio, Finland; 13 Department of Internal Medicine, Kuopio University Hospital, Kuopio, Finland; 14 Department of Internal Medicine and Rehabilitation, Helsinki University Hospital, Helsinki, Finland; 15 Obesity Research Unit, Research Program for Clinical and Molecular Metabolism, Faculty of Medicine, University of Helsinki, Helsinki, Finland; 16 Department of Abdominal Surgery, Abdominal Center, Helsinki University Hospital and University of Helsinki, Helsinki, Finland; 17 Healthy Weight Hub, Abdominal Center, Helsinki University Hospital and University of Helsinki, Helsinki, Finland; 18 Department of Medicine, Endocrinology and Clinical Nutrition, Kuopio University Hospital, Kuopio, Finland; 19 Department of Endocrinology, Karolinska University Hospital, Huddinge, Sweden; 20 Department of Medical and Surgical Sciences, Magna Graecia University, Catanzaro, Italy; 21 Department of Cardiology, Sahlgrenska University Hospital, Gothenburg, Sweden; 22 Institute for Precision Health, David Geffen School of Medicine at UCLA, Los Angeles, CA USA

**Keywords:** Non-alcoholic fatty liver disease, Transcriptomics, Obesity

## Abstract

The mechanisms connecting the human fat depots, subcutaneous adipose tissue (SAT) and visceral adipose tissue (VAT), to metabolic dysfunction-associated steatotic liver disease (MASLD) remain elusive. We hypothesize that in individuals with obesity, a decreased degree of adipocyte differentiation may contribute to ectopic fat accumulation in the liver, seen in MASLD. Here we show, using single nucleus RNA-sequencing from adipose tissue biopsies, that the predicted degree of VAT adipocyte differentiation is decreased in individuals with MASLD, with an attenuated impact observed in SAT adipocytes. Next, we discover that regional variants of the VAT adipocyte differentiation gene set explain a substantial proportion (17%) of MASLD heritability. These genes largely overlap (>50%) with adipocyte genes differentially expressed by MASLD, regulated by variants in *cis*. Finally, we show that these genes are linked to smaller adipocyte size. Together, our findings reveal that a decreased predicted degree of VAT adipocyte differentiation contributes to MASLD.

## Abstract (web summary)

Metabolic dysfunction-associated steatotic liver disease (MASLD) is rising in prevalence globally. Here, the authors reveal that a decreased degree of adipocyte differentiation in visceral adipose tissue contributes to MASLD development.

## Introduction

In the last two decades, obesity has climbed in pervasiveness globally^1,2^. With it, metabolic disorders associated with abdominal obesity^3,4^, including metabolic dysfunction-associated steatotic liver disease (MASLD), have wrought unprecedented health complications, resulting in increased mortality rate^5–7^. Adipose tissue is involved in lipid metabolism and curtailing adverse ectopic fat accumulation^8,9^. Adipocytes undergo remodeling to accommodate the increase in lipids through hyperplasia, increasing in cell number, and hypertrophy, increasing in cell size^10^. However, previous mouse studies have suggested that in MASLD, this expansion fails, leading to the aggregation of lipids in the liver^11,12^. Therefore, investigating the major human fat depots, visceral adipose tissue (VAT) and subcutaneous adipose tissue (SAT), is critical to understand the development of MASLD.

MASLD currently affects 38% of adults worldwide^13^ and often goes undiagnosed^14^. Without intervention, MASLD may progress into its pro-inflammatory and pro-fibrotic stage, metabolic dysfunction-associated steatohepatitis (MASH) or to cirrhosis and even hepatocellular carcinoma^15,16^. Given that the prevalence of MASLD is expected to increase to over 55% by 2040^13^, it is imperative to deduce the mechanisms contributing to this redistribution of lipids from adipose tissue depots to the liver. While studies using bulk RNA-sequencing have been used to better understand this relationship, particularly the communication between the liver and VAT^17^, cellular heterogeneity in bulk tissue analysis has not enabled the analysis of adipocytes at a granular level or assess their role in MASLD development.

Recent single cell studies have revealed subtypes underlying the functional behaviors of adipocytes involved in lipid regulation and thermogenesis, highlighting the heterogeneity and plasticity of adipocytes in response to metabolic syndrome^18–20^. While adipose dysfunction has been proposed to lead to MASLD in individuals with obesity^9,21^, most studies have been limited to SAT bulk RNA-sequencing and microarray-based expression data^22,23^. Challenges in obtaining VAT biopsy samples due to the invasiveness of the procedure have caused it to be understudied. Despite this, VAT is suggested to hold the potential in determining a person’s susceptibility to metabolic syndrome^4,20^. In line with this, we hypothesize that a lower degree of adipocyte differentiation in VAT, more so than in SAT, contributes to the development of MASLD. Toward this end, we obtained VAT and SAT samples from individuals with obesity who underwent bariatric surgery, coupled with their histology-based liver assessment. We performed single nucleus RNA-sequencing (snRNA-seq) to investigate these depots at the single cell level. First, we compare the predicted degree of VAT adipocyte differentiation between the individuals with and without MASLD to uncover the role of VAT in the pathogenesis of MASLD. In parallel, we conducted similar analysis in SAT. We also examine how consequential sex is in remodeling both depots and how the expression of the MASLD genes predicting the differentiation degree relate to adipocyte size.

We discover that the predicted degree of VAT adipocyte differentiation is decreased in individuals with MASLD. Furthermore, regional DNA variants in these underlying genes explain a large amount of heritability in MASLD. We also highlight that these genes greatly overlap with the genes differentially expressed by MASLD in VAT adipocytes. Overall, we show that a decreased degree of predicted VAT adipocyte differentiation and its genetic predisposition contribute to the development of MASLD.

## Results

### Study design for predicting the degree of adipocyte differentiation in VAT and SAT and its links to MASLD

**Fig. 1:**
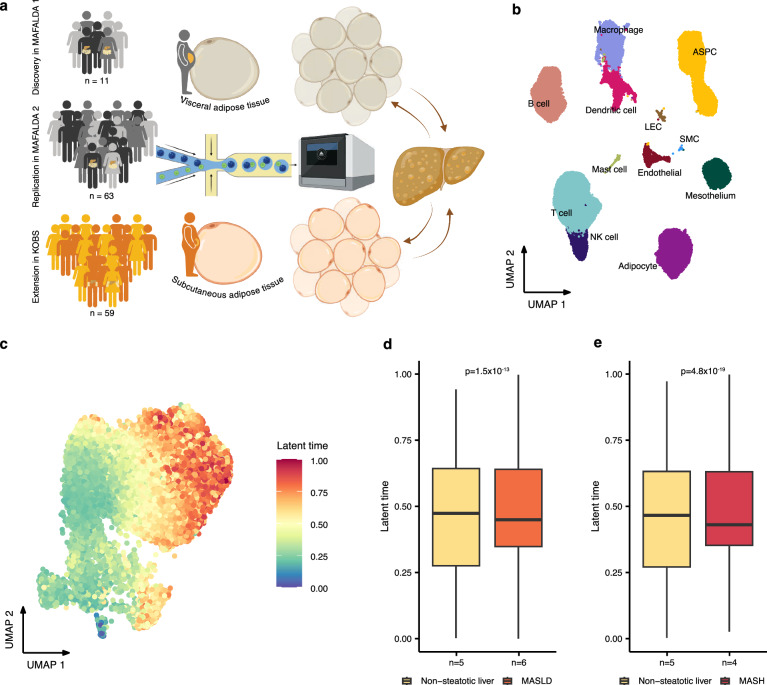
Decreased predicted degree of VAT adipocyte differentiation by MASLD. **a** Schematic overview of the study design. We used discovery (*n* = 11) and replication (*n *= 63) VAT single nucleus RNA-seq (snRNA-seq) data sets from the MAFALDA cohort. We also extended the latent time investigations across tissues to a subcutaneous adipose tissue (SAT) snRNA-seq data set (*n* = 59) from the KOBS cohort. **b** Uniform Manifold Approximation and Projection (UMAP) visualization of 85,441 nuclei from the VAT snRNA-seq data in MAFALDA 1. **c** UMAP visualization of adipocytes, colored by the latent time values, blue indicating an earlier latent time and red indicating a later latent time. **d** Boxplots show a lower latent time in the individuals with MASLD (either steatosis or MASH) (*n *= 6), when compared to the individuals with non-steatotic livers (*n* = 5) using the two-sided Wilcoxon rank sum test. **e** Boxplots show a lower latent time in the individuals with MASH (*n* = 4) when compared to the individuals with non-steatotic livers (*n* = 5) using the two-sided Wilcoxon rank sum test. Abbreviations: VAT indicates visceral adipose tissue; SMC (vascular) smooth muscle cell; ASPC adipose stem and progenitor cells; LEC lymphatic endothelial cells; NK cells natural killer cell; MASLD metabolic dysfunction-associated steatotic liver disease; and MASH, metabolic dysfunction-associated steatohepatitis. Panel **a** was created in BioRender. Gelev, K. (https://BioRender.com/rbovx2f). In. Panels **d**, **e** data are represented as a boxplot with whiskers indicating 1.5x the interquartile range (minimum and maximum), upper and lower bounds showing the 25^th^ and 75^th^ percentiles, and the middle lines depicting the median values. Outliers are indicated by singular dots. Source data are provided as a Source Data file.

We hypothesized that a lower degree of VAT adipocyte differentiation contributes to metabolic dysfunction-associated steatotic liver disease (MASLD) in the liver. Therefore, VAT dysfunction in MASLD and MASH, i.e., the inability of VAT adipocytes to become fully matured adipocytes, may ultimately contribute to the accumulation of ectopic fat in the liver, inducing MASLD. Using a latent time analysis of adipocytes, we set out to investigate this by first identifying the genes that predict the degree of adipocyte differentiation in VAT adipocytes from the individuals with MASLD versus those without MASLD. Following this, we assessed how much these VAT adipocyte latent time genes explain of MASLD heritability. For this study, we examined VAT single nucleus RNA-sequencing (snRNA-seq) data from a discovery cohort of 11 individuals in the MAFALDA cohort with liver histology-based assessment, referred to as MAFALDA 1, followed by validation in 63 independent individuals from the same cohort, referred to as MAFALDA 2. We then extended our investigations to subcutaneous adipose tissue (SAT) to compare our findings across the key human fat depots using SAT snRNA-seq data from the KOBS cohort (*n* = 59), with MASLD defined by liver histology-based assessment (Fig. 1a) (for clinical characteristics, see Supplementary Data 1). Finally, we tested how the genes predicting the VAT adipocyte differentiation degree relate to VAT adipocyte size in an independent RYSA cohort (*n* = 66 individuals with obesity).

### Decreased predicted degree of VAT and SAT adipocyte differentiation observed in individuals with MASLD

After performing quality control (QC) (see “Methods”), in MAFALDA 1, we clustered and annotated cell-types using the previous VAT single cell atlas^19^ as reference (Fig. 1b, and Supplementary Fig. 1) followed by subsetting adipocytes separately to assess whether adipocyte functional activity differed by MASLD. Using dynamical modeling from scVelo^24^ (see “Methods”), we obtained cell-level latent time values in adipocytes (Fig. 1c). We used latent time as a proxy for the predicted degree of adipocyte differentiation. In this study, a lower latent time implies that a cell is predicted to be earlier in its differentiation trajectory, or it is predicted to be less differentiated. A higher latent time indicates that the cell is further along in its predicted differentiation trajectory (see “Methods”). We observed that individuals with MASLD had a lower latent time in VAT adipocytes than individuals without MASLD (*p* = 1.5 × 10^−13^) (Fig. 1d, and Supplementary Data 2), even after adjustment for confounders and technical factors (see Methods). We observed similar differences in the individuals with and without metabolic dysfunction-associated steatohepatitis (MASH) (*p *= 4.8 × 10^−19^) (Fig. 1e, and Supplementary Data 3).

**Fig. 2:**
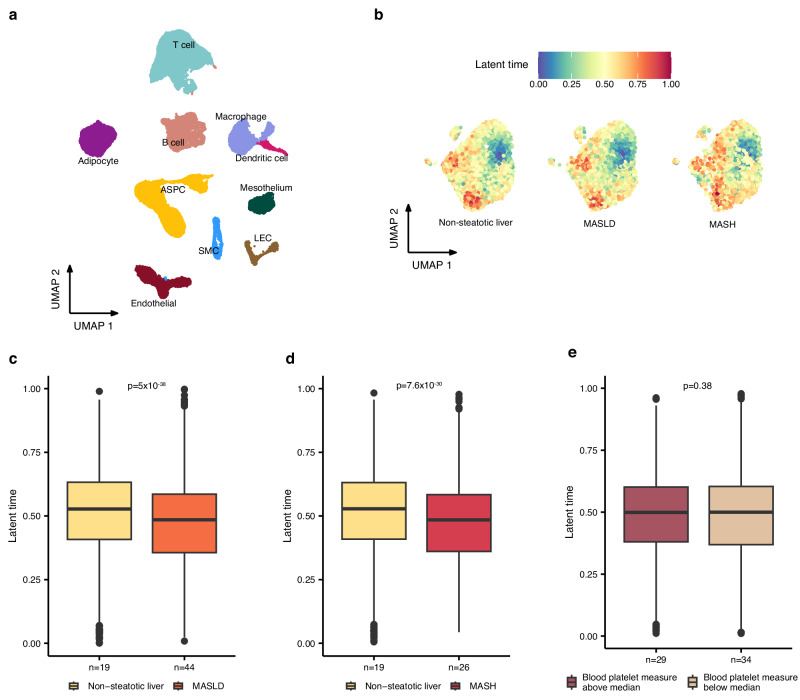
Replication of the delayed adipocyte latent time in MASLD and MASH. **a** Uniform Manifold Approximation and Projection (UMAP) visualization of 84,063 nuclei from the VAT snRNA-seq data in the MAFALDA 2 data set (*n* = 63). **b** UMAP visualization of adipocytes, colored by the latent time values, blue indicating an earlier latent time and red indicating a later latent time, and split by the liver histology-based groups. **c** Boxplots show a lower latent time in the individuals with MASLD (either steatosis or MASH) (*n* = 44) when compared to the individuals with non-steatotic livers (*n* = 19) using the two-sided Wilcoxon rank sum test. **d** Boxplots show a lower latent time in the individuals with MASH (*n* = 26) when compared to the individuals with non-steatotic livers (*n* = 19) using the two-sided Wilcoxon rank sum test. **e** Boxplots showing a non-significant difference of the latent time in adipocytes between the above and below median blood platelet measurements using the two-sided Wilcoxon rank sum test. Abbreviations: hAd indicates human adipocyte subtype; SMC, (vascular) smooth muscle cell; ASPC adipose stem and progenitor cells; LEC lymphatic endothelial cells; NK cells, natural killer cell; MASLD metabolic dysfunction-associated steatotic liver disease; and MASH, metabolic dysfunction-associated steatohepatitis. In the boxplots of panels **c–e**, the boundary points show the 25^th^ and 75^th^ percentile, the middle lines the median latent time values, and the whiskers 1.5x of the interquartile range (minimum and maximum). Outliers are represented by the single dots. Source data are provided as a Source Data file.

To validate these results, we utilized the MAFALDA 2 cohort. Similarly to MAFALDA 1, we clustered and annotated the cell-types (Fig. 2a, and Supplementary Fig. 2), subset the adipocytes, and then determined the latent time of each cell. As in MAFALDA 1, individuals with MASLD and MASH had fewer VAT adipocytes reaching their final terminal endpoints (Fig. 2b), both visually and quantitatively (*p* = 5 × 10^−38^ and *p* = 7.6 × 10^−30^, respectively) (Fig. 2c, Supplementary Data 4 and Fig. 2d, Supplementary Data 5). We also found that insulin levels were associated with MASLD and latent time, identifying that a lower predicted degree of adipocyte differentiation is associated with higher insulin levels (Supplementary Note 1.1, Supplementary Data 6). Upon performing the latent time analysis in SAT adipocytes, we observed a similar effect between the individuals with MASLD and non-steatotic livers, though at an attenuated degree (Supplementary Note 1.2, Supplementary Figs. 3–6, Supplementary Data 7, 8). However, sex exhibited a much larger influence on the predicted degree of adipocyte differentiation in SAT than VAT (Supplementary Notes 1.2–1.4, Supplementary Figs. 7–12, and Supplementary Data 9–21).

Furthermore, even when adjusting for rs738409^25^ in *PNPLA3*, a known MASLD variant, we observed similar results (Supplementary Note 1.5, Supplementary Figs. 13, 14, and Supplementary Data 22–24). Blood platelet count was used as a negative phenotypic control trait (*p* = 0.38) (Fig. 2e, and Supplementary Data 25) because we did not find it to be associated with MASLD, MASH, or fibrosis in MAFALDA 2 (*p* > 0.05) (Supplementary Data 26). Taken together, the latent time results observed in MAFALDA 1 replicated in MAFALDA 2 (*p* < 0.05 in the same direction of effect).

To provide more insight into the latent time results, we re-performed the analysis with scVelo^24^ using the overlapping genes between a previously published VAT adipogenesis experiment (Supplementary Data 27) and the predicted degree of adipocyte differentiation (i.e., adipocyte latent time) gene set (*n* = 666 genes). Using these 666 genes, referred to as the subset of the predicted degree of adipocyte differentiation gene set, we found that our results were in line with the observation that the individuals with MASLD and MASH have a lower adipocyte latent time when compared to the control group (Supplementary Note 1.6, Supplementary Fig. 15, Supplementary Data 28-30).

In comparison, when measuring the latent time of hepatocytes from a MASLD case/control cohort with liver histology assessments^26^, we found that the individuals with MASLD and MASH had a significantly higher latent time compared to the controls with non-steatotic liver (*p* = 1.4 × 10^−82^ for MASLD and *p* = 1.6 × 10^−7^ for MASH, respectively) (Supplementary Fig. 16, Supplementary Data 31, 32). Investigating this further, we found that the hepatocytes coinciding with zone 3 exhibited a higher latent time (Supplementary Fig. 17). Hepatocytes are known to exhibit distinct functions based upon their zonation, with zone 3 being involved in lipogenesis, while other zones such as zone 1 are involved in beta-oxidation^27^. Thus, in hepatocytes, individuals with MASLD and MASH have a higher latent time, and in VAT adipocytes individuals with MASLD and MASH have a lower latent time.

To better characterize the hepatocytes with increased latent time, we overlapped the genes used to estimate hepatocyte latent time, i.e., the predicted degree of hepatocyte differentiation gene set (*n* = 2000), with the genes (*n* = 4,122 genes) that were DE by MASLD in hepatocytes (Supplementary Data 33). Among those, 1040 genes were DE by MASLD and contributed to the predicted degree of hepatocyte differentiation rate. We found that the genes that were upregulated by MASLD and involved in predicting the differentiation degree for hepatocytes (i.e., hepatocyte latent time gene set) (*n* = 417 genes) were enriched in cholesterol and sterol esterification pathways (Supplementary Fig. 18, and Supplementary Data 34). This also matches with the characterization of zone 3 hepatocytes that we predicted to have an increased latent time. Both cholesterol esterification and lipogenesis are linked to tissue remodeling in the liver as a response to the influx of lipids in individuals with MASLD. This is in contrast with the genes downregulated by MASLD from the hepatocyte latent time gene set (*n* = 623 genes) that were enriched for other liver-specific processes such as regulation of copper and zinc (Supplementary Fig. 18, Supplementary Data 35). Based upon this, the hepatocytes with a higher latent time corresponding to individuals with MASLD seem to be involved in hepatocyte remodeling to accommodate the increase in lipids.

### Key triglyceride-regulating adipocyte subtype is diminished in individuals with MASH

**Fig. 3:**
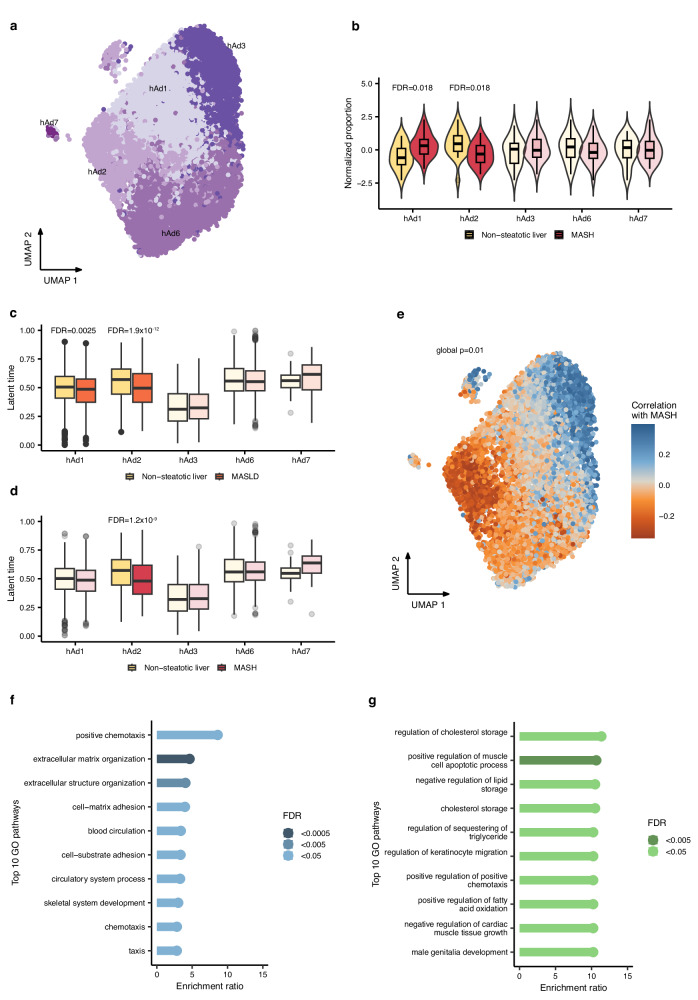
Investigation of the underlying adipocyte subtypes linked to MASLD and MASH in VAT. **a** Uniform Manifold Approximation and Projection (UMAP) visualization of adipocytes, colored by subtypes. **b** Violin plots showing the adjusted normalized proportions of adipocyte subtypes between the individuals with non-steatotic (*n* = 19) livers and MASH (*n* = 26). Adipocyte subtypes with differences (FDR < 0.05) in the proportions between the pairwise combinations of the liver histology-based groups are shown by the two-sided Wilcoxon rank sum test. **c** Boxplots show a lower latent time (FDR < 0.05) in the individuals with MASLD livers (either steatosis or MASH) (n = 44) vs non-steatotic livers (*n* = 19) in hAd1 and hAd2, determined by pairwise comparisons using the two-sided Wilcoxon rank sum test. **d** Boxplots show a lower latent time (FDR < 0.05) in the individuals with MASH livers (*n* = 26) when compared to the individuals with non-steatotic livers (*n* = 19) in hAd2 by pairwise comparisons using the two-sided Wilcoxon rank sum test. **e** UMAP visualization of adipocytes, colored by correlation (neighborhood coefficient) with MASH determined by empirical permutation test. Subtype hAd1 is positively associated with MASH (blue) and hAd2 is negatively associated with MASH (red). **f** Horizontal lollipop plots show top 10 (FDR < 0.05) GO functional pathway enrichments for adipocyte subtype hAd1. **g** Horizontal lollipop plots show top 10 (FDR < 0.05) GO functional pathway enrichments for adipocyte subtype hAd2. Abbreviations: hAd indicates human adipocyte subtype; MASLD, metabolic dysfunction-associated steatotic liver disease; MASH, metabolic dysfunction-associated steatohepatitis; and GO, Gene Ontology. **b** shows violin plots representing the upper and lower bounds (25^th^ and 75^th^ percentiles), and the middle lines indicating the median subtype proportions, for which the covariates have been regressed out and inverse normalized. The whiskers indicate the distance that spans 1.5x the interquartile range (minimum and maximum) with outliers being shown as dots. In boxplots of panels (**c**, **d**) boundary points show the 25^th^ and 75^th^ percentile, middle lines the median latent time values, and whiskers 1.5x of the interquartile range (minimum and maximum). Outliers are represented by single dots. Shading in these panels showcase the significant adipocyte subtypes. Source data are provided as a Source Data file.

Adipocytes are a highly heterogeneous cell-type and exhibit distinct metabolic features, such as storing triglycerides and oxidating fatty acids^28,29^. Therefore, to understand the heterogeneity of adipocyte subtypes in MASLD, we performed subtyping using the previous VAT single cell atlas^19^ as a reference. This resulted in five distinct VAT subtypes (Fig. 3a). In MAFALDA 1, we verified the expression of the VAT adipocyte subtypes by calculating the module scores of these subtype marker genes from MAFALDA 2 in MAFALDA 1. Supplementary Fig. 19 demonstrates that all VAT adipocyte subtypes are also present in MAFALDA 1.

Next, we compared the adipocyte subtype proportions across the identified subtypes between the individuals with MASLD and MASH and the control group in MAFALDA 2. Though we did not find differences in subtype proportions in individuals with MASLD (Supplementary Fig. 20), we did observe a different proportion of hAd1 and hAd2 in those with MASH (Fig. 3b). Individuals with MASH had a higher proportion of hAd1 (FDR = 0.018) and a lower proportion of hAd2 (FDR = 0.018). These data suggest that the reduction in the VAT adipocyte subtype hAd2 is associated with MASH. When investigating whether the latent time differences are also present in VAT adipocyte subtypes, hAd1 exhibited a lower latent time (FDR = 0.0025) in the individuals with MASLD, while hAd2 had a lower latent time in both MASLD and MASH (FDR = 1.9 × 10^−12^, FDR = 1.2 × 10^−9^) (Fig. 3c, Supplementary Data 36 and Fig. 3d, Supplementary Data 37). No significant differences were observed in the other adipocyte subtypes after an FDR correction. These data suggest that the lower latent time of the individuals with MASLD and MASH in hAd2 reflects a VAT subtype linked to MASLD.

To further understand the relationship between the VAT adipocyte subtypes and MASH, we performed an association analysis using covarying neighborhood analysis^30^ (CNA). This analysis searches for abundant cellular neighborhoods correlated with MASH. Based upon the permuted *p* of 0.01, there is an association between subcellular neighborhoods and MASH among the VAT adipocytes. Thus, the significant CNA result (global permuted *p* = 0.01) (Fig. 3e) further showcases the correlations between subcellular neighborhoods and MASH among the VAT adipocytes.

Next, we investigated the function of the VAT adipocyte subtypes using their marker genes, i.e., the genes highly expressed in a subtype when compared to other subtypes (see “Methods”). We found 252 marker genes in adipocyte subtype hAd1, 246 in hAd2, 506 in hAd3, 141 in hAd6, and 195 in hAd7, respectively (Supplementary Data 38). In addition, we separately investigated the adipocyte subtype marker genes that are part of the adipocyte latent time gene set (Supplementary Data 39). Of these, 144 were marker genes for adipocyte subtype hAd1, 143 in hAd2, 150 in hAd3, 46 in hAd6, and 40 in hAd7, respectively. Investigating the key marker genes defining these subtypes (Supplementary Data 38), we found that the hAd1 marker genes were (FDR < 0.05) enriched in early progenitor pathways^31–33^ including extracellular matrix organization (ECM) and positive chemotaxis (Fig. 3f, and Supplementary Data 40), while hAd2 showed (FDR < 0.05) enrichment for triglyceride and cholesterol regulatory pathways (Fig. 3g, and Supplementary Data 41). The enrichment of the hAd1 pathways suggests a state of underdevelopment, exhibiting more preadipocyte-specific functions. The ECM is crucial for structure, preadipocyte differentiation, and intercellular communication^34–36^. Furthermore, chemotaxis, refers to the movement of cells in response to a chemical stimulus. In the context of adipogenesis, this may be a result of growth factors such as *FGF2*, *VEGFC*, and *ANGPT1*, which are marker genes for subtype hAd1. These genes act as regulators of adipogenesis^37^ and modulate angiogenesis^38–40^. Thus, coupled with the reduction in hAd2, an adipocyte subtype with marker genes involved in the homeostasis of neutral lipids, these adipocytes in individuals with MASLD appear to remain as underdeveloped adipocytes. They express more fibroblastic-like markers and are unable to reach their full adipogenic potential. Furthermore, hAd2 seems poorly maintained specifically in individuals with MASH.

### Lower predicted degree of adipocyte differentiation correlates with smaller adipocytes

To investigate whether reduced adipocyte latent time correlates with adipocyte size, we used the adipocyte size measurements and VAT bulk RNA-seq data in RYSA and performed a gene set enrichment analysis. We first identified genes, the VAT expression of which is associated with the adipocyte diameter in RYSA, adjusting for VAT cell-types proportions, age, sex, BMI, and technical factors (see “Methods”). We used the following VAT bulk expressed genes among the predicted degree of VAT adipocyte differentiation gene set for the pathway parameters: the 263 genes that are DE by MASLD or MASH; the 145 genes that are upregulated by MASLD or MASH; and the 118 genes that are downregulated by MASLD or MASH (Supplementary Data 42). Following this, we found that there is a negative normalized enrichment score (NES) (NES = −1.77, *p* value = 9.17 × 10^−7^) between the gene list that was ranked based on the correlation of the VAT expression with adipocyte diameter in RYSA and the 263 genes DE by MASLD or MASH in VAT adipocytes in MAFALDA. Furthermore, we found that this negative NES is mostly reflecting the 145 DE genes upregulated by MASLD or MASH (NES = −2.19, *p* value = 3.27 × 10^−10^). Using the genes that contributed most to the enrichment of this upregulated gene set (*n* = 48 genes), we built the first principal component (PC1) of their VAT bulk expression and identified a negative trend (rho = −0.242, *p* value = 0.057) between PC1 of the VAT bulk expression and the adipocyte diameter in RYSA (Supplementary Note 1.7, Supplementary Data 43). This shows that there is a negative association between the expression of the VAT MASLD DE genes predicting the differentiation degree and adipocyte diameter in the RYSA cohort.

### Variants in *cis* regions of the genes predicting the differentiation degree of VAT adipocytes explain a large amount of heritability of MASLD traits

To first assess genetic associations of regional variants in the VAT adipocyte latent time genes with MASLD, we employed MAGMA^41^ to perform an enrichment analysis of MASLD GWAS signals among these genes. The following gene sets were tested: the predicted degree of adipocyte differentiation gene set (i.e., the adipocyte latent time gene set), the ranked VAT genes (i.e., the genes with differing dynamic time by MASLD) from scVelo^24^, and the two sets of VAT subtype marker genes in the identified adipocyte subtypes, hAd1 and hAd2 (Supplementary Data 44). We found that all except the hAd1 gene set were enriched (*p* = 9.7×10^−4^, 2.5×10^−4^, 2.6×10^−4^) for ALT GWAS signals (see “Methods”) when adjusting for alcohol consumption (ALT 1), while all four gene sets were enriched (*p* = 2.3 × 10^−4^, 8.0 × 10^−6^, 5.1 × 10^−3^, 6.7 × 10^−4^) when omitting individuals with heavy alcohol intake (ALT 2) (see “Methods”) (Table 1). The latent time gene set and the overlapping genes between the ranked VAT gene sets from both MAFALDA 1 and MAFALDA 2 were also found to be enriched (*p* = 0.0083, *p* = 0.034) for proton density fat fraction (PDFF) GWAS variants (see “Methods”) (Table 1).

**Table 1:** The VAT adipocyte subtype and latent time gene sets show significant (*p *< 0.05) enrichments for GWAS signals in MASLD traits using the one-sided competitive gene-set analysis in MAGMA^41^

Outcome^#^	Gene set*	Number of GWAS genes	Standardized beta	*p*
ALT 1	Latent time gene set	1493	0.029	9.7 × 10^−4^
ALT 1	Ranked gene set	50	0.033	2.5 × 10^−4^
ALT 1	hAd2	211	0.033	2.6 × 10^−4^
ALT 2	Latent time gene set	1493	0.031	2.3 × 10^−4^
ALT 2	Ranked gene set	50	0.038	8.0 × 10^−6^
ALT 2	hAd1	203	0.023	5.1 × 10^−3^
ALT 2	hAd2	211	0.028	6.7 ×;10^−4^
PDFF	Latent time gene set	1480	0.017	0.0083
PDFF	Overlap of ranked gene set of MAFALDA 1 and MAFALDA 2	16	0.013	0.034

^#^ALT 1 refers to ALT adjusted for alcohol consumption while ALT 2 does not include individuals with heavy alcohol consumption. PDFF indicates proton density fat fraction.

^*^Latent time gene set indicates the genes in the predicted degree of adipocyte differentiation gene set; ranked gene set the genes with a differing transcriptional activity by MASLD (see “Methods”) and hAd1 and hAd2 gene sets the marker genes of each subtype, respectively. GWAS indicates genome-wide association study; *MASLD* metabolic dysfunction-associated steatotic liver disease; *ALT* alanine transaminase; and *hAd* human VAT adipocyte subtype.

**Fig. 4:**
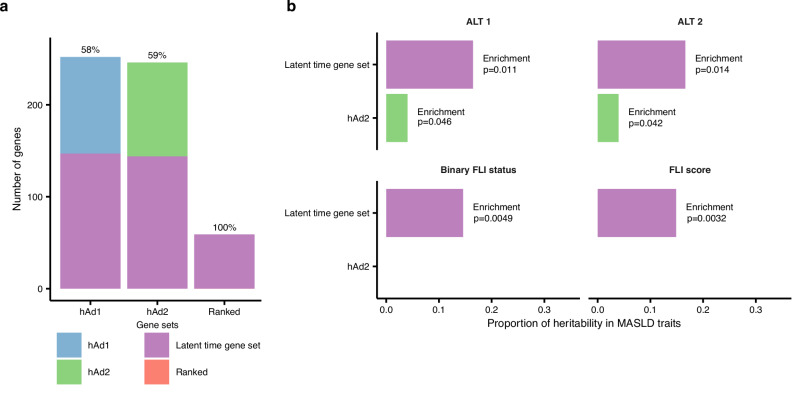
VAT adipocyte latent time and subtype hAd2 genes explain a large proportion of heritability in MASLD traits in the UK Biobank. **a** Barplot showcasing the large overlap of the genes from the VAT latent time gene set (i.e., the predicted degree of adipocyte differentiation gene set) across all of the other gene sets. **b** Horizontal barplots displaying the proportion of heritability explained by the variants in *cis* regions of the genes from the gene sets of interest with significant heritability enrichments (enrichment *p* < 0.05). Partitioned heritability was performed using LDSC^42^ for each MASLD trait in the UK Biobank, an independent cohort from the MAFALDA cohorts. Specifically, both the adipocyte latent time and hAd2 gene sets show enrichment for heritability of ALT (*p* = 0.011 and *p* = 0.014 for the latent time gene set; *p* = 0.046 and *p* = 0.042 for hAd2) while the latent time gene set also shows enrichment for heritability of a MASLD proxy, the fatty liver index (FLI) (*p* = 0.0049 and *p* = 0.0032). ALT 1 refers to ALT adjusted for alcohol consumption while ALT 2 does not include individuals with heavy alcohol consumption. Binary FLI status is assessed based upon FLI ≥ 60 for cases and FLI ≤ 30 for controls^98^. Abbreviations: hAd indicates human adipocyte subtype; *MASLD* metabolic dysfunction-associated steatotic liver disease; *MASH* metabolic dysfunction-associated steatohepatitis; *LDSC* LD score regression; *ALT* alanine transaminase; and *FLI* fatty liver index. Source data are provided as a Source Data file.

Next, we investigated whether these four gene sets harbor variants significantly contributing to the heritability in MASLD traits using LDSC^42^ in the UK Biobank (UKB) (see Methods). We first observed that the genes predicting the differentiation degree also exhibit a large (>50%) overlap across all the other gene sets (Fig. 4a). Then, we found that variants falling within the *cis* regions of this latent time gene set and hAd2 subtype marker gene set contributed a large amount of heritability (16.5% and 4.0% for ALT 1, alcohol adjusted, and 16.6% and 4.0% for ALT 2, no heavy alcohol consumption) in ALT with consistent enrichments (*p* = 0.011 and *p* = 0.046 for ALT 1, alcohol adjusted, and *p* = 0.014 and *p* = 0.042 for ALT 2, no heavy alcohol consumption). The predicted degree of adipocyte differentiation gene set also contributed to a large amount of heritability in a MASLD (or steatotic liver disease) surrogate, fatty liver index (FLI), both as a binary (14.6%) and continuous (14.9%) trait, with significant heritability enrichments (*p* = 0.0049 and *p* = 0.0032), respectively (Fig. 4b, Supplementary Data 45). Furthermore, we found that the subset of this gene set (666 genes), which overlapped with the bulk VAT adipogenesis DE genes (see “Methods”), still explained a significant proportion of heritability in ALT and FLI (Supplementary Fig. 21, Supplementary Data 46). Taken together, we show that genetic variants in the genes predicting the VAT adipocyte differentiation degree explain a large amount of heritability in MASLD traits, most likely through changing the adipocyte expression of these genes.

### Identifying adipocyte genes differentially expressed by MASLD, MASH, and sex and their genetic *cis* regulatory variants

Considering the context-specific adipocyte latent time results we discovered in VAT adipocytes and the GWAS and heritability enrichments for the MASLD traits, we next searched for VAT adipocyte genes differing by MASLD, MASH, or sex. Following this, we then conducted VAT adipocyte *cis*-expression quantitative trait locus (*cis*-eQTL) analysis of these DE genes to discover genetic variants regulating their adipocyte expression. Finally, we investigated which VAT adipocyte MASLD and MASH DE genes, regulated by the *cis*-eQTL variants, overlap with the predicted degree of adipocyte differentiation gene set that explains a large proportion of MASLD heritability. Here, we used the MAFALDA 2 for the DE and *cis*-eQTL analyses because the DE and *cis*-eQTL analyses cannot be performed in the MAFALDA 1 due to its sample size.

**Fig. 5:**
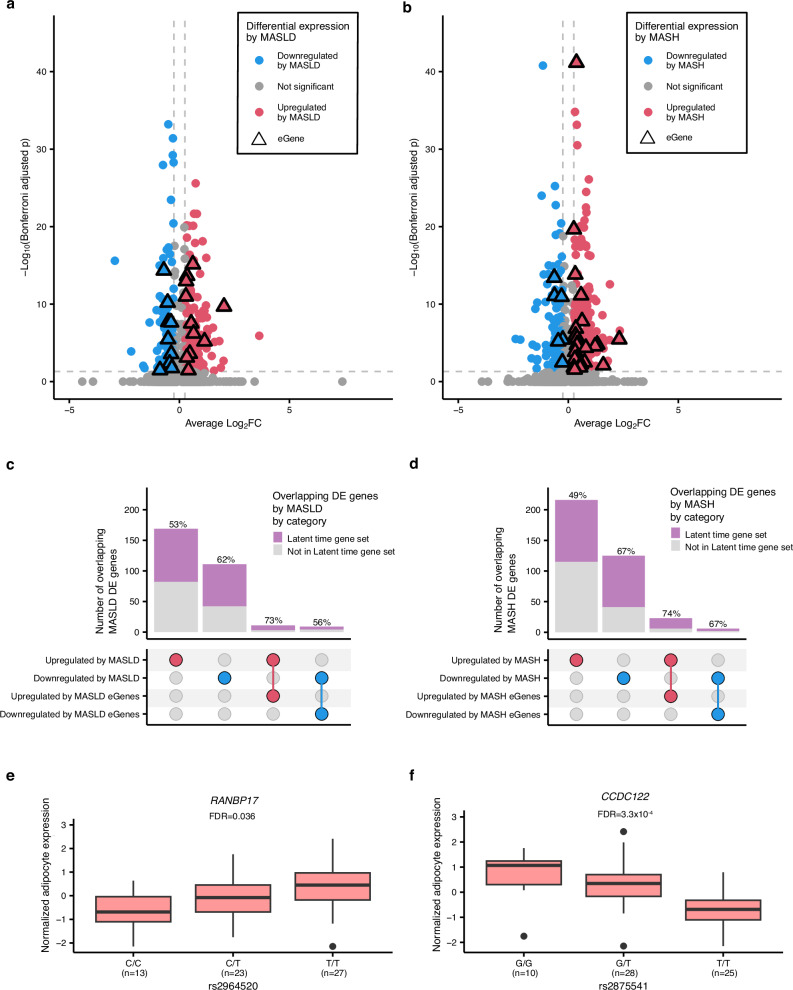
Greater than 50% of the VAT adipocyte genes DE by MASLD and MASH and regulated in *cis* overlap with the VAT latent time gene set. **a** Volcano plot showcasing differentially expressed (DE) genes by MASLD. DE genes by MASLD and adipocyte eGenes are indicated by triangles. **b** Volcano plot showcasing DE genes by MASH. DE genes by MASH and adipocyte eGenes are indicated by the triangles. **c** Upset plot of MASLD DE genes, featuring the intersection of the genes DE by MASLD of the following four categories: upregulated genes by MASLD, downregulated genes by MASLD, upregulated genes by MASLD that are also adipocyte eGenes, and downregulated genes by MASLD that are also adipocyte eGenes. **d** Upset plot of MASH DE genes, featuring the intersection of the genes DE by MASH of the following four categories: upregulated genes by MASH, downregulated genes by MASH, upregulated genes by MASH that are also adipocyte eGenes, and downregulated genes by MASH that are also adipocyte eGenes. **e** Boxplot shows the adipocyte *cis*-eQTL effect of SNP rs2964520 on normalized adipocyte expression of *RANBP17*. **f** Boxplot shows the adipocyte *cis*-eQTL effect of SNP rs2875541 on normalized adipocyte expression of *CCDC122*. Abbreviations: MASLD indicates metabolic dysfunction-associated steatotic liver disease; MASH metabolic dysfunction-associated steatohepatitis; and eGene, *cis*-eQTL target gene. DE analyses in (**a**, **b**) were done using the hurdle model approach in FindMarkers (see “Methods”) and significance determined by Bonferroni adjusted *p* value < 0.05 and absolute log_2_FC > 0.25. Red dots indicate significantly upregulated genes by respective condition while blue dots indicate significantly downregulated genes by respective condition. In panels **e** and **f**, colors represent presence or absence of the genes in the adipocyte latent time gene set across respective categories. The boxplots in these panels **e** and **f** represent the normalized adipocyte expression, with the upper and lower bounds as the 25^th^ and 75^th^ percentiles, the whiskers showing 1.5x the interquartile range of the normalized adipocyte expression, and the middle line indicating the median normalized adipocyte expression. Outliers are indicated by the single dots. Source data are provided as a Source Data file.

We performed the DE analyses using MAST^43^ by MASLD, MASH, and sex in VAT adipocytes, adjusting for biological and technical covariates (see “Methods”) (Supplementary Data 47). We found 300 VAT adipocyte DE genes by MASLD and 370 DE genes by MASH using Bonferroni adjusted *p *< 0.05. Of the 300 VAT adipocyte genes DE by MASLD, 20 genes were identified as adipocyte *cis*-eQTL target genes (i.e., adipocyte eGenes) using Matrix eQTL^44^, and of the 370 genes DE by MASH, 29 genes were adipocyte eGenes (Fig. 5a, b, and Supplementary Data 48). In addition, we observed 191 VAT adipocyte genes DE by sex that overlapped with 18 adipocyte eGenes (Supplementary Fig. 22, and Supplementary Data 48). Notably, these *cis*-regulated MASLD, MASH, and sex VAT DE eGenes include known adipogenesis genes, such as *SNTB2*^45^, *FOXO1*^46^, and *PIK3R1*^47^.

Next, we discovered that a large amount of the adipocyte DE genes by MASLD (56.3%, *p* = 8.6 × 10^−52^) and MASH (55.6%, *p* = 4.7 × 10^−62^) overlap with the adipocyte latent time gene set while only 33.2% (*p* = 1.7 × 10^−46^) of the adipocyte DE genes by sex overlap with this predicted degree of adipocyte differentiation gene set (Supplementary Fig. 22). Furthermore, these latent time genes also comprise the largest groups among the adipocyte MASLD and MASH DE genes that are eGenes (73% upregulated eGenes and 56% downregulated eGenes by MASLD; 74% upregulated eGenes and 67% downregulated eGenes by MASH) (Fig. 5c, d, and Supplementary Fig. 23), emphasizing the contributions of the genes predicting VAT adipocyte differentiation degree and their genetic regulation to MASLD.

Among all expressed genes in hepatocytes (27,200 genes), 143 genes overlap with the 169 adipocyte DE genes by MASLD that are also part of the gene set of the degree of adipocyte differentiation. Among these 143 overlapping genes (143/169; 84.6%), there are 46 genes that are also DE in hepatocytes (46/169; 27.2%). However, only 25 of these 46 DE genes match directionally (25/169; 14.8%) (i.e., the gene is upregulated both in adipocytes and hepatocytes or downregulated in both cell-types) (Supplementary Data 33). These relatively small overlaps of the DE genes in the same direction in both adipocytes and hepatocytes support and are in line with the different direction of the latent time by MASLD observed in adipocytes versus hepatocytes.

From the VAT adipocyte latent time gene set, we exemplify here *RANBP17*, upregulated by both MASLD and MASH, and identified as an adipocyte eGene (FDR = 0.036) (Fig. 5e). It is *cis* regulated by the variant, rs2964520, a BMI GWAS SNP. *RANBP17* encodes a nuclear transport receptor importin, and it has a very strong HuGE Score^48^ of 45 for multiple MASLD-related traits (AST-ALT ratio, high density lipoprotein (HDL) cholesterol, BMI, serum ApoA, plasma C-reactive protein, weight, and childhood obesity), thus providing additional independent evidence toward its genetic impact on these traits. We also exemplify *CCDC122*, a DE gene by sex, from this predicted degree of adipocyte differentiation gene set, the adipocyte expression of which is regulated by a *cis*-eQTL SNP, rs2875541 (FDR = 3.3 × 10^−4^) (Fig. 5f).

Taken together, we identified multiple important DE genes by MASLD, MASH, and sex and regulated locally in *cis* that largely overlap with the adipocyte latent time gene set. These DE and *cis*-eQTL results are in line with our significant MASLD GWAS and heritability enrichment results, showing that genetic variants in the genes used to predict the degree of adipocyte differentiation contribute to a large proportion of heritability in MASLD traits. Together, this convergingly indicates that a lower degree of VAT adipocyte differentiation is associated with MASLD in the liver.

## Discussion

Our study utilizes large cohorts of snRNA-seq data to characterize both VAT and SAT adipocytes and determine how much adipocytes in these human main fat depots contribute to MASLD. Noteworthy, we found that the predicted degree of differentiation in both VAT and SAT adipocytes is lower in individuals with MASLD compared to those without MASLD. Particularly, their latent time is lower in one key functionally enriched VAT adipocyte subtype, hAd2, with an important role in storing lipids and triglycerides. The relevance of the predicted degree of VAT adipocyte differentiation as a major contributor to the MASLD development was further substantiated by our finding that DNA variants around these adipocyte latent time genes explain almost 20% of heritability in MASLD traits in the UKB, independently from the snRNA-seq cohorts. In addition, these genes comprise >50% of the VAT adipocyte genes DE by MASLD. We also found that the genes predicting the degree of adipocyte differentiation (i.e., adipocyte latent time gene set) upregulated by MASLD and MASH are linked to smaller adipocytes in an independent VAT cohort. Taken together, we reveal that a decreased degree of VAT adipocyte differentiation contributes to MASLD in individuals with obesity.

We evaluated adipocyte remodeling in two separate subgroups of the VAT cohort, MAFALDA 1 and MAFALDA 2. Consistently in both data sets, we found a signature of a decreased VAT adipocyte latent time in MASLD and MASH in the VAT adipocyte subtype, hAd2, that seems to be responsible for lipid storage. In line with this, a previous study that used targeted mRNA gene expression analysis of inflammatory and fibrotic genes in human VAT biopsy samples observed that worse VAT impairment was associated with liver damage^49^.

Within liver, hepatocytes from individuals with MASLD and MASH had a higher latent time and these hepatocytes with the higher latent time corresponded to zone 3 hepatocytes involved in lipogenesis. The latent time analysis in VAT adipocytes in turn demonstrated that the individuals with MASLD and MASH have a lower adipocyte latent time than the controls with non-steatotic livers. This suggests that the VAT adipocytes from individuals with MASLD and MASH may be dysfunctional in their capacity to properly store fat, therefore promoting the ectopic fat accumulation, i.e., increased lipogenesis in the liver. Thus, the VAT latent time results of the individuals with MASLD and MASH may reflect the inadequate lipogenesis of their VAT adipocytes.

We found that VAT adipocyte subtype proportions change with progression of MASLD into MASH. Utilizing the previously published single cell adipose atlas^19^ we found that certain previously identified adipocyte subtypes were more common in the specific fat depots, including hAd4 in SAT and hAd6 in VAT, while both fat depots included subtypes hAd1 and hAd2. Subtype hAd1, more commonly identified in SAT^19^, was also detected in VAT and observed to be enriched for extracellular matrix (ECM) organization pathway genes, suggesting the presence of underdeveloped adipocytes that express more fibroblastic-like markers. The pathways, such as chemotaxis and ECM, that we found among the marker genes of hAd1, have been reported as indicators of adipocyte dysfunction^50,51^. Increased ECM remodeling has also been observed as an indicator of unhealthy expansion of adipocytes, causing hypoxia and fibrosis^51^. Therefore, the presence of these pathways among the marker genes of hAd1 suggests increased fibrogenesis in VAT. This underscores a pro-inflammatory and hypoxic microenvironment^52^ that contributes to MASLD. VAT adipocytes in individuals with MASLD may be reduced in their capacity to become fully differentiated adipocytes due to this microenvironment.

Subtype hAd2 has been identified as a basal adipocyte subtype in VAT^19,20^, implying its pertinence to VAT foundation itself. The previous studies also suggest that subtype proportions change with respect to BMI and metabolically unhealthy conditions, e.g., insulin resistance^19,20^, which we observe to be the case in individuals with MASLD. Notably, we found the VAT adipocyte subtype hAd2 to be largely depleted in individuals with MASH, hinting at an intrinsic impairment in the ability for VAT to store lipids.

The discovered increased proportion of hAd1 and the decreased proportion of hAd2 in the individuals with MASH indicate that VAT adipocytes are likely less differentiated, hampering their ability to store fatty acids and exacerbating to the accumulation of triglycerides in the liver. A previous human study investigated the preadipocyte-to-adipocyte transition and highlighted non-classical adipocytes as being an intermediate step to fully differentiated classical adipocytes^53^. Based upon their findings, we postulate that we are observing a similar effect; however, we now show that individuals with MASH appear to remain in this intermediate subcellular stage (i.e., adipocyte subtype hAd1).

In addition, we found that the VAT adipocyte expressed genes DE by MASLD and MASH, exhibit over a 50% overlap with the adipocyte latent time gene set. Regional DNA variants in this gene set, that also overlapped with the majority of subtype marker genes in VAT adipocyte subtypes hAd1 and hAd2, contributed a large proportion of heritability for MASLD traits in the UKB, particularly ALT and FLI. Thus, we observed a genetic effect of the regional variants in these latent time genes on the MASLD outcome. Moreover, we also found that >50% of the genes containing *cis* variants regulating their adipocyte expression are part of the latent time gene set, thus further associating the predicted degree of VAT adipocyte differentiation with MASLD.

Although we investigate both VAT and SAT from individuals with MASLD, we were limited in our ability to assess how SAT is involved in the pathogenesis from MASLD to MASH due to the number of MASH cases in our SAT snRNA-seq cohort. We also recognize that our results do not prove causality and demonstrating the adipocyte dysfunction as a putative causal mechanism leading to MASLD requires further experimental analyses, such as animal studies. These findings also prompt for a study into whether new adipocytes can be recruited from ASPCs to combat MASLD. An additional limitation is that we were unable to obtain matched VAT and SAT biopsies from the same individuals, which would have diminished the potential for confounders, such as varying degrees of MASLD and population-based differences between Italians and Finns. To address this, we adjusted analyses throughout for both technical and biological covariates. Due to the use of two European snRNA-seq cohorts and Europeans from the independent UKB, our results can likely be generalized to Europeans; however, we recognize the need for future studies in additional populations to identify population-based differences.

In conclusion, we discovered that a decreased predicted degree of adipocyte differentiation in VAT adipocytes and a distinct VAT adipocyte subtype contribute to MASLD. We also identified the corresponding key gene sets, explaining a significant amount of MASLD trait heritability. To emphasize these findings, we also identified *cis* variants governing these genes that were enriched for DE genes by MASLD and explained a large proportion of the heritability of the MASLD traits in an independent analysis of UKB. Altogether, this study demonstrates a previously unknown key link between VAT adipocyte dysfunction and MASLD susceptibility.

## Methods

### Ethics

The Molecular Architecture of FAtty Liver Disease in individuals with obesity undergoing bAriatric surgery (MAFALDA) study was approved by the Local Research Ethics Committee at the Campus Bio-Medico University Hospital of Rome (Italy) (approval no. 16/20) and by the Swedish Ethics Review Authority (Dnr 2025-08073-01 and Dnr 2025-06169-01). The Kuopio OBesity Surgery Study (KOBS) study was approved by the Ethics Committee of the Northern Savo Hospital District (54/2005, 104/2008, and 27/2010). The Roux-en-Y versus one-anastomosis gastric bypass (RYSA) cohort was approved by the Helsinki University Hospital Ethics Committee (HUS/1706/2016). The UK Biobank study was approved by the North West Multi-center Research Ethics Committee (21/NW/0157). All participants provided written informed consent, and no compensation was provided to the participants. All research conforms to the principles of the Declaration of Helsinki.

### Study cohorts

#### MAFALDA cohort

A total of 74 individuals with obesity (*n* = 11 in the discovery cohort MAFALDA 1, and *n* = 63 in the replication cohort MAFALDA 2) (Supplementary Data 1) were included from the previously published Molecular Architecture of FAtty Liver Disease in individuals with obesity undergoing bAriatric surgery (MAFALDA) cohort^54,55^. For this study, we obtained visceral adipose tissue (VAT) biopsy samples around the gastric cardia, genotype, and phenotype data. The non-steatotic controls and MASLD and MASH cases were all individuals with morbid obesity (body mass index, BMI ≥ 35 kg/m^2^) undergoing bariatric surgery and recruited at Fondazione Policlinico Universitario Campus Bio-Medico, Rome, Italy^54,55^. Intraoperative liver biopsies were obtained and scored according to the NASH Clinical Research Network (NASH CRN) scoring system^56^. MASLD was determined by a steatosis grade≥1, and MASH was diagnosed by pathologists with a grade≥1 for steatosis, ballooning, and lobular inflammation^57^. The non-steatotic controls are individuals with obesity without MASH or MASLD. In MAFALDA 1, there are six cases with MASLD, four of whom have MASH, whereas the five controls have neither MASLD nor MASH, resulting in a total of 11 individuals (Supplementary Data 1). In MAFALDA 2, there are 44 cases with MASLD, 26 of whom have MASH, whereas the 19 controls have neither MASLD nor MASH, resulting in a total of 63 individuals (Supplementary Data 1). MAFALDA 1 and MAFALDA 2 comprise independent individuals with no overlap. Individuals were excluded if they had a history of alcohol abuse, viral hepatitis, or other causes of liver disease^58^.

#### KOBS cohort

A total of 59 individuals with severe obesity (BMI ≥ 35 kg/m^2^) undergoing bariatric surgery were included from the Kuopio OBesity Surgery study (KOBS) cohort^59,60^. For this study, we used subcutaneous adipose tissue (SAT) biopsy samples, genotype, and phenotype data. Individuals with obesity were recruited in the University of Eastern Finland and Kuopio University Hospital, Kuopio, Finland^61^.

#### RYSA cohort

For the adipocyte size investigations, we included 66 individuals with severe obesity (BMI ≥ 35 kg/m^2^) from the Roux-en-Y versus one-anastomosis gastric bypass (RYSA) cohort^62^, with existing VAT bulk RNA-seq data and adipocyte size estimates available, the latter measured as described previously^62^. These individuals with obesity undergoing bariatric surgery were recruited at the Helsinki University Hospital, Helsinki, Finland^62^.

Self-reported sex information was available for all individuals from the MAFALDA, KOBS, and RYSA cohorts and was cross-checked with the genetically inferred sex from the DNA-level genotype data.

#### UK biobank cohort

We used imputed genotype and phenotype data from the UK Biobank (UKB) cohort^63–65^. The UKB cohort includes over 500,000 individuals with detailed phenotype information, recruited from 2006 to 2010^63,64^. Phenotype data was collected at the time of recruitment and imaging data for a subset of individuals was obtained in follow-up evaluations^65^. Genotyping of variants was performed using either the Affymetrix or Applied Biosystems UK Biobank Axiom genotyping technology. Imputation was done with the Haplotype Reference Consortium and the merged UK10K and 1000 Genomes phase 3 reference panel^33,63^. All analyses were completed with unrelated individuals of European ancestry. Sex was determined based on self-reporting. Data from UKB was accessed under application 33934.

### Genotype data

MAFALDA genotype data was performed using the Illumina Global Screening Array (GSA)−24 v3.0 plus Multidisease Array (Illumina, San Diego, CA). Genotype quality control (QC) was done following the Liver Bible cohort^35,55^. Variants were imputed using Minimac4^66^ with the GRCh38 TOPMed reference genome panel^67,68^. Only imputed variants with an R^2^ > 0.3 were kept in all final analyses. For de-multiplexing of the snRNA-seq data, variants with a MAF < 5% were excluded.

KOBS genotype data were originally generated using the Infinium Global Screening Array-24 v1 (Illumina), as described previously^21^. Prior to imputation, we performed quality control, as described before^69^, to remove monomorphic, unmapped, and strand ambiguous SNPs and those that were not in Hardy-Weinberg equilibrium (HWE) with a *p* < 10^−6^ or missingness >2%. Similarly, as with the MAFALDA genotype data, imputation was conducted using Minimac4^66^ with the GRCh38 TOPMed reference genome panel^67,68^. We carried all downstream analysis with variants passing imputation quality control parameters, an R^2^ > 0.3 and HWE *p* < 10^−6^.

### VAT and SAT biopsy sample preparation and nuclei isolation for snRNA-seq

For the discovery cohort (MAFALDA 1, *n* = 11 individuals), we manually minced 300 mg per VAT biopsy sample, and no samples were pooled. All samples were isolated for nuclei separately using the 10x Genomics nuclei isolation protocol^21,70^. We took an aliquot of 10uL per sample to assess quality and quantity of nuclei. The nuclei in the aliquot were stained with Hoechst stain and counted using the Countess II FL Automated Cell Counter. Nuclei were then loaded into the 10x Chromium Chip and library construction was performed using the Single Cell 3’ v3.1 chemistry (Supplementary Data 49). All libraries were sequencing over two lanes of the NovaSeq 6000 S4, in which we targeted 50,000 reads per nucleus.

For the replication cohort (MAFALDA 2, *n* = 63 individuals), we created 8 batches that included a pool of 7–8 VAT samples per batch. We randomly assigned samples to batches to minimize associations with phenotypes of interest, including sex, age, BMI, MASLD status, and diabetic status. We took 50 mg per VAT biopsy sample for nuclei isolation. VAT samples require careful isolation of nuclei due to their tendency to experience lymphocyte infiltration^71^. Therefore, to ensure each sample had a diverse, substantial number of cell-types, we isolated nuclei in groups of two samples prior to pooling all samples into their respective batch. To increase nuclei yield, we used the Miltenyi Biotec gentleMACS tissue dissociator and followed the 10x Genomics nuclei isolation protocol^21,70^. Nuclei were aliquoted and DNA was stained with Hoechst stain allowing for quantification using the Countess II FL Automated Cell Counter. Following this, nuclei were then loaded into the 10x Chromium Chip and libraries were constructed using the Single Cell 3’ v3.1 chemistry (Supplementary Data 49). All libraries were equally distributed across two lanes and sequenced on the Novaseq X Plus 25B to target 70,000 reads per nucleus.

For the KOBS cohort (*n* = 59 individuals), we created 8 batches that included a pool of 7–8 SAT samples per batch. To prevent our results being confounded by significant batch effects, we randomly assigned the samples to the batches, similarly as in the MAFALDA 2. We took 100 mg from each sample prior to pooling the samples together for nuclei isolation. We used the Miltenyi Biotec gentleMACS tissue dissociator to increase nuclei yield and applied the 10x nuclei isolation protocol, as described before^21,70^. Nuclei were stained and quantified with the Hoechst stain and the Countess II FL Automated Cell Counter. We then loaded the nuclei into the 10x Chromium Chip and created libraries using the Single Cell 3’ v3.1 chemistry (Supplementary Data 49). Sequencing was done over two lanes of the Novaseq X Plus 25B where we targeted 70,000 reads per nucleus.

### Existing bulk VAT RNA-seq data

Libraries were prepared using the KAPA mRNA HyperPrep Kit and sequenced on the Illumina NovaSeq 6000, with an average sequence depth of 50-60 million reads per sample. We aligned reads against the human reference genome hg38 with GENCODE v42 annotations^72^ using STAR^73^ v.2.7.10b applying its two-pass procedure. Sample quality was assessed with FASTQC^74^ and RNA metrics were obtained with Picard Tools v2.25.5. Read counts were calculated using featureCounts^75^ v2.0.3.

### Processing and quality control of snRNA-seq data

FASTQ files were aligned using the alignment tool, STAR^73^ v2.7.10a, with parameter ‘--soloFeatures Gene GeneFull SJ Velocyto’ to accommodate snRNA-seq data. We used the GRCh38 human genome reference and GENCODE v42 filtered annotations^72^. Quality of FASTQ files and alignment output was assessed using FastQC^74^. In DIEM^76^, we utilized default parameters other than increasing the number of clusters in the k-means clustering step to 50 (k = 50)^69^ and using a UMI cutoff of 500 based on the knee bend plot. We manually removed clusters with high debris scores or those with top expressed genes being mitochondrial (MT) and ribosomal genes. Afterwards, we removed low-quality droplets with Seurat^77^ v5.0.0 using nFeatures≤200, UMI ≤ 500, and MT% ≥ 10. The data were then log-normalized and scaled using default parameters. Prior to clustering, principal component analysis (PCA) was performed and the top 30 principal components (PCs) were included in the ‘FindNeighbors’ function. The default resolution of 0.8 was used in the ‘FindClusters’ function in preparation for DecontX^78^. To remove contaminated reads, we used the raw data as background and the post-DIEM^76^ and post-clustered data as our input in DecontX^78^ (celda v1.16.1). We then removed the low-quality nuclei with nFeatures≤200, UMI ≤ 500, and MT% ≥ 10.

### Demultiplexing and removal of heterotypic doublets from snRNA-seq data

For both the MAFALDA 2 and KOBS, we used demuxlet^79^ v2 via popscle to demultiplex the samples within the batches. Employing the default parameters of demuxlet^79^ and the additional parameter of ‘--min-MQ 30’, we used genotype data to assign singlet droplets to the corresponding individuals. As samples from MAFALDA 1 were not pooled into batches, no demultiplexing was performed.

To ensure that no heterotypic doublets remained within the data, we ran DoubletFinder^80^ v2.0.3 on all three snRNA-seq data sets. For each respective sample or batch (depending on the data set), we selected a pN of 0.25 and an optimal pK value to calculate pANN and estimate the predicted number of doublets within the data. We then subset the data to only include singlets.

### Data integration and cell-type annotation in snRNA-seq data

Using the remaining droplets, we merged the samples or batches, respectively of each cohort, together. We then applied SingleR^81^ v2.2.0 to perform cell-type annotation by cell using the previous single nucleus and single cell adipose tissue atlas^19^ as a reference for VAT and SAT separately. Following this, we removed the nuclei that were determined as low quality or ambiguous cell-types. To investigate only expressed genes in our cell-type of interest, we subset our data to genes with a raw count of 3 in at least 3 nuclei^78^. We integrated on batch (or on sample) with Harmony^82^ v1.1.0 to remove residual batch effects and clustered the nuclei via the Louvain method using the first 40 principal components (PCs) and at a resolution of 0.5. Upon clustering, we used SingleR^81^ to annotate the clusters by cell-type using the adipose tissue atlas^19^ for plotting purposes.

### Subtyping of adipocytes and adipose stem and progenitor cells (ASPCs) from VAT and SAT snRNA-seq data

Following merging of batches or samples per data set, adipocytes and adipose stem and progenitor cells (ASPCs) were subset from each main data set. We kept genes with at least 3 raw counts in 3 nuclei^78^ and integrated on batch (or sample) with Harmony^82^ v1.1.0. For adipocytes, we performed clustering using the first 30 PCs at resolutions between 0.5–0.7. ASPCs were clustered using the first 20 PCs at resolutions 0.3 or 0.5. Adipocyte and ASPC subtypes were defined using SingleR^81^ v2.2.0 with adipocyte and ASPC subtypes from the previous adipose single cell atlas as reference^19^, respective of tissue type (VAT or SAT).

### Identification of marker genes in VAT adipocyte subtypes, VAT and SAT ASPC subtypes, and Louvain clusters

To identify marker genes in the adipocyte and ASPC subtypes and Louvain clusters, we employed ‘FindAllMarkers’ from Seurat^77^ v5.0.0. We used default parameters and only.pos=TRUE, min.pct=0.25, and logfc.threshold=0.25^83^. Marker genes for each subtype and Louvain cluster were defined as the genes identified in their respective subtype or cluster that passed a Bonferroni adjusted *p* value of 0.05 and are expressed at a log_2_FC > 0.25, similarly as in previous single cell RNA-seq studies^19,84–87^. These marker genes are generally expressed in all subtypes and clusters, but not to the same high degree as in their respective subtype or Louvain cluster.

### Pathway enrichment analysis of marker genes and gene sets

Next, we performed functional pathway enrichment analysis with the marker genes and gene sets using the R version of WebGestalt^88^ v0.4.6. We applied the ‘ORA’ enrichment method to find overrepresented enrichment of the marker genes and the gene sets within the Gene Ontology (GO) Biological Process database. For adipocytes, genes that had at least a raw count of three in three adipocytes or ASPCs^78^ were used as the background. For assessing the pathway enrichment of the subset of the predicted degree of adipocyte differentiation genes (*n* = 666 genes), we used all of the expressed genes that overlapped between the VAT adipocytes and the BRB-seq VAT adipogenesis data set^89^ as the background. For assessing the pathway enrichment of the hepatocytes differentially expressed (DE) by MASLD that were part of the predicted degree of hepatocyte differentiation gene set, we used all expressed genes in hepatocytes as the background. Significantly enriched pathways were determined by FDR < 0.05.

### Alignment and data integration of liver snRNA-seq data to estimate latent time in hepatocytes

Liver snRNA-seq data (*n* = 18 individuals with liver histology) were quality controlled and processed as described previously^26^. The liver snRNA-seq data^26^ comprise 13 individuals with MASLD, nine of whom have MASH, and five individuals with non-steatotic livers. Sex was determined based on self-reporting. To obtain the spliced and unspliced matrices for scVelo^24^, we aligned the fastq files using STAR^73^ v2.7.10a with the parameter ‘--soloFeatures Velocyto’ against the GRCh38 human genome reference^72^ matching Cell Ranger^90^ v.3.0.2. For downstream analysis, we used the published processed files and subset hepatocytes from the other main cell-types based upon key hepatocyte marker genes from PanglaoDB^91^. We then normalized and scaled the data with default parameters using Seurat^92^ v4.3.0. To remove batch effects, we integrated with Harmony^82^ v1.1.0 on sample and performed clustering using the first 30 PCs and a resolution 0.5 in Seurat^92^.

### Calculating module scores of subtype and zonation marker genes

To assess the presence of VAT adipocyte subtypes in MAFALDA 1, we first obtained the marker genes of each annotated subtype in MAFALDA 2. We then calculated modules scores in MAFALDA 1 using Seurat^77^ to highlight the presence of the VAT subtypes in the smaller cohort. For the liver zonation, we obtained the marker genes of the liver zones from a previously published paper^27^ and calculated module scores of these liver zonation marker genes in hepatocytes from the liver snRNA-seq data^26^. The scores were scaled from 0 to 1 and plotted.

### Alignment and gene quantification of bulk visceral ASPCs and differentiated adipocytes

Bulk RNA barcoding and sequencing (BRB-seq) was previously performed on seeded ASPCs and differentiated adipocytes from omentum biopsies^89^. We assessed the read quality using FastQC^74^, and then aligned the reads using STAR^73^ v2.7.10a with the two-pass method and default parameters. Alignment was done against the GRCh38 human genome reference and GENCODE v42 filtered annotations^72^. Following this, we used featureCounts^75^ from the subread package v2.1.0 and only kept uniquely mapped reads for downstream analysis. Technical metrics were obtained using Picard Tools v2.25.0.

### Estimating cell-type proportions in VAT bulk RNA-seq data from RYSA

To estimate cell-type proportions in VAT bulk RNA-seq data from the RYSA cohort, we used the marker gene-based method from BisqueRNA^93^ v1.0.5. Briefly, we Trimmed Mean of M-values (TMM) normalized and converted the counts to counts per million (CPM) with edgeR^94^ v3.42.4. Values were then log_2_-transformed after adding a prior count of 1. Next, we regressed out technical factors, including RNA integrity number (RIN), percent intronic bases, and median 3’ bias^70^, and then inverse normalized the data. For this analysis, we used the marker genes derived from the cell-types in MAFALDA 2 VAT snRNA-seq data.

### Gene set enrichment analysis in VAT bulk RNA-seq data from RYSA

Using the count data from the VAT bulk RNA-seq data in RYSA, we first filtered for the genes expressed in 90% of the samples^95^, resulting in 20,805 expressed genes. Then we TMM-normalized the counts in preparation for modeling the mean-variance with limma-voom^96^ v3.56.2. To identify the genes, the VAT expression of which is associated with the adipocyte diameter in RYSA, we first adjusted for RIN, percent intronic bases, median 3’ bias, sex, age, BMI, and cell-type proportions. Cell-type proportions included the five major cell-types: adipocytes, ASPCs, macrophages, endothelial cells, and T-cells (see above). For gene set enrichment analysis, we used fgsea^97^ v1.26.0 and provided gene sets in the pathway parameter.

The following VAT bulk expressed genes among the predicted degree of VAT adipocyte differentiation gene set were included for the pathway parameters: the 263 genes that are differentially expressed (DE) by MASLD or MASH; the 145 genes that are upregulated by MASLD or MASH; and the 118 genes that are downregulated by MASLD or MASH (Supplementary Data 42). Gene ranking was determined based on the effect size and -log10(*p*-value) from the topTable function in limma-voom^96^ v3.56.2. Default parameters were otherwise employed. Then we used the genes associated with the enrichment (*n* = 48 genes) for further downstream analysis described below.

### Correlating the first principal component of VAT expression data with adipocyte diameter

In preparation for principal component analysis (PCA), counts were TMM-normalized and transformed to CPMs. A prior count of 1 was added before performing log_2_ transformation. Normalized values were residualized for the same technical and biological factors and cell-type proportions as for the gene set enrichment analysis and then inverse normalized. Next, we subset the inverse normalized values to the genes of interest for PCA, respectively. Finally, we assessed the relationship between PC1 of VAT expression for each respective gene set and adipocyte diameter using Spearman’s rank-order correlation test.

### Identifying differentially expressed genes in BRB-sequencing data

Leveraging the previously published data set of human visceral ASPCs differentiated to adipocytes from the omentum^89^, we performed differential expression (DE) analysis between ASPCs (day 0) and differentiated ASPCs (day 14) using limma-voom^96^ v3.56.2. The DE analysis was performed on genes with a nonzero count in 90% of samples, resulting in 10,080 genes^95^. Counts were TMM normalized with edgeR^94^ v3.42.4 and used as input in the limma-voom^96^ v3.56.2 pipeline. In the model, we corrected for age, sex, percent uniquely mapped, and median 3’ bias and used false discovery rate (FDR) to correct for multiple testing. Significance was determined by an FDR < 0.05.

### Trajectory analysis within adipocytes and hepatocytes and determining putative genes associated with transcriptional activity

To obtain spliced and unspliced matrices from STAR^73^ v2.7.10a run, we included the parameter ‘--soloFeatures Velocyto’. The matrices were then added to the remaining adipocytes and hepatocytes passing quality control. The R objects were converted to anndata objects with the gene count matrices, spliced count matrices, and unspliced count matrices as individual layers. We first filtered the unspliced and spliced count matrices based upon the top 2000 variable genes that had at least a count of 20 in unspliced and spliced count matrices^24^. These genes were then normalized with ‘scv.pp.filter_and_normalize’ in scVelo^24^ v0.3.1. Moments of unspliced over spliced abundances (velocity) were computed with ‘scv.pp.moments’ from scVelo^24^, using the first 30 PCs, size 30 neighborhoods, and the Harmony embeddings to ensure the trajectory was not associated with batch effects for both adipocytes and hepatocytes. To better learn the transcriptional behavior, we applied the dynamical model from scVelo^24^ that employs a likelihood-based expectation-maximization framework to estimate gene phase trajectory. Default parameters were applied for all analysis with scVelo^24^ v0.3.1.

Latent time is estimated for each cell to predict what stage of differentiation it is in. This was determined with ‘scv.tl.latent_time’ applying default parameters from scVelo^24^ that identifies root and end cells based upon the Markov diffusion process. To compare our latent time values across disease conditions in adipocytes, we first regressed out batch, number of adipocytes per sample, age, sex, BMI, diabetic status, and fibrotic status and added residuals back to the mean latent time to re-establish data structure prior to using the two-sided Wilcoxon rank sum test. To prevent confounding by *PNPLA3* when comparing disease conditions, we also adjusted for genotypes of the MASLD-associated *PNPLA3* variant rs738409^25^ prior to adding residuals back to the mean latent time. When comparing sex, we performed a similar procedure except instead of including sex as a covariate, we corrected for the MASLD status. We employed blood platelet count as a phenotypic control trait. The same covariates, including sex and MASLD, were included in the model prior to adding the residuals back to the mean latent time. We determined significance across subtypes using the false discovery rate (FDR) < 0.05. For clarity, we refer to the adipocyte latent time gene set as the predicted degree of adipocyte differentiation gene set as well. This latent time gene set consists of all genes tested to estimate the trajectory by scVelo^24^.

Using the overlapping genes between the DE genes from the BRB-seq data set^89^ and our latent time gene set, we utilized the same pipeline above to obtain the latent time of this subset of genes in VAT adipocytes. We refer to this gene set as the subset of latent time gene set.

For the hepatocytes, we regressed out the number of hepatocytes per sample, age, sex, BMI, and fibrotic status and then added the residuals back to the mean latent time. Similarly to the adipocytes, we used the two-sided Wilcoxon rank sum test and determined significance by a *p* < 0.05. We refer to the gene set used to calculate the trajectory by scVelo^24^ in hepatocytes by the predicted degree of hepatocyte differentiation gene set and the hepatocyte latent time gene set.

To investigate genes associated with the latent time results, we employed ‘scv.tl.rank_velocity_genes’ with the genes that were used to create the trajectory and those that had a minimum Spearman correlation of 0.3 between spliced and unspliced counts per gene. Ranking was then performed by the MASLD status from scVelo^24^ to obtain a ranked gene list.

### Assessing associations between MASLD and clinical parameters

To determine whether clinical parameters are associated with MASLD, we ran a logistic regression model and corrected for sex. Insulin and glucose were also adjusted for the diabetic status. MASLD was binarized and used as the outcome. We adjusted for multiple testing with the Bonferroni correction, considering an adjusted *p* < 0.05 as significant. We then assessed the relationship with latent time (adjusted for covariates described above) and insulin using a linear regression model. Furthermore, blood platelet count was determined as a negative control by assessing the relationship between MASLD, MASH, and fibrosis and blood platelet count. We ran logistic regression models for each trait, respectively, and adjusted for age and sex.

### Assessing subtype proportions in adipocytes and ASPCs from VAT and SAT snRNA-seq data

We determined proportions of subtypes by calculating the number of each adipocyte subtype or ASPC subtype over the total number of adipocytes or ASPCs in each individual. For comparisons between the individuals with MASLD or MASH and individuals with non-steatotic livers, we took the residuals after regressing out batch, age, sex, BMI, diabetic status, and fibrotic status and performed inverse normalization. For sex, we did the same procedure except we regressed out the MASLD status instead of sex. Next, we performed the two-sided Wilcoxon rank sum test and used FDR to correct for multiple testing across the subtypes of adipocytes or ASPCs for each condition, respectively. Significance was determined by an FDR < 0.05.

### Correlating adipocyte subtypes with phenotypic traits in VAT and SAT snRNA-seq data

To assess the heterogeneity of VAT and SAT adipocytes and determine a putative correlative relationship between phenotypes, we applied ‘association.Seurat’ from the R version of Covarying Neighborhood Analysis^30^ (CNA) v0.0.99. For the CNA^30^ analysis of MASH, we adjusted for batch, age, sex, number of adipocytes per individual, diabetic status, and fibrotic status. For sex, we adjusted for batch, age, MASLD status, number of adipocytes per individual, diabetic status, and fibrotic status in MAFALDA. In KOBS, we adjusted for batch, age, MASLD status, number of adipocytes per individual, and diabetic status. To obtain significance estimates of the global heterogeneity testing, we ran 10,000 permutations for MASH and sex, respectively. The global p is used to test whether the null hypothesis (i.e., that there is no association between the neighborhood and the phenotypic trait) can be rejected. The phenotypic trait of interest in the cohort was permuted to obtain a null distribution for this analysis.

### Performing GWAS for MASLD traits

We performed genome-wide association studies (GWASs) for the continuous serum alanine transaminase (ALT) (*n* = 373,241), liver magnetic resonance imaging proton density fat fraction (PDFF) (*n* = 23,606), fatty liver index^98^ (FLI) (*n* = 372,830), and the binarized MASLD status using PDFF (*n* = 17,463) and binarized FLI (*n* = 275,467) in the unrelated European individuals of the UKB^63–65^. We used BOLT-LMM^99^ v2.3.6 to conduct the GWASs in the UKB, while quantile-normalizing the continuous outcomes and adjusting for age at measurement, age at measurement^2^, sex, genotyping array, testing center, and top 20 genetic PCs. For the binarized MASLD outcome using PDFF, we identified individuals with PDFF > = 5% as cases^100,101^, and individuals with PDFF less than the median (3.1%) as controls. To binarize FLI, we defined the MASLD cases as individuals with FLI > = 60 and controls as individuals with FLI < 30^98^. In the ALT, MASLD status, and FLI GWASs, we additionally adjusted for weekly alcohol intake, which was derived by converting the reported red wine, champagne plus white wine, beer plus cider, spirits, fortified wine, other alcoholic drink intakes to average grams per day and taking the sum^102,103^. Additionally, we also conducted a separate GWAS for ALT without adjusting for alcohol intake (*n* = 257,497), in which we instead omitted the individuals with heavy alcohol intake, defined as average daily alcohol intake exceeding 20 grams for females, and 30 grams for males^100,101^. The continuous PDFF GWAS also included BMI during imaging as a covariate and excluded individuals with heavy alcohol use.

### Assessing enrichment of gene sets with MAGMA

To determine whether variants within or surrounding the genes (within 10 kb) in the gene sets are enriched for MASLD GWAS traits, we ran MAGMA^41^ (Multi-marker Analysis of GenoMic Annotation) v1.10. The MASLD GWAS traits summary statistics were used with the LD reference panel of individuals of European descent from the 1000 Genomes Project^104^.

### Estimating partitioned heritability of gene sets for MASLD GWAS traits

Partitioned heritability of MASLD GWAS traits from annotations from gene sets of interest was assessed using LD score regression (LDSC)^42^ v1.0.1 on unrelated individuals of European ancestry in the UKB. Significant enrichment of a trait was determined by an enrichment *p* < 0.05.

### Identifying differential expressed (DE) genes in VAT adipocytes, VAT and SAT ASPCs, and liver hepatocytes by MASLD, MASH, and sex

To obtain differentially expressed (DE) genes by MASLD, MASH and sex in VAT adipocytes, we used ‘FindMarkers’ from Seurat^77^ v5.0.0 and employed the hurdle model from MAST^43^ v1.26.0. For ASPCs, the DE analyses by MASLD and sex in VAT were performed in a similar way as in adipocytes using ‘FindMarkers’ from Seurat^77^ v5.0.0 and the hurdle model from MAST^43^. For MASLD and MASH, we adjusted for the number of nuclei, batch, age, sex, fibrotic and diabetic status, and BMI. For sex, we adjusted for the same covariates other than sex, and in its place included MASLD status. In the DE analysis by MASLD in SAT, we adjusted for number of nuclei, batch, age, sex, diabetic status, and BMI. For sex, we corrected for the same covariates, excluding sex that was replaced by the MASLD status.

Leveraging the hepatocyte snRNA-seq data^26^, we obtained DE genes by MASLD as described above for adipocytes and ASPCs. In hepatocytes, we adjusted for the number of nuclei, age, sex, BMI, and fibrosis status. Significance in all DE analyses was determined based upon a Bonferroni adjusted *p* < 0.05 and an absolute log_2_FC > 0.25.

### Determining the overlap between the DE genes and the predicted degree of adipocyte differentiation gene set

We employed a Fisher’s exact test to assess the significance of the overlap between the VAT DE genes by MASLD, MASH, and sex and the predicted degree of adipocyte differentiation gene set using GeneOverlap v1.36.0. This analysis was done for each group of the DE genes by condition. The genes that we included for the genome size parameter comprised the expressed adipocyte genes, defined as the genes with at least a raw count of three in three nuclei^78^. For the overlap analysis between the DE genes of the BRB-seq data set^89^ and the genes of our gene set, we also used the Fisher’s exact test to determine significance from GeneOverlap v1.36.0. The genome size included the shared genes tested in the DE analysis and expressed in VAT adipocytes.

### VAT adipocyte *cis*-eQTL analysis

We performed cell-type-level *cis*-expression quantitative trait loci (*cis*-eQTL) analysis in VAT adipocytes using Matrix eQTL^44^ v2.3. We selected the tested expressed genes based on the following parameters: a pseudocount>0.1, transcripts per million (TPM) in at least 20% of the samples, and raw counts>6 in at least 20% of samples^105^. Pseudocount matrices were constructed of the 11,954 genes passing these criteria, and the counts were TMM normalized to CPMs^106^ and inverse normalized across the samples. We further filtered the testing to the imputed and post-QC SNPs with a MAF > 10% in Italians in the UKB, resulting in 4,317,408 tested variants. In the *cis*-eQTL model, we included 10 genetic PCs, the number of nuclei in adipocytes per individual, and 16 expression PCs to capture potential hidden confounders in the expression data. We empirically determined the number of expression PCs included in the final model by evaluating the number of eGenes discovered compared to the number of PCs included^107^, and defined significant *cis*-eQTLs using an FDR < 0.05.

### Statistics & reproducibility

No statistical method was used to predetermine sample size; however, the single cell level sample sizes of the main cohorts are larger than or similar to those reported in previous cell-type level SAT and VAT studies^19,53,108^. No data were excluded from the analyses in MAFALDA 1, MAFALDA 2, or KOBS. Related individuals were excluded from the UKB. As this is an observational study, no randomization or blinding was performed. We used VAT snRNA-sequencing to examine adipocyte latent time results from a discovery cohort of 11 individuals, referred to as MAFALDA 1. We validated the adipocyte latent time results of MAFALDA 1 in a cohort of 63 independent individuals, referred to as MAFALDA 2. We performed all other analyses in the larger cohort of 63 independent individuals, MAFALDA 2, and did not perform further analyses in the smaller MAFALDA 1. Results reported from the UKB GWAS analyses were not replicated due to the unprecedented large sample size of the cohort. Statistical significance was assessed using empirical permutation, non-parametric tests, or parametric tests when possible.

### Reporting summary

Further information on research design is available in the Nature Portfolio Reporting Summary linked to this article.

## Supplementary information

Supplementary Information

Description Of Additional Supplementary File

Supplementary Data 1-49

Reporting summary

Transparent Peer Review file

## Source data

Source data

## Data Availability

The MAFALDA 1 VAT snRNA-seq data are available in the NIH Gene Expression Omnibus (GEO) database under accession code GSE302702. The MAFALDA 2 VAT snRNA-seq data are available in the NIH GEO under accession GSE302599. The KOBS SAT snRNA-seq data are available in the NIH GEO under accession GSE302701. The liver snRNA-seq data^26^ are available in the NIH GEO under accession GSE244832. The SAT and VAT reference snRNA-seq data from Emont et al.^19^ are available for downloading in the Single Cell Portal under study number SCP1376. The bulk RNA-sequencing data set of human visceral ASPCs differentiated to adipocytes^89^ are publicly available in EMBL’s European Bioinformatics Institute BioStudies database under ArrayExpress E-MTAB-12898. Data from the UK Biobank were used in this study under UK Biobank Application Number 33934. UK Biobank data are available for bona fide researchers through the application process (https://www.ukbiobank.ac.uk/learn-more-about-ukbiobank/contact-us). Source data for all figures is publicly available at Zenodo^109^ (https://zenodo.org/records/20127417). Source data are provided with this paper.

## Code availability

No custom code was employed and all codes used for analyses were from their original publications.
