# Supplementary material for: Decreased degree of adipocyte differentiation in visceral adipose tissue contributes to metabolic dysfunction-associated steatotic liver disease
Source: Nat Commun. 2026 Jun 3;17:7101. doi: 10.1038/s41467-026-73660-6 (PMC13392065; doi:10.1038/s41467-026-73660-6)
Supplement: Supplementary file 1 — Supplementary Information [file 41467_2026_73660_MOESM1_ESM.pdf]

Decreased degree of adipocyte differentiation in visceral adipose tissue contributes to metabolic dysfunction-associated steatotic liver disease

Kyla Z. Gelev[1], Seung Hyuk T. Lee[1], Marcus Alvarez[1], Rosellina M. Mancina[2,3,4,5], Federica Tavaglione[5,6], Oveis Jamialahmadi[3,4], Umberto Vespasiani-Gentilucci[5,7], Asha Kar[1,8], Zitian Wang[1,8], Dorota Kaminska[9,10], Minna U. Kaikkonen[11], Ville Männistö[12,13], Sini Heinonen[14,15], Tuure Saarinen[16], Anne Juuti[16], Kirsi H. Pietiläinen[15,17], Jussi Pihlajamäki[18,19]*, Stefano Romeo[3,4,20,21,22] *, Päivi Pajukanta[1,8,23]*

[1]Department of Human Genetics, David Geffen School of Medicine at UCLA, Los Angeles, CA
[2]Department of Life Science, Health, and Health Professions, Link Campus University, Rome, Italy
[3]Centre for Reproduction, Metabolism and Molecular medicine (CeRM), Department of Medicine (H7), Karolinska Institute, Huddinge, Sweden
[4]Department of Molecular and Clinical Medicine, University of Gothenburg, Gothenburg, Sweden
[5]Research Unit of Clinical Medicine and Hepatology, Department of Medicine and Surgery, Università Campus Bio-Medico di Roma, Rome, Italy
[6]MASLD Research Center, Division of Gastroenterology and Hepatology, University of California at San Diego, La Jolla, California, United States
[7]Operative Unit of Clinical Medicine and Hepatology, Fondazione Policlinico Universitario Campus Bio-Medico, Rome, Italy
[8]Bioinformatics Interdepartmental Program, UCLA, Los Angeles, CA
[9]Division of Cardiology, Department of Medicine, UCLA, Los Angeles, CA
[10]Institute of Public Health and Clinical Nutrition, University of Eastern Finland, Kuopio, Finland
[11]A. I. Virtanen Institute for Molecular Sciences, University of Eastern Finland, Kuopio, Finland
[12]Institute of Clinical Medicine, Internal Medicine, University of Eastern Finland, Kuopio, Finland
[13]Department of Internal Medicine, Kuopio University Hospital, Kuopio, Finland
[14]Department of Internal Medicine and Rehabilitation, Helsinki University Hospital, Helsinki, Finland
[15]Obesity Research Unit, Research Program for Clinical and Molecular Metabolism, Faculty of Medicine, University of Helsinki, Helsinki, Finland
[16]Department of Abdominal Surgery, Abdominal Center, Helsinki University Hospital and University of Helsinki, Helsinki, Finland
[17]Healthy Weight Hub, Abdominal Center, Helsinki University Hospital and University of Helsinki, Helsinki, Finland
[18]Institute of Public Health and Clinical Nutrition, University of Eastern Finland, Kuopio, Finland

[19]Department of Medicine, Endocrinology and Clinical Nutrition, Kuopio University Hospital, Kuopio, Finland
[20]Department of Endocrinology, Karolinska University Hospital, Huddinge, Sweden
[21]Department of Medical and Surgical Sciences, Magna Graecia University, Catanzaro, Italy
[22]Department of Cardiology, Sahlgrenska University Hospital, Gothenburg, Sweden
[23]Institute for Precision Health, David Geffen School of Medicine at UCLA, Los Angeles, CA

*Corresponding authors: jussi.pihlajamaki@uef.fi, stefano.romeo@ki.se, and PPajukanta@mednet.ucla.edu

Supplementary Notes

1.1 Clinical parameters associated with MASLD and latent time

1.2 The predicted degree of SAT adipocyte differentiation is decreased in individuals with MASLD and in males

1.3 Sex effect on adipocyte subtype proportions in VAT

1.4 Sex impacts VAT and SAT adipose stem and progenitor cells (ASPCs)

1.5 Adjusting for PNPLA3 genotype status does not impact latent time results in VAT and SAT

1.6 Subset of VAT latent time genes consistently exhibits a lower latent time in adipocytes from the individuals with MASLD than without MASLD

1.7 The first principal component of upregulated genes by MASLD or MASH in the VAT bulk expression data is associated with adipocyte size

Supplementary Figures

Supplementary References

Supplementary Notes

1.1 Clinical parameters associated with MASLD and latent time

We also assessed whether clinical parameters were associated with MASLD and the latent time (Supplementary Data 6). We found that after multiple test correction, only insulin remained significantly associated with MASLD. Considering that insulin was the only clinical parameter associated with MASLD, we ran a linear regression analysis to assess the relationship between insulin and the latent time. We found that insulin was inversely associated with the latent time (beta=-0.051, p-value=$2.67x10^{-8}$), i.e., higher insulin level is associated with a lower latent time, in line with the MASLD and MASH findings. This indicates that insulin levels also associate with the latent time.

1.2 The predicted degree of SAT adipocyte differentiation is decreased in individuals with MASLD and in males

To investigate SAT adipocyte differentiation, we performed snRNA-seq on SAT biopsies in KOBS (n=59). We first conducted QC (see Methods) (Supplementary Fig. 3) and then clustered and annotated the SAT cell-types (Supplementary Fig. 4a). We subset the data to SAT adipocytes and performed re-clustering and annotation on their subtypes using the previous SAT single cell atlas[1] as a reference (Supplementary Fig. 4b). To determine whether the latent time is also linked to MASLD in SAT adipocytes and their subtypes, we employed the dynamical model from scVelo[2] to obtain latent time values in SAT adipocytes (Supplementary Fig. 4c). We were unable to assess MASH in KOBS due to sample size (n=3). In individuals with MASLD, we identified a lower latent time (p=$1x10^{-5}$) in all SAT adipocytes (Supplementary Fig. 4d, Supplementary Data 7).

Subsequently, to determine whether some SAT adipocyte subtypes are linked to this observed difference in the latent time in all SAT adipocytes, we performed comparisons across the SAT adipocyte subtypes between MASLD and the control group. Individuals with MASLD had a modest reduction in the latent time in adipocyte subtype hAd1 (FDR=0.0013) (Supplementary Fig. 4e, Supplementary Data 8). However, we did not find differences in SAT adipocyte subtype proportions by MASLD (Supplementary Fig. 5).

We next investigated how sex contributes to the latent time in SAT adipocytes. We found a lower latent time in males than females ($p=1.4\times10^{-162}$) (Supplementary Fig. 6a, Supplementary Data 9). We further identified that all three SAT adipocyte subtypes were different in proportions by sex ($FDR=9.2\times10^{-4}$ for hAd1, FDR=0.0025 for hAd2, $FDR=9.2\times10^{-6}$ for hAd4) (Supplementary Fig. 6b). Both adipocyte subtypes, hAd1 and hAd2, had greater proportions in males than females, while hAd4 had lower proportions in males than in females (Supplementary Fig. 6b). Moreover, we also observed that there are differences in the latent times across all adipocyte subtypes between sex (FDR=0.041 for hAd1, $FDR=2.4\times10^{-41}$ for hAd2, $FDR=1.1\times10^{-4}$ for hAd4). Both hAd2 and hAd4 exhibited a lower latent time in the males, while hAd1 showed a higher latent time in males (Supplementary Fig. 6c, Supplementary Data 10). Further examining this sexual dimorphism in SAT, we identified a dramatic (global permuted p=0.0001) association of sex with SAT adipocyte heterogeneity (Supplementary Fig. 6d). Collectively, this indicates that sex has a profound effect on the predicted degree of SAT adipocyte differentiation.

1.3 Sex effect on adipocyte subtype proportions in VAT

Considering the sexual dimorphism of VAT[3,4] and that the VAT data comprises both males and females (Supplementary Fig. 7a), we investigated whether sex is associated with VAT adipocyte heterogeneity. First, to assess the sex effect on the adipocyte latent time, we evaluated the latent time of each group. We observed a lower latent time in males ($p=9.7x10^{-29}$) after adjusting for covariates (Supplementary Fig. 7b, Supplementary Data 11). Comparing the adipocyte subtype proportions between males and females using the two-sided Wilcoxon rank sum test (see Methods), we identified a greater difference in proportions of the adipocyte subtype hAd1 in males versus females (FDR=0.0033) (Supplementary Fig. 7c). Following this, we tested the latent time in a more granular resolution across the VAT adipocyte subtypes. We found that multiple VAT adipocyte subtypes exhibited (FDR<0.05) differences in latent time between males and females (Supplementary Fig. 7d, Supplementary Data 12). To further investigate the relationship between subtypes and sex, we performed an association analysis using CNA[5]. We found that sex is linked to the heterogeneity of VAT adipocytes (global p=0.0001) and that the male sex was highly correlated with specific regions and cell states of VAT adipocytes, more specifically, hAd1 (Supplementary Fig. 7e). This demonstrates that sex has a profound effect on adipocyte subtype diversity and contributes to VAT differentiation and function.

1.4 Sex impacts VAT and SAT adipose stem and progenitor cells (ASPCs)

Although we primarily focused on adipocytes due their known heterogeneity and key role in lipid storage[6], we also performed an investigation into adipose stem and progenitor cells (ASPCs) to better understand the influence of MASLD and sex on adipose tissue. In our analysis of VAT and SAT ASPCs, we did not detect VAT or SAT ASPC subtype proportions to differ by the MASLD status or sex (Supplementary Fig. 8). However, in the VAT and SAT ASPC DE

analyses, we found 261 DE genes by MASLD and 123 DE genes by sex in VAT (Supplementary Data 13), and 158 DE genes by MASLD and 349 DE genes by sex in SAT (Supplementary Data 14), respectively. To understand their biological processes, we tested the identified ASPC MASLD and sex DE genes for functional pathway enrichments. In VAT, the upregulated DE genes by MASLD were enriched, for example, for nucleotide-sugar biosynthesis process, complement activation, and regulation of inflammatory responses (Supplementary Fig. 9, Supplementary Data 15), underscoring a potentially pro-inflammatory microenvironment for adipogenesis. In SAT, upregulated genes by MASLD were overrepresented in responses to growth factors, while the downregulated genes by MASLD were enriched for positive regulation of fatty acids (Supplementary Fig. 9, Supplementary Data 15). The combination of these pathways potentially indicates the inhibition of adipogenesis in SAT. Among the VAT DE genes by sex, the upregulated genes in females were enriched for collagen processes and extracellular matrix organization, suggesting a greater impact on adipocyte differentiation in females than males (Supplementary Fig. 10, Supplementary Data 16). These were similar pathways as seen in the upregulated genes in females for SAT as well (Supplementary Fig. 10, Supplementary Data 16).

We also assessed whether the marker genes of the VAT and SAT ASPC subtypes differed by sex, similarly as in a previously published animal study, which investigated mouse ASPCs and their functional differences by sex[7]. In VAT, only 13 DE genes by sex overlapped with the marker genes of the VAT hASPC2 subtype, 15 genes overlapped with the marker genes of the VAT hASPC3 subtype, and three genes overlapped with the marker genes of the VAT hASPC4 subtype, respectively (Supplementary Data 17). However, we did find there to be a Louvain

cluster within the VAT ASPC subtypes that is enriched for sex-specific pathways, such as ovulation cycle and sex differentiation (Supplementary Data 18, Supplementary Fig. 11, Supplementary Data 19), suggesting the influence of sex in VAT ASPCs as well. Furthermore, the presence of these pathways also highlights the potential role of sex hormones in modulating human VAT ASPCs. In SAT, 54 genes DE by sex overlapped with the marker genes of the SAT hASPC4 subtype and 90 genes DE by sex overlapped with the marker genes of the SAT hASPC5 subtype (Supplementary Data 20). These 90 marker genes in the SAT hASPC5 subtype are enriched for collagen processes and extracellular matrix and structure (Supplementary Fig. 12, Supplementary Data 21), implicating modulated adipogenesis by sex in humans. This larger number of the sex DE genes in SAT ASPC subtypes supports the other SAT findings (Supplementary Fig. 6), showing that SAT is greatly impacted by sex.

Taken together, we saw larger proportions of subtype hAd1 in males. This implicates a potential sex effect on the predicted degree of adipocyte differentiation. Unlike in VAT, the SAT subtype proportions were more prominently associated with sex. While SAT also showed a difference in adipocyte latent time by MASLD, this did not carry into the adipocyte subtype proportion differences of hAd2. Therefore, this is consistent with the notion that VAT remodeling is more consequential to MASLD in comparison to remodeling in SAT. In ASPCs, we did not detect major differences in the proportions of their subtypes by MASLD or sex. However, we did observe some underlying sex effects in both VAT and SAT, in line with previous mouse studies[7,8]. This suggests hormonal influences on the microenvironment affecting adipogenesis

1.5 Adjusting for *PNPLA3* genotype status does not impact latent time results in VAT and SAT

As *PNPLA3* is part of the VAT latent time gene set, we also adjusted for the known MASLD variant, *PNPLA3* rs738409[9] to avoid possible confounding. In this analysis, the individuals with MASLD still showed a lower latent time ($p=3.1x10^{-36}$) in comparison to the individuals without MASLD (Supplementary Fig. 13, Supplementary Data 22). This was also true when comparing the individuals with MASH to the individuals without MASLD ($p=1.7x10^{-27}$) (Supplementary Fig. 13, Supplementary Data 23). In SAT, when correcting for the genotype groups of the MASLD-associated *PNPLA3* variant, rs738409[9], we still observed a lower latent time in the individuals with MASLD when compared to the individuals without MASLD ($p=1.1x10^{-5}$) (Supplementary Fig. 14, Supplementary Data 24).

1.6 Subset of VAT latent time genes consistently exhibits a lower latent time in adipocytes from the individuals with MASLD than without MASLD

We identified genes differentially expressed (DE) during VAT adipogenesis, leveraging a previously published BRB-seq data set of human visceral ASPCs differentiated to adipocytes from the omentum[10] (Supplementary Data 27). Then, we tested for the overlap (see Methods) between these genes DE during VAT adipogenesis and the predicted degree of adipocyte differentiation (i.e., adipocyte latent time) gene set, resulting in 666 overlapping genes (overlap p=0.002). Taking those 666 genes, we re-performed the analysis with scVelo[2] and found similar significant results as with the full gene set (2,000 genes) (Supplementary Fig. 15, Supplementary Data 28, 29). In more detail, the individuals with MASLD had a lower latent time when compared to the individuals without MASLD ($p=4.9x10^{-31}$). This lower latent time was also

observed in the individuals with MASH in comparison to the individuals without MASLD ($p=2.1x10^{-14}$). Furthermore, these 666 genes, referred to as the subset of predicted degree of adipocyte differentiation (i.e. adipocyte latent time) gene set are enriched for pathways of morphogenesis, development, and activin receptor signaling (Supplementary Data 30).

1.7 The first principal component of upregulated genes by MASLD or MASH in the VAT bulk expression data is associated with adipocyte size

To further investigate this finding, we then built the first principal component (PC1) of the VAT bulk expression of the genes upregulated by MASLD or MASH and contributing most to the enrichment (n=48 genes) (Supplementary Data 43) (for selection of these genes, see Methods). Next, we tested this PC1 for association with adipocyte size in the RYSA cohort. We identified a negative trend (rho=-0.242, p-value=0.057) between PC1 of the VAT bulk expression (n=48 genes) and the adipocyte diameter in RYSA. Given that only bulk VAT RNA-seq data are available in RYSA, we then focused more on adipocyte expression by testing the adipocyte marker genes among the 48 genes (n=13 genes, Supplementary Data 43) and found that there is also a significant negative correlation (rho=-0.263, p-value=0.039) between PC1 of their VAT bulk expression and the adipocyte diameter in RYSA. Finally, when we investigated the marker genes of VAT adipocyte subtype hAd1 among the 48 genes (n=14 genes, Supplementary Data 43), we observed that there is a significant negative correlation (rho=-0.31, p-value=0.013) between PC1 of their VAT bulk expression and the adipocyte diameter in RYSA. This coincides with our findings that the latent time in hAd1 is lower (FDR<0.05) in the individuals with MASLD livers vs non-steatotic livers (Fig. 3c, Supplementary Data 36) and that the marker genes of the hAd1 adipocyte subtype are enriched for preadipocyte specific pathways (Fig. 3f,

Supplementary Data 40), suggesting them to be earlier in their differentiation. Overall, these new adipocyte diameter results suggest that the MASLD DE genes predicting the differentiation degree are also linked to smaller adipocyte size.

Supplementary Figures

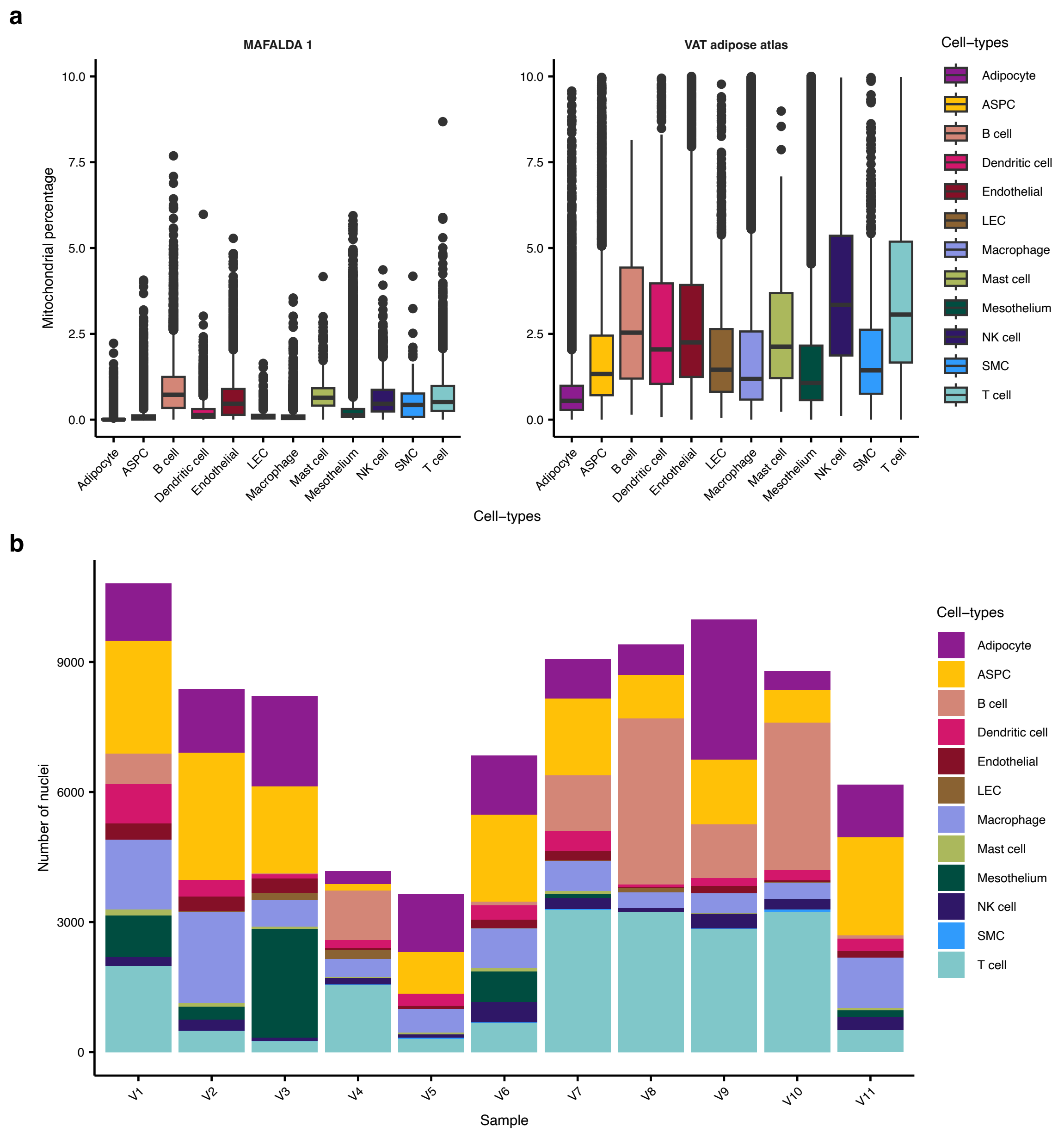

Supplementary Figure 1: Final nuclei after quality control pipeline in MAFALDA 1 (n=11). (a) Mitochondrial percentages across cell-types in MAFALDA 1 are comparable to those in the visceral adipose tissue atlas (n=10) by Emont et al.[1] **(b)** Stacked barplot displaying number of nuclei per sample (n=11 samples), colored by cell-types. Abbreviations: ASPC indicates adipose stem and progenitor cells; LEC, lymphatic endothelial cells; NK cells, natural killer cells, and SMC, (vascular) smooth muscle cell. In panel **a**, data are represented as boxplots with whisker end points indicating the 1.5x interquartile range. The upper and lower bounds of the boxes show the 25th and 75th percentiles, and the middle lines depict the median values. Outliers are indicated by dots. Source data are provided as a Source Data file.

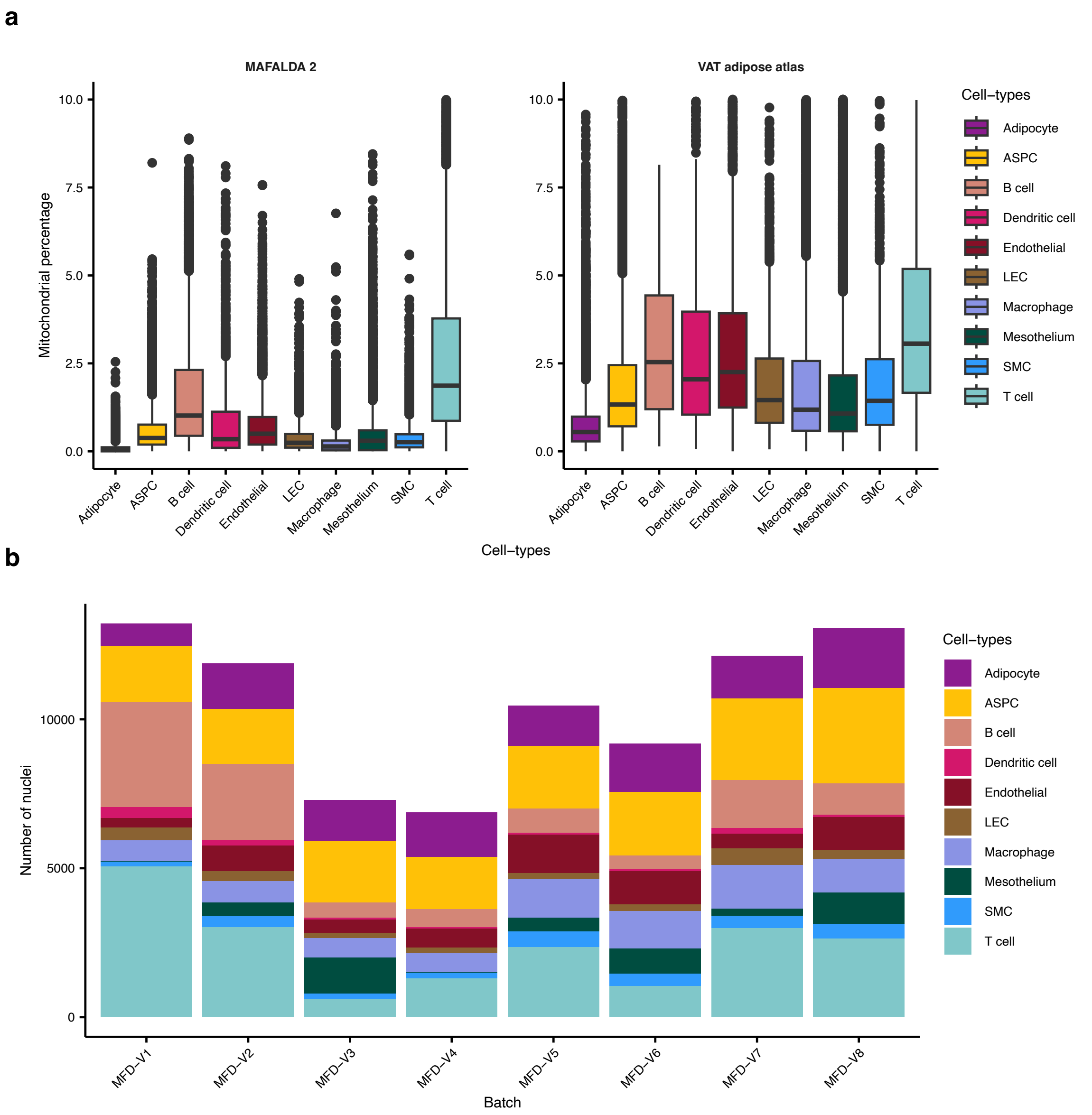

Supplementary Figure 2: Final nuclei after quality control pipeline in MAFALDA 2 (n=63). **(a)** Mitochondrial percentages across cell-types in MAFALDA 2 are comparable to those in the visceral adipose tissue atlas (n=10) by Emont et al.[1] **(b)** Stacked barplot showcasing number of nuclei per batch (n=63), colored by cell-types. Abbreviations: ASPC indicates adipose stem and progenitor cells; SMC, (vascular) smooth muscle cell; and LEC, lymphatic endothelial cells. In panel **a** data shown as boxplots with whisker end points indicating the 1.5x interquartile range, upper and lower bounds showing the first and third quarter percentiles, and the middle lines are the median values. Outliers are the singular dots. Source data are provided as a Source Data file.

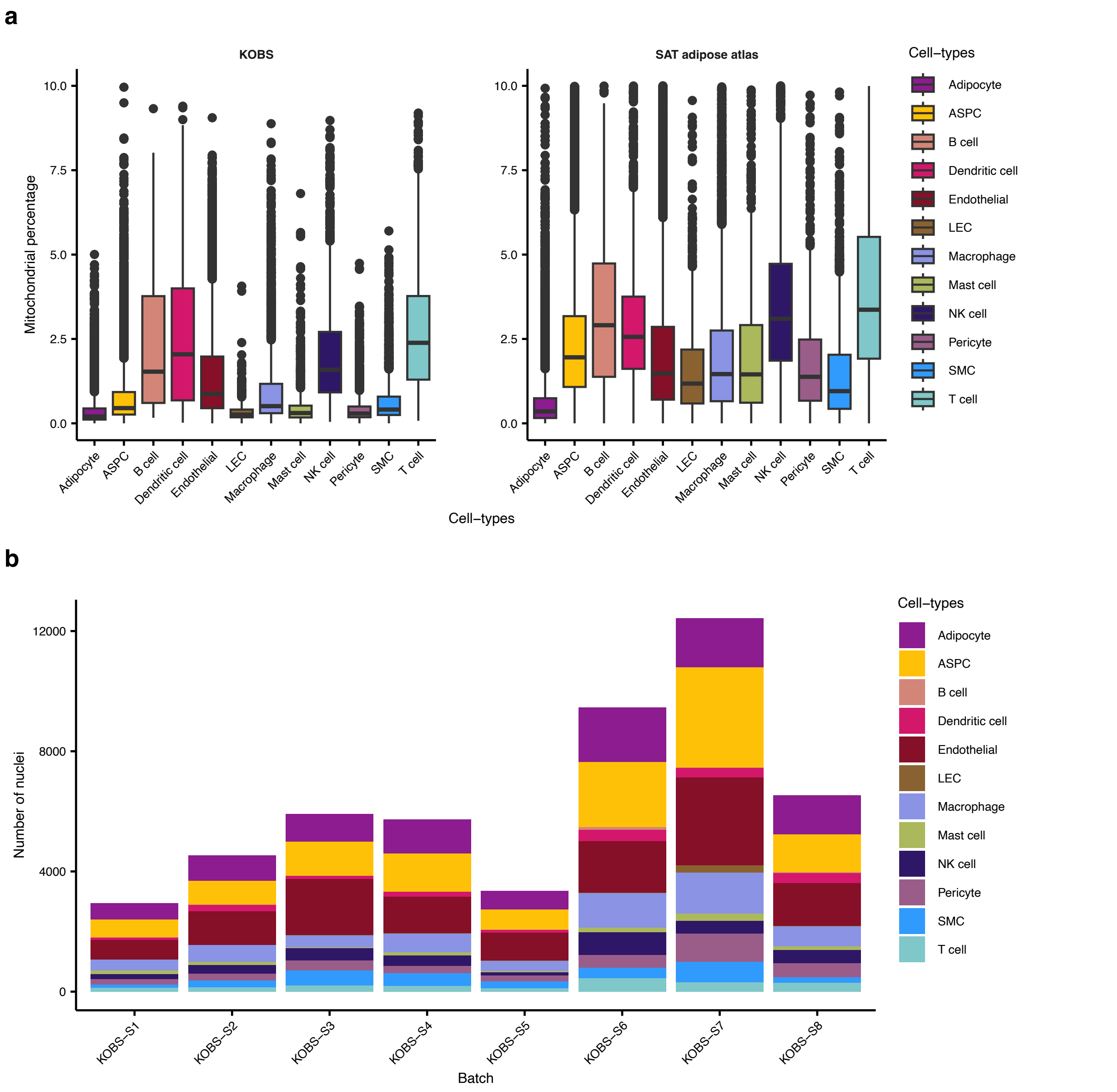

Supplementary Figure 3: Final nuclei after quality control pipeline in KOBS (n=59). (a) Mitochondrial percentages across cell-types in KOBS are comparable to those in the subcutaneous adipose tissue atlas (n=22) by Emont et al.[1] are comparable. **(b)** Stacked barplot showing number of nuclei per batch (n=59), colored by cell-types. Abbreviations: ASPC indicates adipose stem and progenitor cells; LEC, lymphatic endothelial cells; NK cells, natural killer cells, and SMC, (vascular) smooth muscle cell. Boxplots are used to showcase the data. Whisker end points indicate the 1.5x interquartile range with upper and lower bounds showing the 25th and 75th percentiles. The median values are depicted with the middle lines and outliers are shown by the singular dots. Source data are provided as a Source Data file.

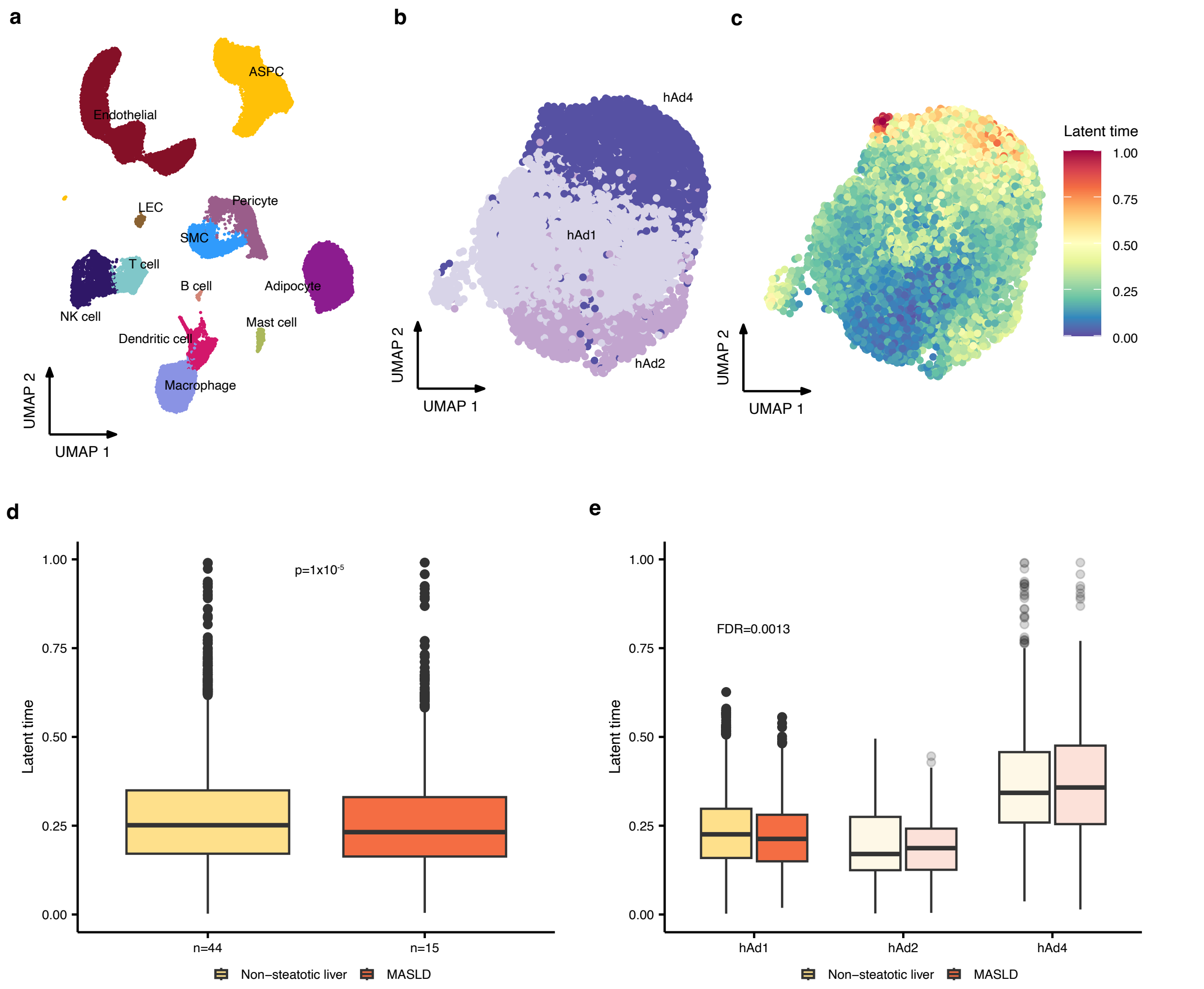

Supplementary Figure 4: SAT uncovers that adipocyte latent time and subtype proportions differ by MASLD. (a) Uniform Manifold Approximation and Projection (UMAP) visualization of 50,869 nuclei from the subcutaneous adipose tissue (SAT) snRNA-seq data in the extension data set (n=59, KOBS). **(b)** UMAP visualization of adipocytes, colored by subtype. **(c)** UMAP visualization of adipocytes, colored by the latent time values obtained from scVelo[2] dynamic model, blue indicating an earlier latent time and red indicating a later latent time. **(d)** Boxplots showing the latent time difference in adipocytes between the liver histology-based groups using the two-sided Wilcoxon rank sum test. Latent time was adjusted for age, sex, batch, number of nuclei, body mass index (BMI), presence of liver fibrosis, and presence of diabetes. Boxplots show the lower latent time in individuals with MASLD (n=15) when compared to the individuals with non-steatotic livers (n=44). **(e)** Boxplots show the latent time difference in adipocytes between the liver histology-based MASLD groups across subtypes by pairwise comparisons using the two-sided Wilcoxon rank sum test. In the boxplots, only subtype hAd1 has a lower latent time (FDR<0.05) in the individuals with MASLD (n=15) when compared to the individuals with non-steatotic livers (n=44). Abbreviations: SAT indicates subcutaneous adipose tissue; MASLD, metabolic dysfunction-associated steatotic liver disease; MASH, metabolic dysfunction-associated steatohepatitis; SMC, (vascular) smooth muscle cell; ASPC, adipose stem

and progenitor cells; LEC, lymphatic endothelial cells; NK cells, natural killer cell; hAd, human adipocyte subtype; and BMI, body mass index. Panels **d** and **e** feature the boxplots to represent latent time values. Boxplots show the median latent time values as the middle line, with the values ranging from the 25th to 75th percentiles. The whiskers extend to include 1.5x the interquartile range and the outliers are shown as dots. Shading in panel **e** shows the significant adipocyte subtypes. Source data are provided as a Source Data file.

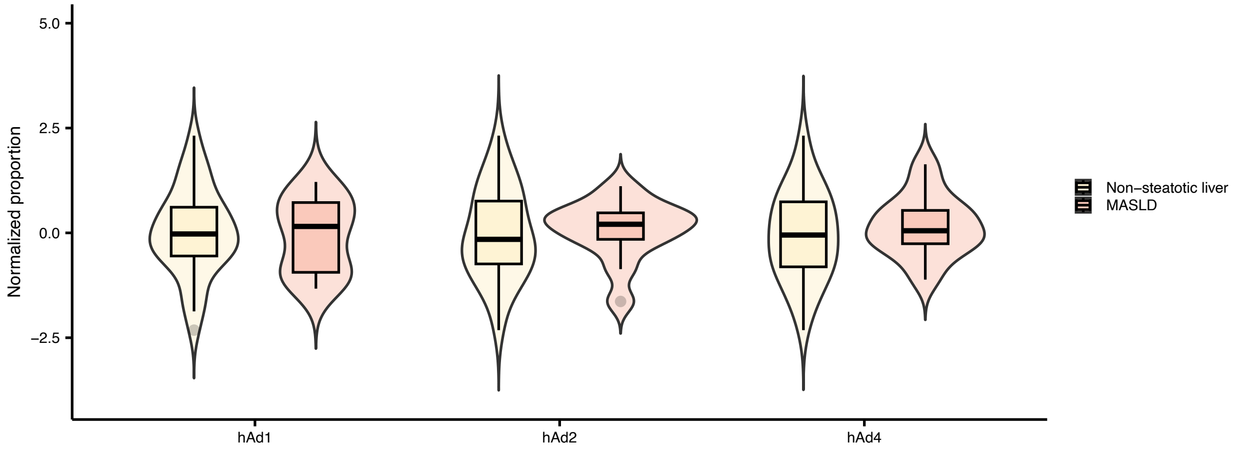

Supplementary Figure 5: No significant differences were observed in the SAT adipocyte subtype proportions between the individuals with MASLD and without MASLD in KOBS. Violin plots show that the normalized proportions of SAT adipocyte subtypes do not differ between the individuals with MASLD (n=15) and the individuals with non-steatotic livers (n=44) in KOBS, determined by pairwise comparisons using the two-sided Wilcoxon rank sum test and correcting for multiple testing using false discovery rate (FDR) (FDR<0.05). Abbreviations: SAT indicates subcutaneous adipose tissue; and hAd, human adipocyte subtype. The adipocyte subtype proportions have been adjusted for covariates and inverse normalized. Values are represented by violin plots. These show the values within the 25th and 75th percentiles, with the median subtype proportion value indicated by the middle line. The whiskers indicate the distance spanning 1.5x the interquartile range and the singular dots showcase the outliers. Source data are provided as a Source Data file.

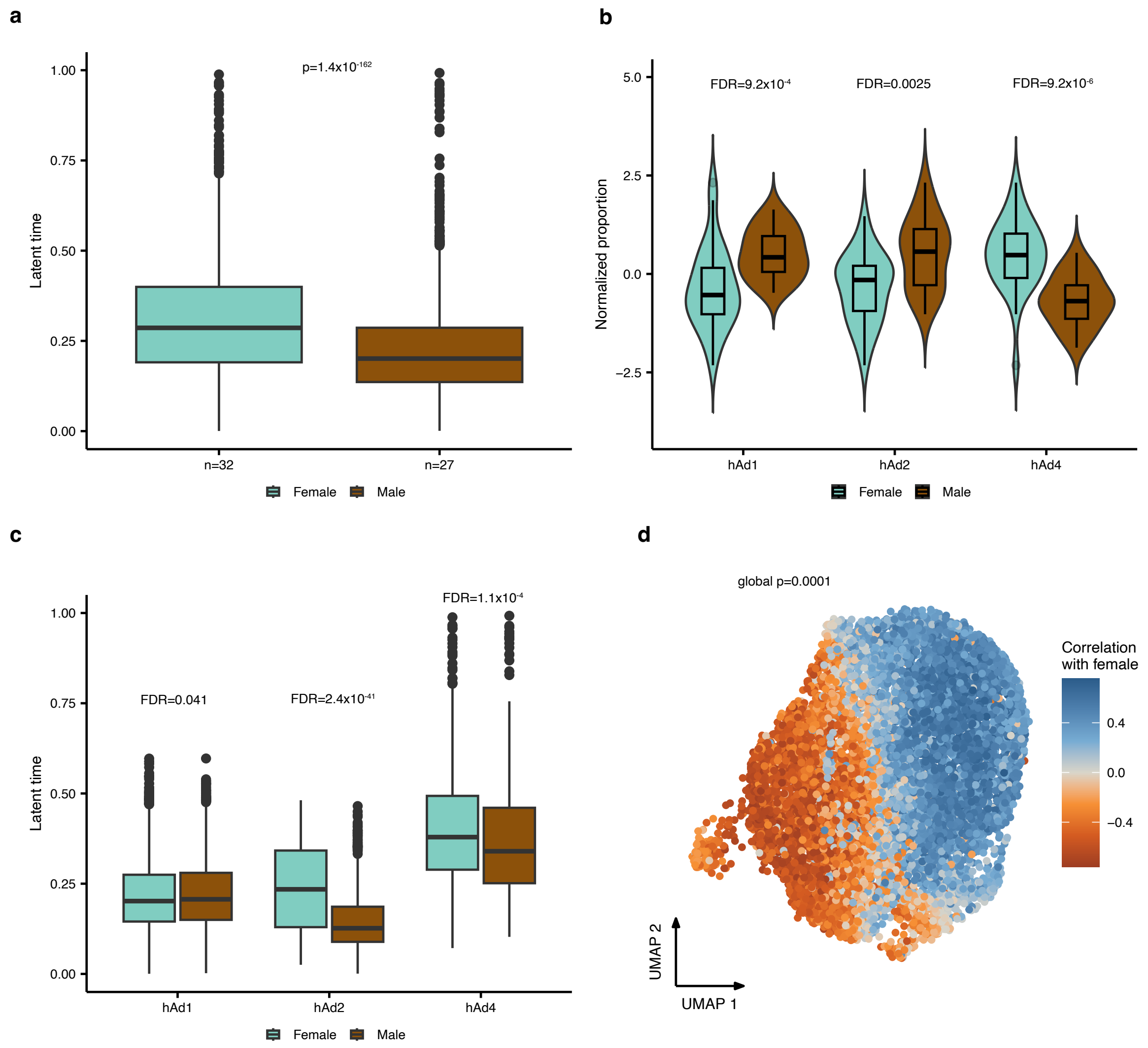

Supplementary Figure 6: SAT subtypes are largely impacted by sex. (a) Boxplots show the lower latent time in males (n=27) when compared to females (n=32) using the two-sided Wilcoxon rank sum test. **(b)** Violin plots showing the inverse normalized proportions (adjusted for age, presence of MASLD and liver fibrosis, batch, BMI, and presence of diabetes) of adipocyte subtypes between sex. Violin plots show multiple SAT adipocyte subtypes as different (FDR<0.05) between males (n=27) and females (n=32) by pairwise comparisons using the two-sided Wilcoxon rank sum test. **(c)** Boxplots showing the latent time difference (FDR<0.05) in adipocytes between males (n=27) and females (n=32) across subtypes by pairwise comparisons using the two-sided Wilcoxon rank sum test. Latent time was first adjusted for biological and technical covariates (age, BMI, presence of fibrosis, MASLD, and diabetes, number of nuclei, and batch). **(d)** UMAP visualization of SAT adipocytes in the KOBS cohort, colored by correlation (neighborhood coefficient) with females (n=32) versus males (n=27) determined by empirical permutation test, and implicating multiple sex-specific adipocyte subtypes. Abbreviations: hAd indicates human adipocyte subtype; FDR, false discovery rate; and BMI, body mass index. Panels **a** and **c** feature the boxplots to represent latent time values. Boxplots show the median latent time values as the middle line, with the values ranging from the 25th to

75th percentiles. The whiskers extend to include 1.5x the interquartile range and the outliers are shown as dots. In panel **b**, the subtype proportions are represented by violin plots. These show the values within the 25th and 75th percentiles, with the median subtype proportion value indicated by the middle line and the whiskers show the distance of 1.5x the interquartile range. The singular dots highlight the outliers. Source data are provided as a Source Data file.

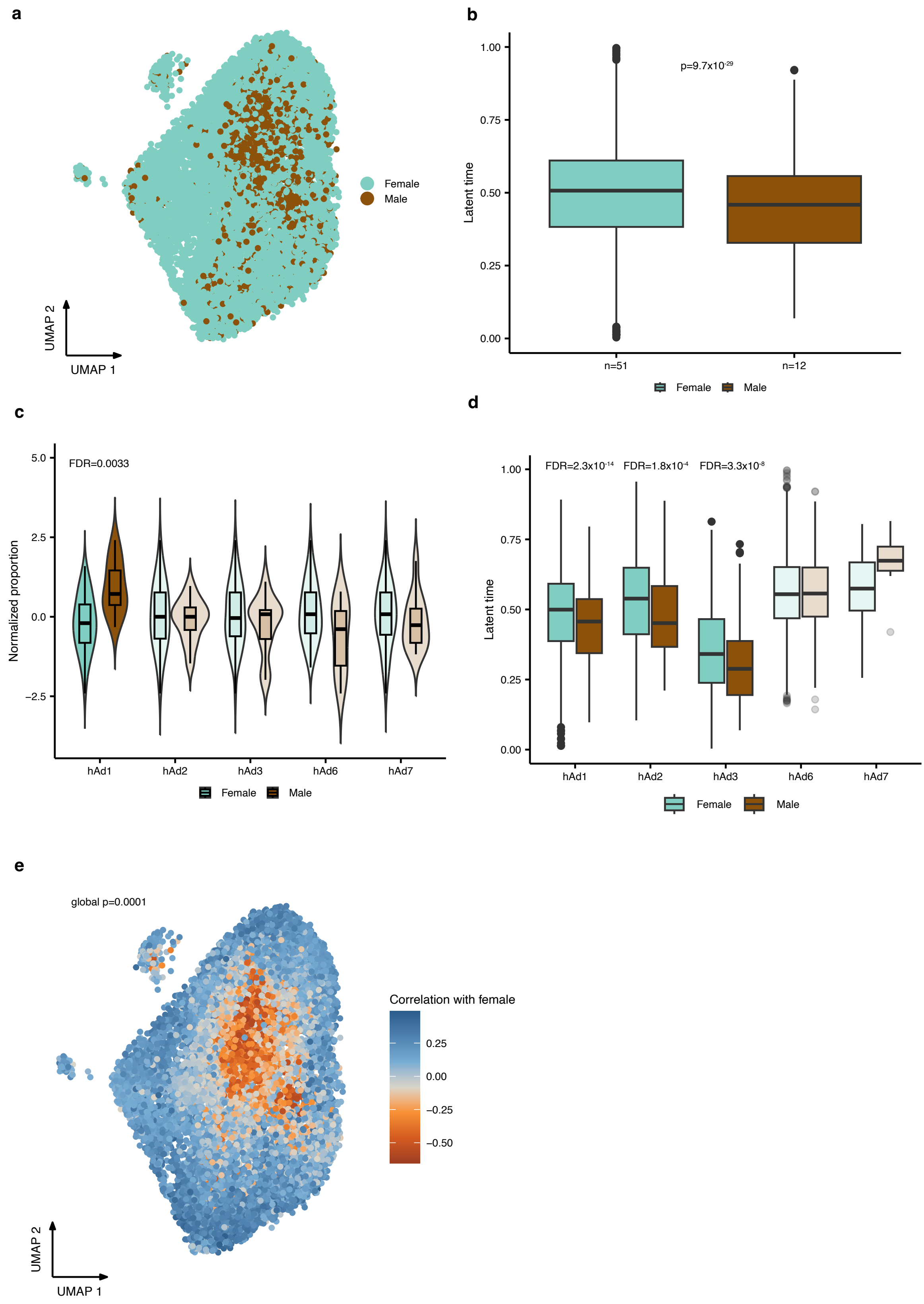

Supplementary Figure 7: Identification of a sex-associated adipocyte subtype in VAT related to MASH. (a) Uniform Manifold Approximation and Projection (UMAP) visualization

of VAT adipocytes in MAFALDA 2 (n=63), colored by sex. **(b)** Boxplots showing the latent time difference in adipocytes between sex using the two-sided Wilcoxon rank sum test. **(c)** Violin plots showing the normalized proportions (adjusted for age, presence of MASLD and liver fibrosis, batch, body mass index (BMI), and presence of diabetes) of adipocyte subtypes between sex. Adipocyte subtype, hAd1, also MASH-associated, identified as different (FDR <0.05) between males (n=12) and females (n=51). **(d)** Boxplots showing the latent time differences between sex and adipocyte subtypes, determined by pairwise comparisons using the two-sided Wilcoxon rank sum test. Boxplots show the (FDR<0.05) lower latent time in males (n=12) versus females (n=51) in multiple VAT adipocyte subtypes. **(e)** UMAP visualization of VAT adipocytes, colored by correlation (neighborhood coefficient) with females (n=51) versus males (n=12) with significance determined via empirical permutation test, illustrating the sex-specific subtype, hAd1. Abbreviations: VAT indicates visceral adipose tissue; hAd indicates human adipocyte subtype; MASLD, metabolic dysfunction-associated steatotic liver disease; MASH, metabolic dysfunction-associated steatohepatitis; and FDR, false discovery rate. Panels **b** and **d** use boxplots to represent the latent time values that have been adjusted for age, presence of MASLD and liver fibrosis, batch, BMI, and presence of diabetes. In the boxplots, the 25th and 75th percentile values are indicated by the box with the middle lines representing the median latent time values. Whiskers are used to reveal the distance of 1.5x the interquartile range, and the outliers are shown using the individual dots. Panel **c** shows the violin plots that showcase the adjusted and inverse normalized subtype proportions. The upper and lower bounds show the 25th and 75th percentiles, with whiskers indicating the distance spanning 1.5x the interquartile range (minimum and maximum). Median value in the subtype proportions is indicated by the middle line and outliers are the dots. Source data are provided as a Source Data file.

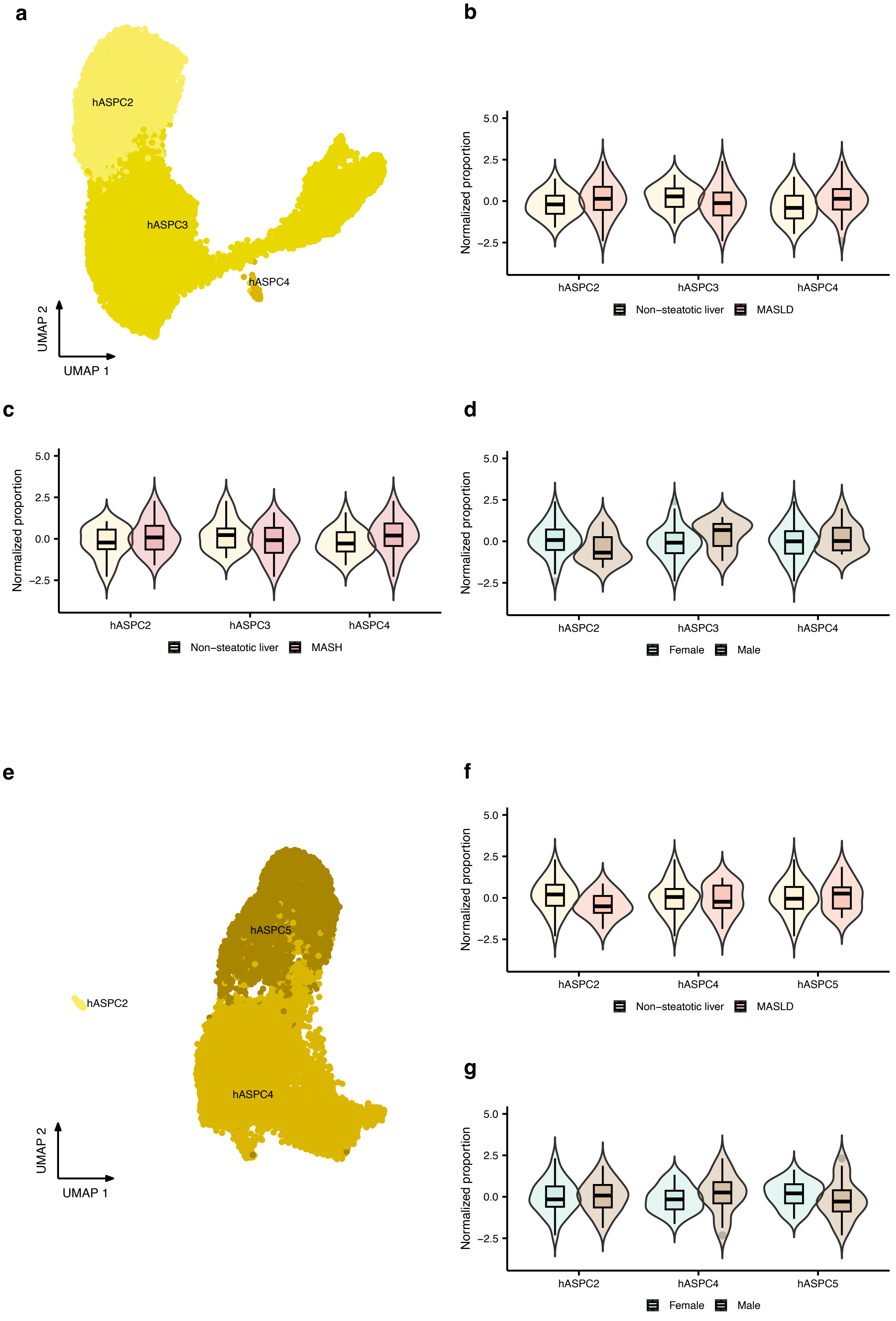

Supplementary Figure 8: Adipose stem and progenitor cells (ASPCs) subtypes in

MAFALDA 2 and KOBS. (a) Uniform Manifold Approximation and Projection (UMAP) visualization of MAFALDA 2 ASPCs, colored by the ASPC subtypes. **(b)** Violin plots of ASPC subtypes showcasing that no ASPC subtype proportions differ by the MASLD status in MAFALDA 2. **(c)** Violin plots reveal that no ASPC subtype proportions differ between the individuals with MASH and the individuals with non-steatotic livers in MAFALDA 2. **(d)** Violin plots show no significant difference in ASPC subtype proportions by sex in MAFALDA 2. **(e)** UMAP of KOBS ASPCs, colored by the ASPC subtypes. **(f)** Violin plots show that no ASPC subtype proportions differ by the MASLD status. **(g)** Violin plots of ASPC subtype proportions show that they do not differ by sex in KOBS. In panels b-d and f-g all pairwise comparisons were done using the two-sided Wilcoxon rank sum test and adjusted for multiple testing using false discovery rate (FDR) (FDR<0.05). Abbreviations: hASPC indicates human adipose stem and progenitor cell subtype; GO, gene ontology. Panels **b-d**, and **f-g** are the violin plots that showcase the adjusted and inverse normalized ASPC subtype proportions The upper and lower bounds show the 25th and 75th percentiles, with whiskers spanning 1.5x the interquartile range. Median value in the subtype proportions is indicated by the middle line, and outliers are the singular dots. Source data are provided as a Source Data file.

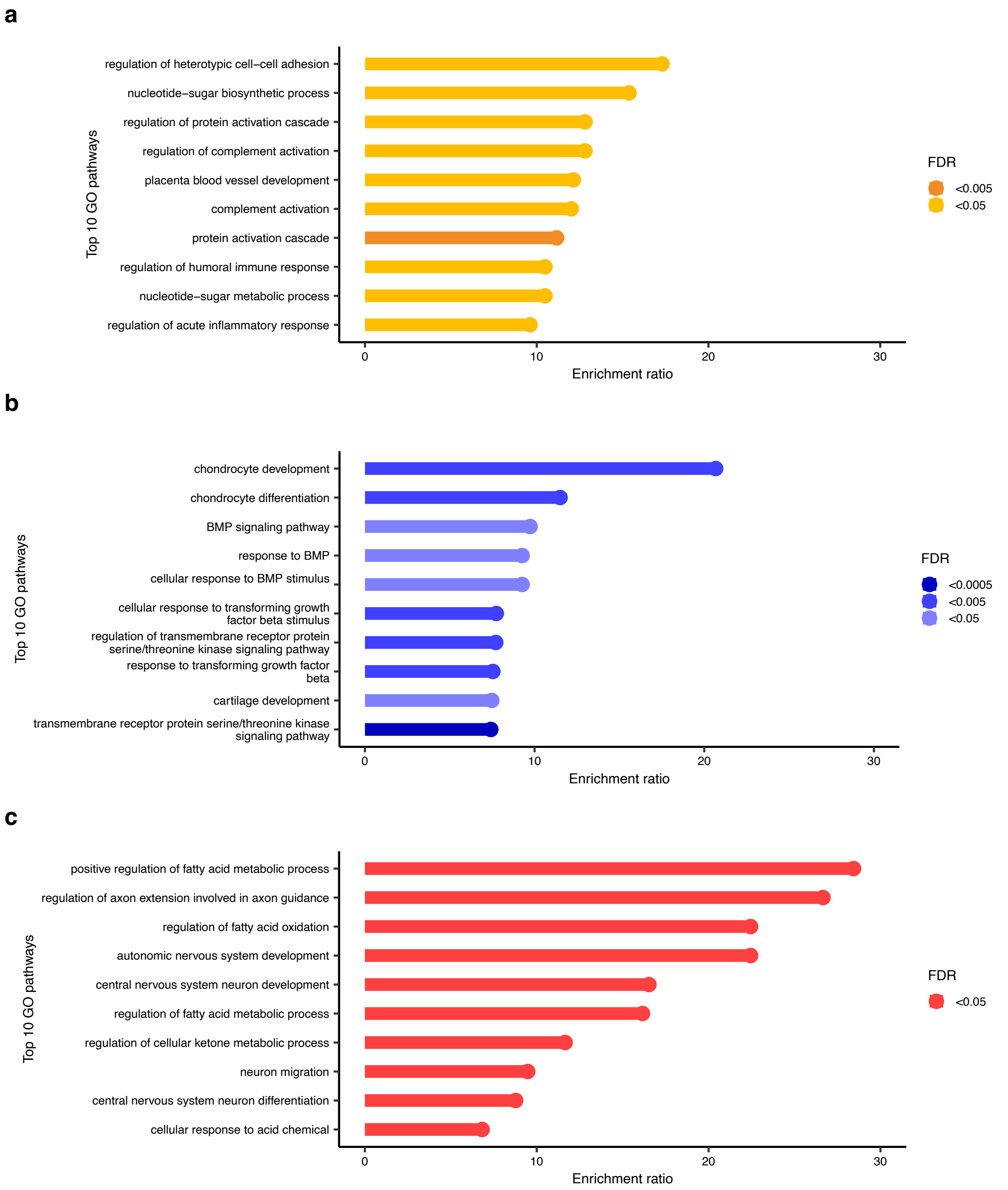

Supplementary Figure 9: Pathway enrichments of the differentially expressed genes by MASLD in VAT and SAT adipose stem and progenitor cells (ASPCs) in MAFALDA 2 and KOBS. (a) Horizontal lollipop plots show the top 10 (FDR<0.05) GO functionally enriched pathways for the VAT ASPC genes upregulated in the individuals with MASLD in MAFALDA 2 (log_2FC>0.25 and Bonferroni adjusted p<0.05). The upregulated genes by MASLD are enriched for pathways of nucleotide-sugar biosynthetic process, and immune and inflammatory responses. **(b)** Horizontal lollipop plots feature the top 10 (FDR<0.05) GO functionally enriched pathways for the SAT ASPC genes upregulated in the individuals with MASLD in KOBS (log_2FC>0.25 and Bonferroni adjusted p<0.05). The upregulated genes by MASLD in SAT ASPCs are enriched

for growth factor response pathways. **(c)** Horizontal lollipop plots highlight the top 10 (FDR<0.05) GO functionally enriched pathways for the SAT ASPC genes downregulated in the individuals with MASLD in KOBS ($log_2FC<-0.25$ and Bonferroni adjusted $p<0.05$). The downregulated genes by MASLD in SAT ASPCs are enriched for fatty acid processes. Abbreviations: VAT indicates visceral adipose tissue; SAT, subcutaneous adipose tissue; MASLD, metabolic dysfunction-associated steatotic liver disease; FDR indicates false discovery rate; and GO, gene ontology. Source data are provided as a Source Data file.

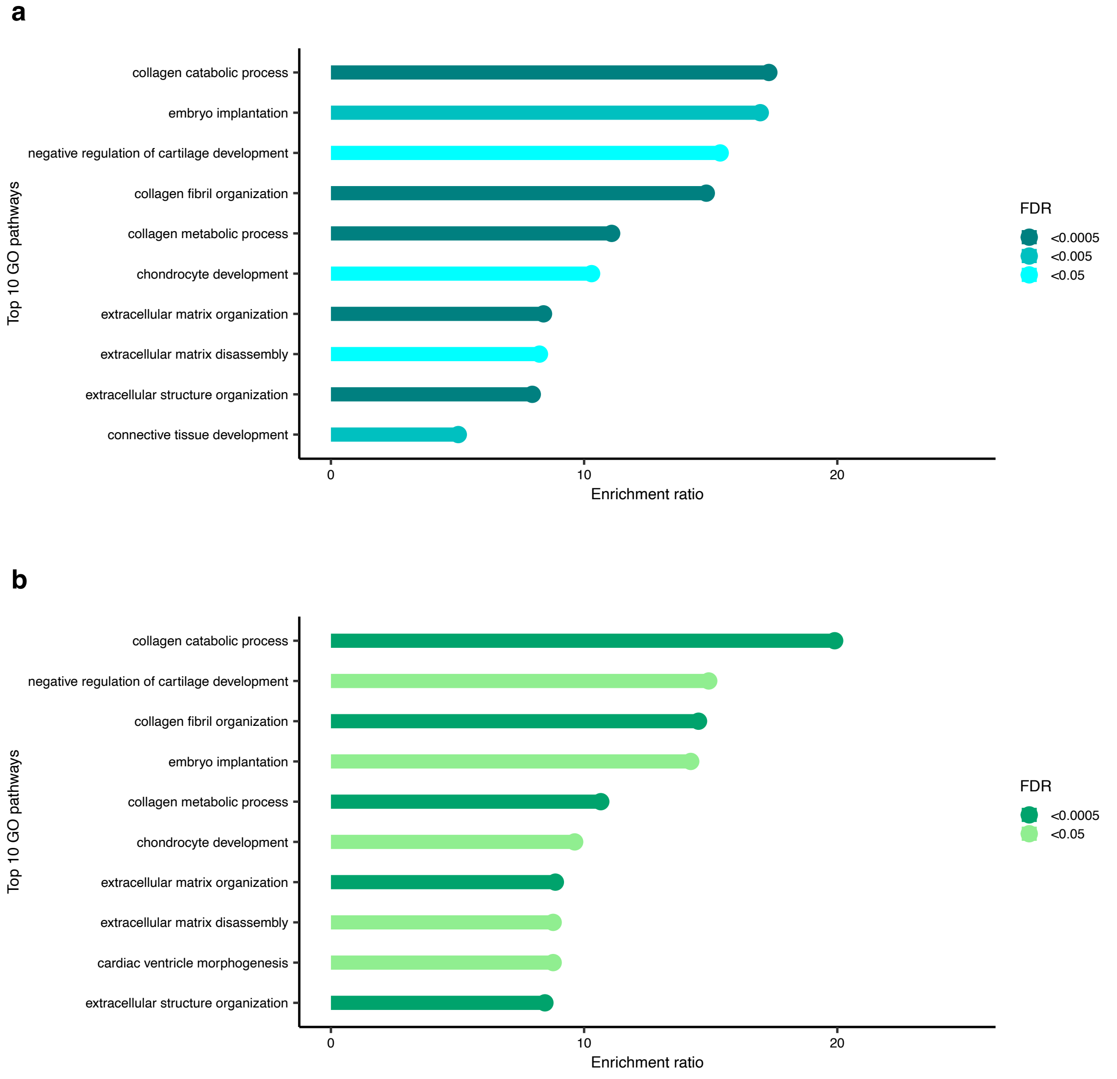

Supplementary Figure 10: Pathway enrichments of differentially expressed genes upregulated in females in VAT and SAT adipose stem and progenitor cells (ASPCs) in MAFALDA 2 and KOBS. (a) Horizontal lollipop plots show the top 10 (FDR<0.05) GO functionally enriched pathways for the ASPC genes upregulated in females in MAFALDA 2 (log_2FC>0.25 and Bonferroni adjusted p<0.05). The upregulated genes in females in VAT ASPCs feature pathway enrichments for collagen and extracellular matrix organization. **(b)** Horizontal lollipop plots reveal the top 10 (FDR<0.05) GO functionally enriched pathways for SAT ASPC genes upregulated in females in KOBS (log_2FC>0.25 and Bonferroni adjusted p<0.05). The upregulated genes in females in SAT ASPCs share similar pathway enrichments to VAT ASPCs genes upregulated in females, including collagen processes and extracellular matrix organization. Abbreviations: VAT indicates visceral adipose tissue; SAT, subcutaneous adipose tissue; FDR, false discovery rate; and GO, gene ontology. Source data are provided as a Source Data file.

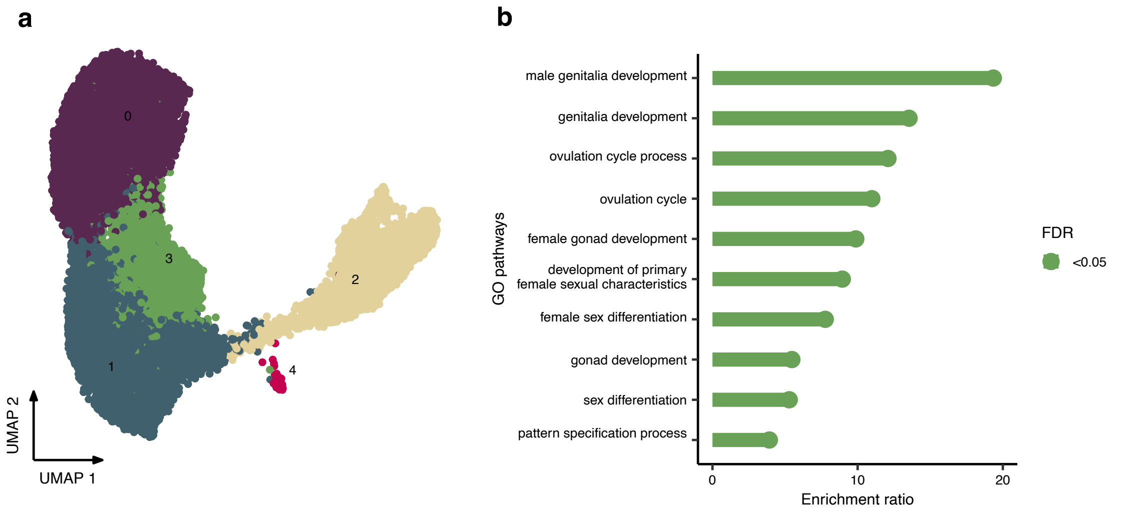

Supplementary Figure 11: Louvain clustering and enrichment in adipose stem and progenitor cells (ASPCs) in MAFALDA 2. (a) Uniform Manifold Approximation and Projection (UMAP) visualization of MAFALDA 2 ASPCs, colored by Louvain clustering. **(b)** Horizontal lollipop plots highlight the GO functionally enriched pathways of the marker genes of Louvain cluster 3. The genes of Louvain cluster 3 are enriched for sex-specific pathways, including ovulation cycle and sex differentiation. Abbreviations: FDR indicates false discovery rate; and GO, gene ontology. Source data are provided as a Source Data file.

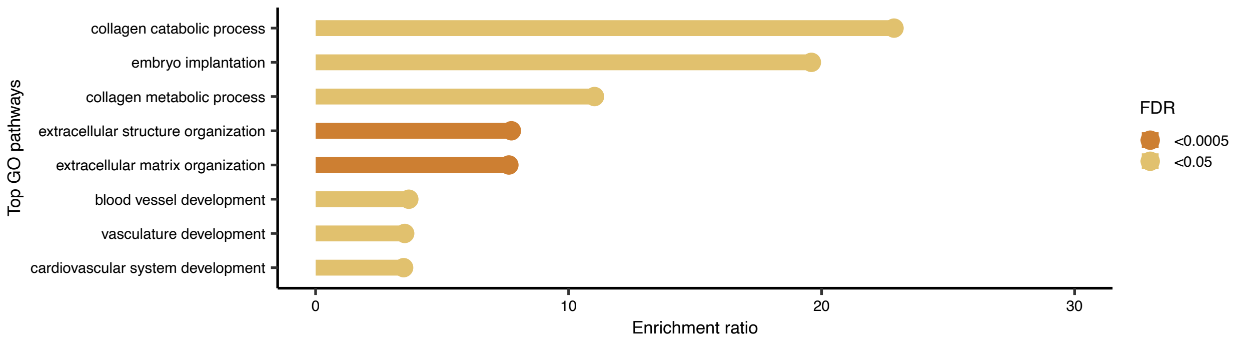

Supplementary Figure 12: Pathway enrichment of the 90 marker genes differentially expressed (DE) by sex in the adipose stem and progenitor cell (ASPC) subtype, hASPC5, in KOBS. Horizontal lollipop plots of the top (FDR<0.05) GO functionally enriched pathways among the 90 marker genes DE by sex in hASPC5 in KOBS. These pathways include collagen processes and extracellular matrix and structure organization. Abbreviations: hASPC indicates human adipose stem and progenitor cell subtype; FDR, false discovery rate; and GO, gene ontology. Source data are provided as a Source Data file.

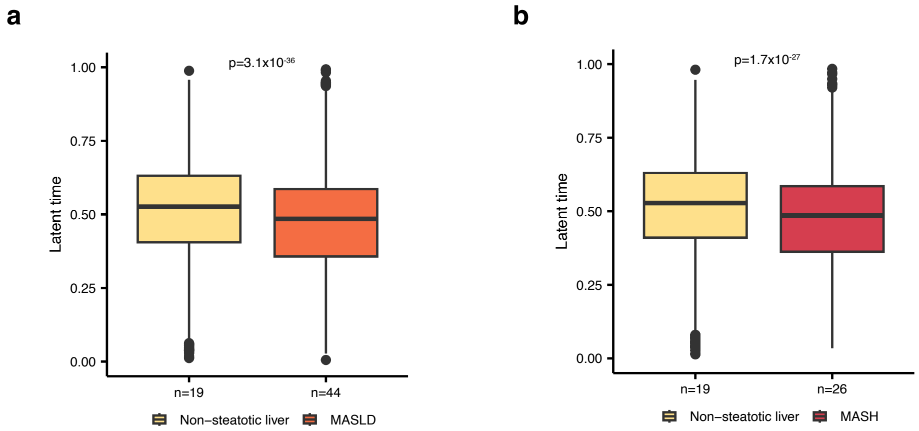

Supplementary Figure 13: Comparison of the latent time in VAT adipocytes from MAFALDA 2 between the individuals with and without MASLD/MASH when adjusting for the *PNPLA3* genotype status of rs738409[9]. (a) Boxplots display a lower latent time in the individuals with MASLD (either steatosis or MASH) (n=44) when compared to the individuals with non-steatotic livers (n=19) with adjustment for the following covariates: batch, number of adipocytes per sample, age, sex, body mass index (BMI), diabetic status, fibrotic status, and the *PNPLA3* genotype status of rs738409[9], using the two-sided Wilcoxon rank sum test. **(b)** Boxplots showcase a lower latent time in the individuals with MASH (n=26) when compared to the individuals with non-steatotic livers (n=19) with adjustment for batch, number of adipocytes per sample, age, sex, BMI, diabetic status, fibrotic status, and the *PNPLA3* genotype status of rs738409[9], using the two-sided Wilcoxon rank sum test. Abbreviations: VAT indicates visceral adipose tissue; MASLD, metabolic dysfunction-associated steatotic liver disease; and MASH, metabolic dysfunction-associated steatohepatitis. In boxplots of panels **a** and **b**, the boundary points show the 25th and 75th percentile, the middle lines the median latent time values, and the whiskers the distance spanning 1.5x the interquartile range. Outliers are represented by the single dots. Source data are provided as a Source Data file.

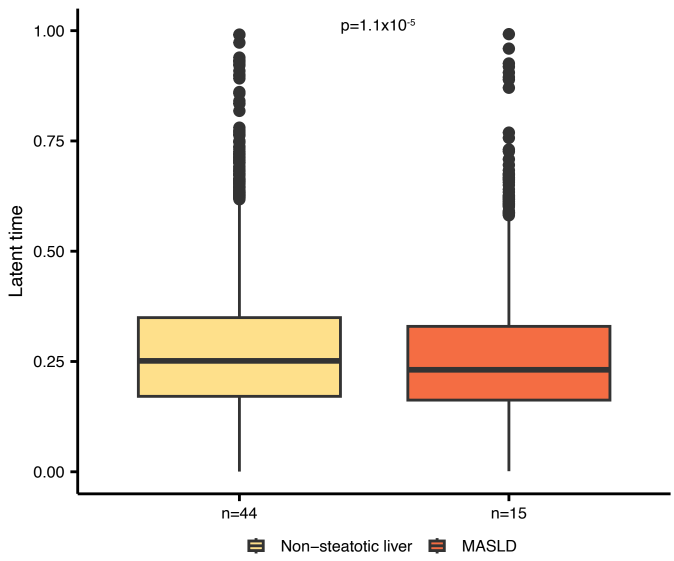

Supplementary Figure 14: Comparison of the latent time in SAT adipocytes adjusted for the *PNPLA3* genotype status of rs738409[9] in KOBS. Boxplot shows a lower latent time in the individuals with MASLD (n=15) than in the individuals with non-steatotic livers (n=44). Latent time was adjusted for batch, number of adipocytes per sample, age, sex, body mass index, diabetic status, fibrotic status, and the *PNPLA3* genotype status of rs738409[9]. Significance was obtained using the two-sided Wilcoxon rank sum test. The boxplot shows the 25th and 75th percentiles, the middle lines as the median latent time values, and the whiskers span the distance of 1.5x the interquartile range. Outliers are represented by the single dots. Source data are provided as a Source Data file.

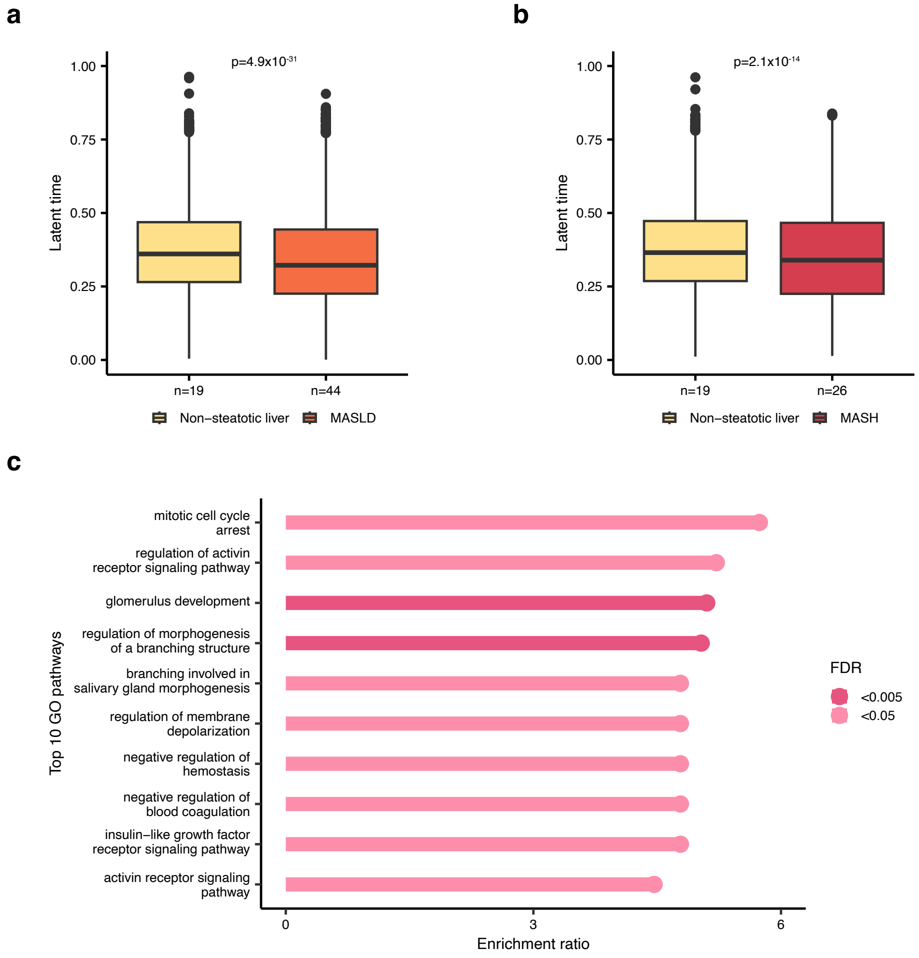

Supplementary Figure 15: Comparison of the latent time in VAT adipocytes from MAFALDA 2 between the individuals with and without MASLD/MASH using the subset of the genes applied to predict the VAT adipocyte differentiation degree (n=666 genes). (a) Boxplots reveal a lower latent time in the individuals with MASLD (either steatosis or MASH) (n=44) than in the individuals with non-steatotic livers (n=19) using the two-sided Wilcoxon rank sum test. The latent time was obtained using the overlapping genes between the predicted degree of VAT adipocyte differentiation gene set (i.e. entire latent time gene set) and the bulk RNA-sequencing differentially expressed genes (DEGs) between adipose stem and progenitor cells (ASPCs) and differentiated adipocytes, referred to as the subset of predicted degree of adipocyte differentiation gene set (n=666 genes) (see Methods). **(b)** Boxplots show a lower latent time in the individuals with MASH (n=26) than in the individuals with non-steatotic livers (n=19) using the two-sided Wilcoxon rank sum test. The latent time was obtained as in panel **a** (n=666 genes). **(c)** Horizontal lollipop plot of the top 10 GO functionally enriched pathways (FDR<0.05) among the subset gene set (n=666 genes). Abbreviations: VAT indicates visceral adipose tissue; MASLD, metabolic dysfunction-associated steatotic liver disease; MASH, metabolic dysfunction-associated steatohepatitis; FDR indicates false discovery rate; and GO, gene ontology. In panels **a** and **b**, the latent time values have been adjusted for age, sex, batch,

number of nuclei, presence of diabetes, and presence of liver fibrosis. Data are represented as a boxplot with whisker end points indicating 1.5x the interquartile range, upper and lower bounds showing the 25th and 75th percentiles, and the middle lines depicting the median values. Outliers are indicated by singular dots. Source data are provided as a Source Data file.

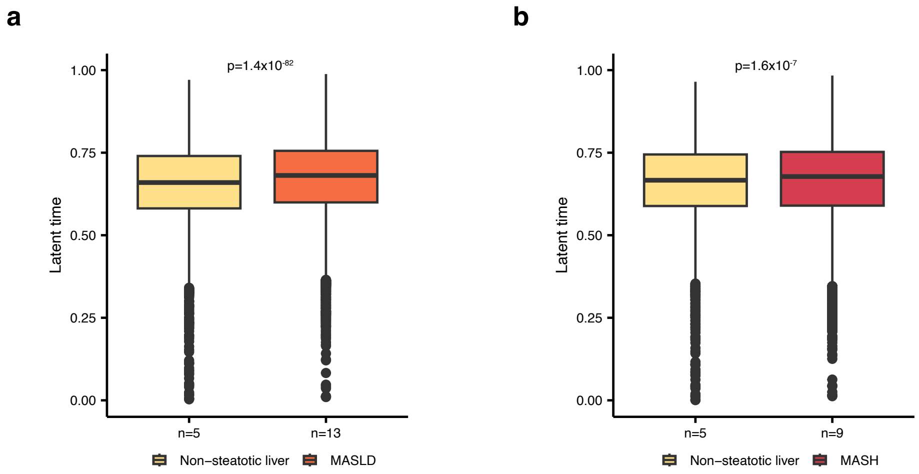

Supplementary Figure 16: Latent time analysis in liver hepatocytes using liver snRNA-seq data[11] reveals a higher latent time in individuals with MASLD/MASH in comparison to individuals with non-steatotic livers. (a) Boxplots show a higher latent time in the individuals with MASLD (either steatosis or MASH) (n=13) when compared to the individuals with non-steatotic livers (n=5). Latent time was adjusted for the number of hepatocytes per sample, age, sex, body mass index (BMI), and fibrotic status and assessed for significance using the two-sided Wilcoxon rank sum test. **(b)** Boxplots highlight a higher latent time in the individuals with MASH (n=9) when compared to the individuals with non-steatotic livers (n=5) with adjustment for the number of hepatocytes per sample, age, sex, BMI, and fibrotic status and using the two-sided Wilcoxon rank sum test. Abbreviations: MASLD indicates metabolic dysfunction-associated steatotic liver disease; and MASH, metabolic dysfunction-associated steatohepatitis. In boxplots of panels **a** and **b**, the boundary points are the 25th and 75th percentiles, while the middle lines are the median latent time values. The whiskers indicate the distance spanning 1.5x the interquartile range and outliers are shown by the single dots. Source data are provided as a Source Data file.

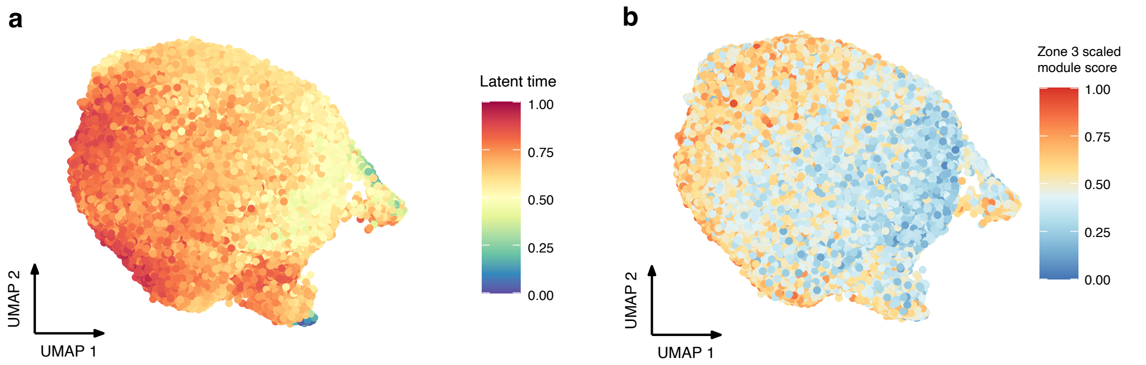

Supplementary Figure 17: Hepatocyte zone 3 coincides with a higher latent time in liver snRNA-seq data[11]. (a) Uniform Manifold Approximation and Projection (UMAP) visualization of hepatocytes, colored by latent time. **(b)** UMAP of hepatocytes colored by module scores from zone 3 marker genes (n=1078 marker genes). The scale ranges from 0 to 1, with blue implicating little to no expression and red showing high expression of the liver zone 3 marker gene set. Source data are provided as a Source Data file.

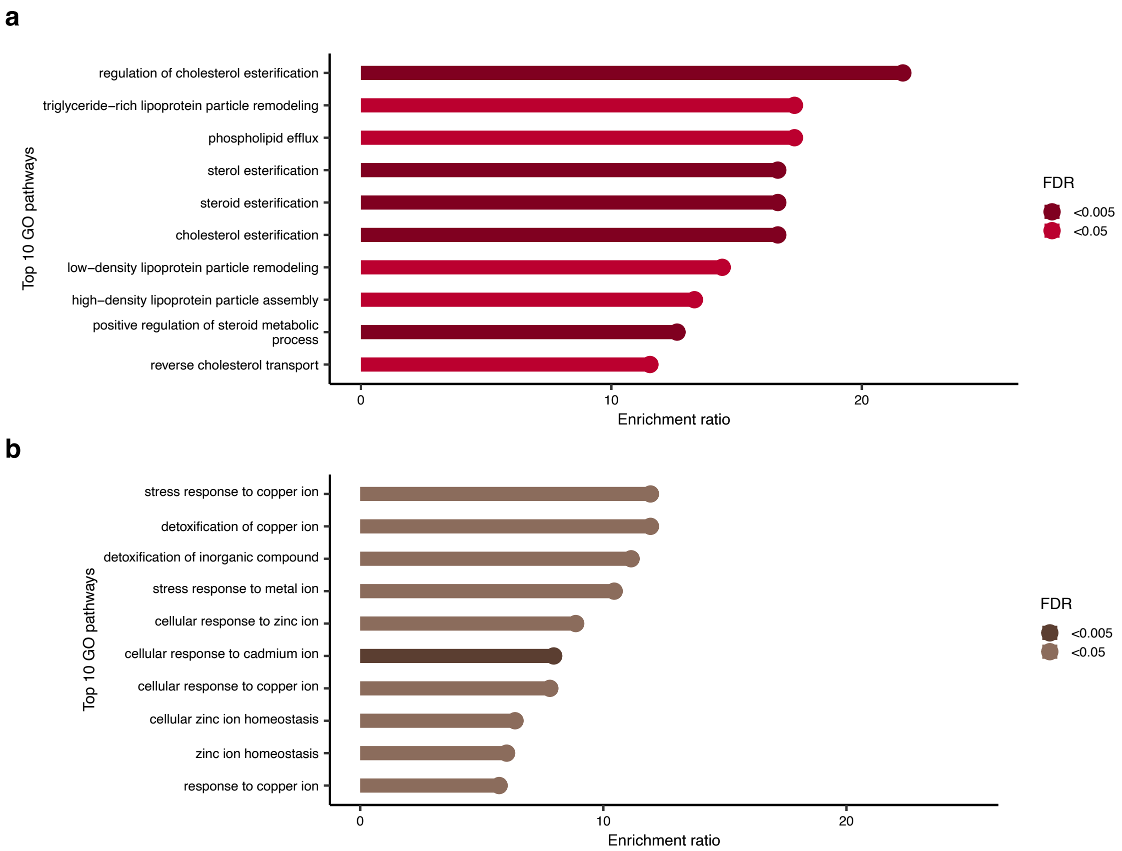

Supplementary Figure 18: Pathway enrichments of the differentially expressed genes by MASLD among the genes used to predict the degree of hepatocyte differentiation in liver snRNA-seq data[11]. (a) Horizontal lollipop plots show the top 10 (FDR<0.05) GO functionally enriched pathways for the upregulated genes by MASLD among the genes used to predict the degree of hepatocyte differentiation ($\log_2$FC>0.25 and Bonferroni adjusted p<0.05). These genes are enriched for pathways of cholesterol and sterol esterification. **(b)** Horizontal lollipop plots feature the top 10 (FDR<0.05) GO functionally enriched pathways for the downregulated genes by MASLD among the genes used to predict the degree of hepatocyte differentiation ($\log_2$FC>0.25 and Bonferroni adjusted p<0.05). These genes are enriched for regulation of copper and zinc pathways. Abbreviations: FDR indicates false discovery rate; GO, gene ontology; and MASLD, metabolic dysfunction-associated steatotic liver disease. Source data are provided as a Source Data file.

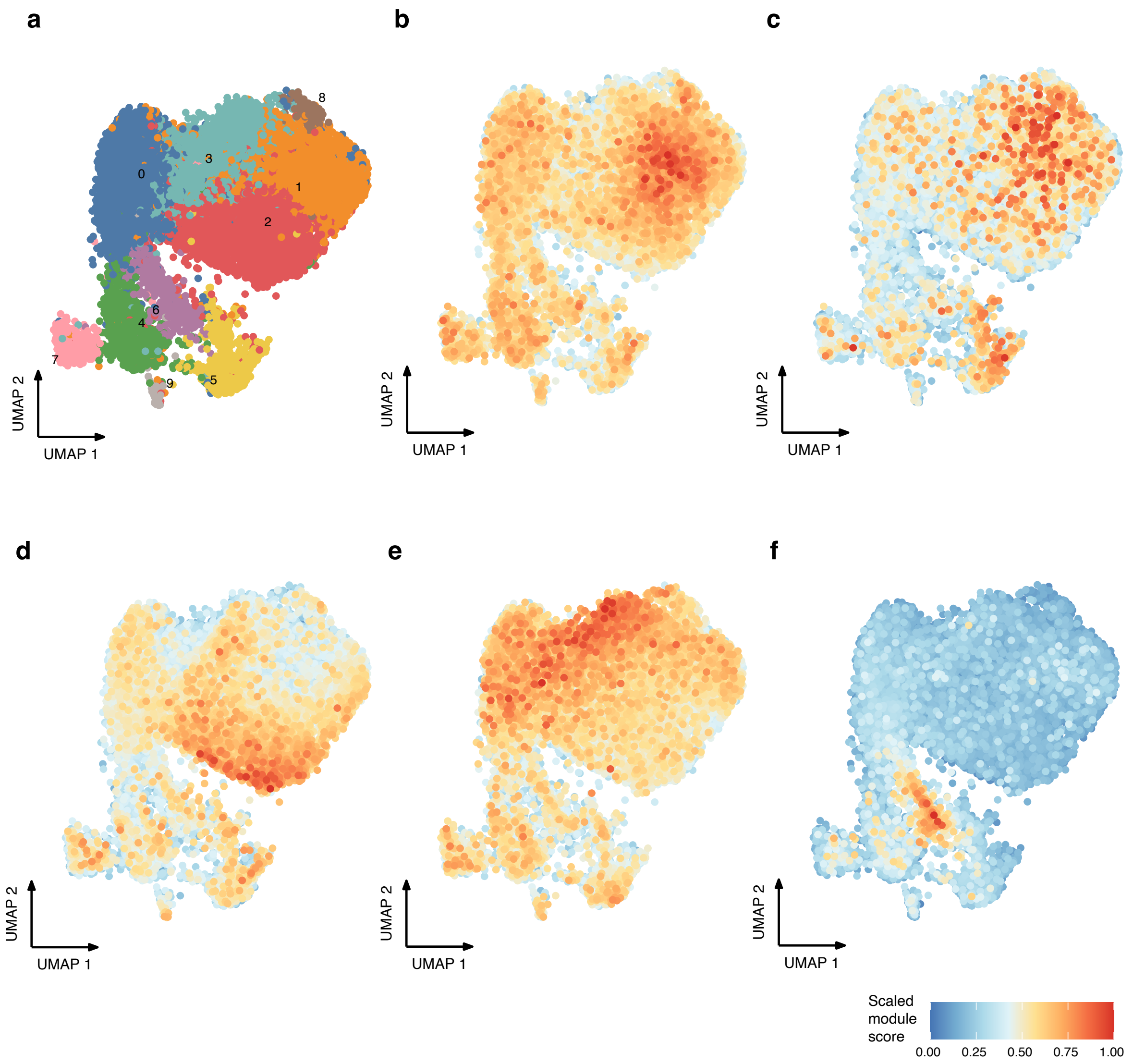

Supplementary Figure 19: Module scores of VAT adipocyte subtype marker genes from MAFALDA 2 displayed in VAT adipocytes from MAFALDA 1. (a) Uniform Manifold Approximation and Projection (UMAP) of adipocytes, colored by Louvain cluster (n=9 Louvain clusters). **(b)** UMAP visualization of adipocytes in MAFALDA 1, colored by module scores from MAFALDA 2 hAd1 subtype marker genes (n=252 marker genes). **(c)** UMAP of adipocytes in MAFALDA 1, colored by module scores from MAFALDA 2 hAd2 subtype marker genes (n=246 marker genes). **(d)** UMAP of adipocytes in MAFALDA 1, colored by module scores from MAFALDA 2 hAd3 subtype marker genes (n=506 marker genes). **(e)** UMAP of adipocytes in MAFALDA 1, colored by module scores from MAFALDA 2 hAd6 subtype marker genes (n=141 marker genes). **(f)** UMAP of adipocytes in MAFALDA 1, colored by module scores from MAFALDA 2 hAd7 subtype marker genes (n=195 marker genes). Abbreviations: VAT indicates visceral adipose tissue; and hAd, human adipocyte subtype. Panels **b**-**f** all use the same scale, shown to the bottom of panel **f**. The scale ranges from 0 to 1, with blue signifying little to no

expression and red indicating high expression of the respective marker gene set. Source data are provided as a Source Data file.

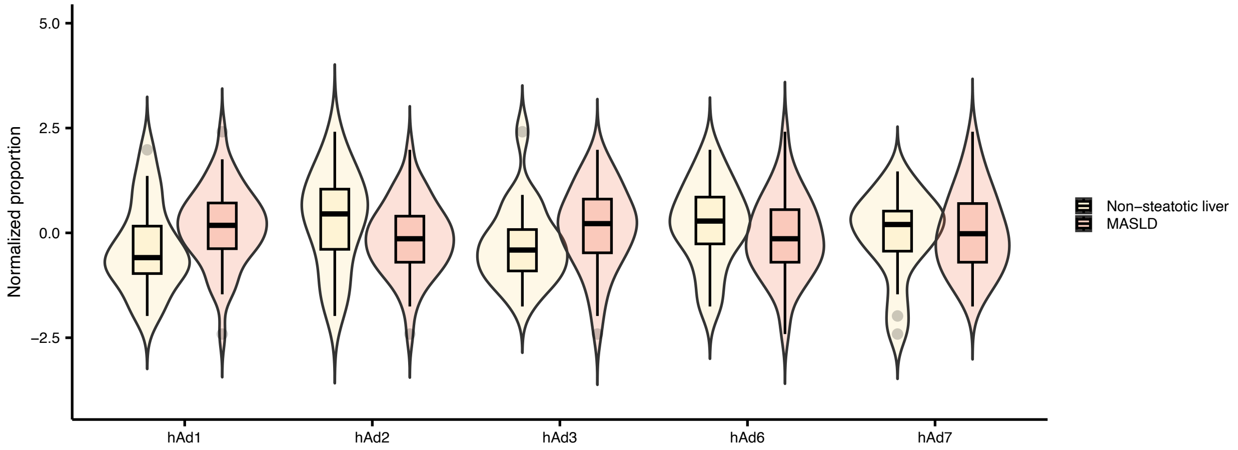

Supplementary Figure 20: No significant differences were observed in the VAT adipocyte subtype proportions between the individuals with MASLD and without MASLD. Boxplots show that the normalized proportions of VAT adipocyte subtypes do not differ between the individuals with (n=44) and without MASLD (n=19) in MAFALDA 2 by pairwise comparisons using the two-sided Wilcoxon rank sum test and adjusting for multiple testing with false discovery rate (FDR) (FDR<0.05). Abbreviations: VAT indicates visceral adipose tissue; hAd, human adipocyte subtype; and MASLD, metabolic dysfunction-associated steatotic liver disease. The adipocyte subtype proportions are represented via violin plots. Values have been adjusted for covariates and inverse normalized. These highlight the values within the 25th and 75th percentiles, with the median subtype proportion value shown by the middle line. Whiskers highlight the distance of 1.5x the interquartile range with the singular dots indicating outliers. Source data are provided as a Source Data file.

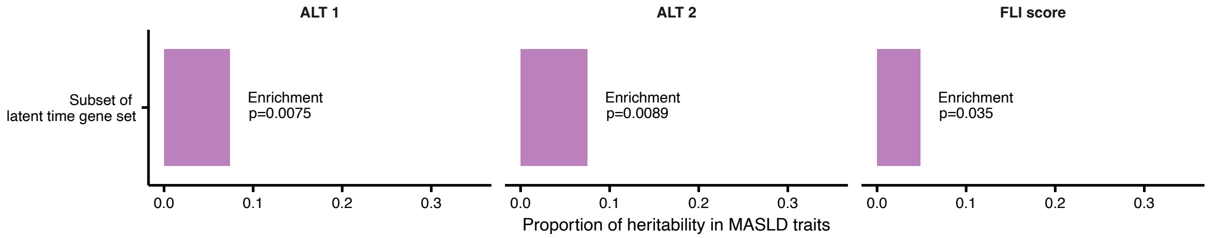

Supplementary Figure 21: The subset of the VAT genes used to predict the degree of adipocyte differentiation explains a large proportion of heritability in MASLD traits in the UK Biobank. Horizontal barplots showing the proportions of heritability explained by the variants in the *cis* regions of the genes of a subset of the predicted degree of adipocyte differentiation gene set (i.e. latent time gene set) (n=666 genes). This subset shows enrichment for heritability of ALT 1 (proportion h^2=7.40%, p=0.0075), ALT 2 (proportion h^2=7.50%, p=0.0089), and the fatty liver index (FLI) (proportion h^2=4.85%, p=0.035). ALT 1 refers to ALT adjusted for alcohol consumption while ALT 2 does not include individuals with heavy alcohol consumption. Abbreviations: VAT indicates visceral adipose tissue; and MASLD, metabolic dysfunction-associated steatotic liver disease. Source data are provided as a Source Data file.

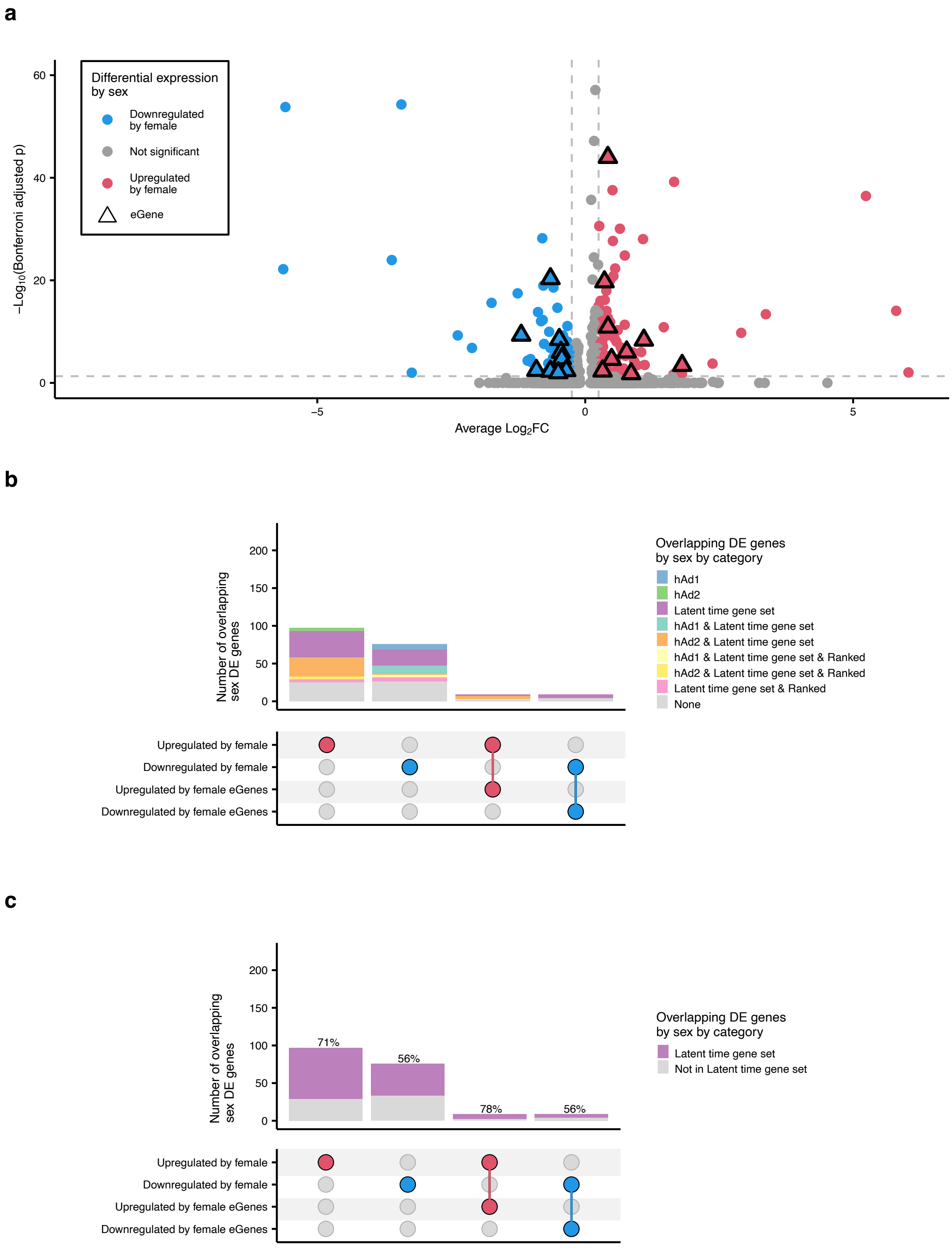

Supplementary Figure 22: A total of 191 VAT adipocyte genes are differentially expressed (DE) by sex in VAT adipocytes and show strong overlaps with the genes used to predict the degree of adipocyte differentiation. (a) Volcano plot showcases the significantly differentially

expressed (DE) genes by sex in VAT adipocytes (n=191 genes) (Bonferroni adjusted p<0.05 and absolute $log_2FC>0.25$ in MAFALDA 2 (n=63 individuals). Covariates included in the model were age, batch, BMI, number of nuclei, presence of diabetes, presence of MASLD, and presence of liver fibrosis. The DE genes by sex in VAT adipocytes that were also identified as significant (FDR<0.05) adipocyte *cis*-eQTL target genes (i.e., adipocyte eGenes) from Matrix eQTL are labelled. Red dots indicate significantly upregulated genes in females, while blue dots indicate significantly downregulated genes in females. **(b)** Upset plot features the intersection of the genes DE by sex in VAT adipocytes of the following four categories: Upregulated genes by females, downregulated genes by females, upregulated genes by females that are also adipocyte eGenes, and downregulated genes by females that are also adipocyte eGenes. Colors of the bars in upset plot indicate the number of genes present in each gene set of interest (Predicted degree of adipocyte differentiation (i.e., latent time), Ranked, hAd1, hAd2). Abbreviations: VAT indicates visceral adipose tissue; MASLD, metabolic dysfunction-associated steatotic liver disease; eGene, *cis*-eQTL target genes; and hAd indicates human adipocyte subtype. **(c)** Upset plot of sex DE genes, featuring the intersection of genes DE by sex of the following four categories: upregulated genes by females, downregulated genes by females, upregulated genes by females that are also adipocyte eGenes, and downregulated genes by females that are also adipocyte eGenes. Colors represent the genes in the latent time gene set and genes not in the gene set. DE analysis in panel **a** was done using the hurdle model approach in 'FindMarkers' from Seurat[12] (see Methods) and significance determined by Bonferroni adjusted p-value<0.05 and absolute log2FC>0.25. Source data are provided as a Source Data file.

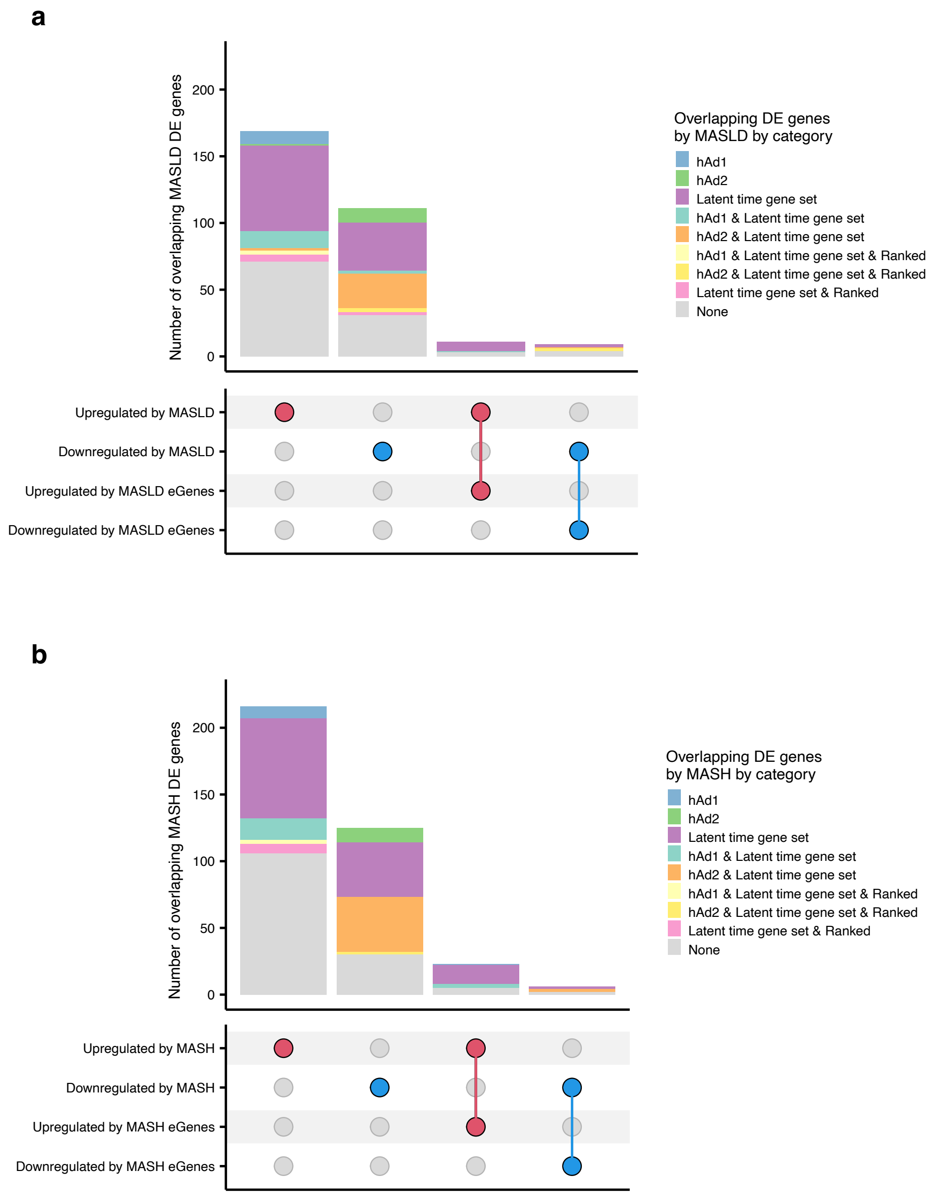

Supplementary Figure 23: A total of 300 and 370 genes are differentially expressed (DE) by MASLD and MASH in VAT adipocytes and show strong overlaps across the genes used to predict the degree of adipocyte differentiation. (a) Upset plot of MASLD DE genes in VAT adipocytes from MAFALDA 2 (n=63 individuals), featuring the intersection of genes DE by MASLD of the following four categories: upregulated genes by MASLD, downregulated genes by MASLD, upregulated genes by MASLD that are also adipocyte eGenes, and downregulated genes by MASLD that are also adipocyte eGenes. Colors of bars in upset plot indicate the number of genes present in each gene set of interest (Predicted degree of adipocyte differentiation (i.e., latent time), Ranked, hAd1, and hAd2). **(b)** Upset plot of MASH DE genes in VAT adipocytes from MAFALDA 2 (n=63 individuals), featuring the intersection of genes DE by MASH of the following four categories: upregulated genes by MASH, downregulated genes by MASH, upregulated genes by MASH that are also adipocyte eGenes, and downregulated genes by MASH that are also adipocyte eGenes. Colors of bars in upset plot indicate the number

of genes present in each gene set of interest (Latent time, Ranked, hAd1, and hAd2). Abbreviations: VAT indicates visceral adipose tissue; MASLD, metabolic dysfunction-associated steatotic liver disease; hAd, human adipose subtype; eGene, *cis*-eQTL target gene. and MASH, metabolic dysfunction-associated steatohepatitis. Source data are provided as a Source Data file.

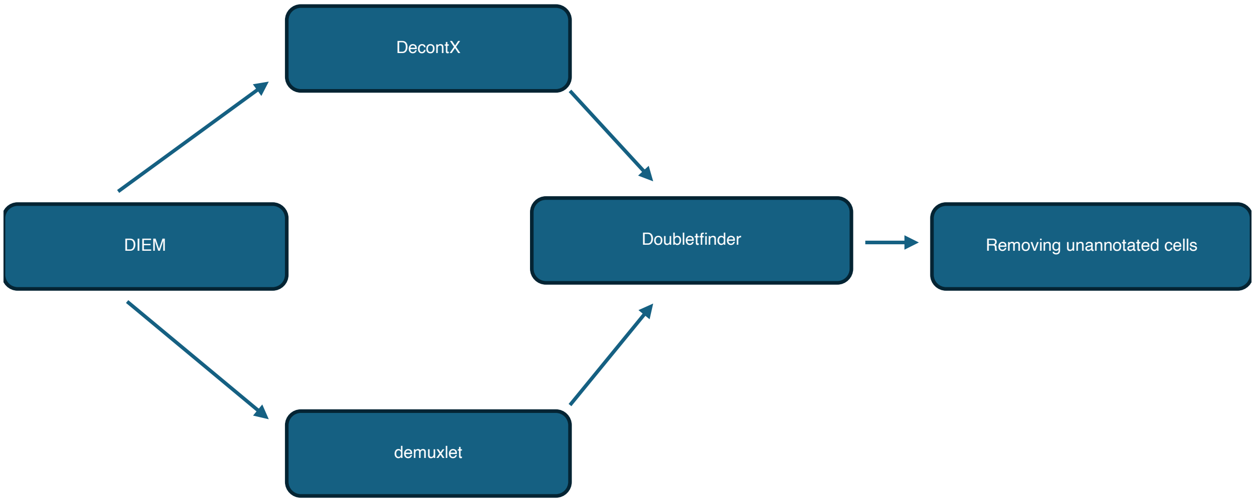

Supplementary Figure 24: Quality control pipeline performed for snRNA-seq data. Flow chart of the quality control pipeline performed to obtain high quality nuclei in MAFALDA 1, MAFALDA 2, and KOBS data. As samples in MAFALDA 1 were not multiplexed, they were not processed with demuxlet[13]. Demuxlet[13] and DecontX[14] were run in parallel. For more details, see Methods.

Supplementary References

1. Emont, M. P. *et al.* A single-cell atlas of human and mouse white adipose tissue. *Nature* **603**, 926–933 (2022).
2. Bergen, V., Lange, M., Peidli, S., Wolf, F. A. & Theis, F. J. Generalizing RNA velocity to transient cell states through dynamical modeling. *Nat. Biotechnol.* **38**, 1408–1414 (2020).
3. Smith, S. R. *et al.* Contributions of total body fat, abdominal subcutaneous adipose tissue compartments, and visceral adipose tissue to the metabolic complications of obesity. *Metabolism.* **50**, 425–435 (2001).
4. Palmer, B. F. & Clegg, D. J. The sexual dimorphism of obesity. *Mol. Cell. Endocrinol.* **402**, 113–119 (2015).
5. Reshef, Y. A. *et al.* Co-varying neighborhood analysis identifies cell populations associated with phenotypes of interest from single-cell transcriptomics. *Nat. Biotechnol.* **40**, 355–363 (2022).
6. Yang Loureiro, Z., Solivan-Rivera, J. & Corvera, S. Adipocyte Heterogeneity Underlying Adipose Tissue Functions. *Endocrinology* **163**, bqab138 (2022).
7. Shan, B. *et al.* Multilayered omics reveal sex- and depot-dependent adipose progenitor cell heterogeneity. *Cell Metab.* **34**, 783-799.e7 (2022).
8. Jeffery, E. *et al.* The Adipose Tissue Microenvironment Regulates Depot-Specific Adipogenesis in Obesity. *Cell Metab.* **24**, 142–150 (2016).
9. Romeo, S. *et al.* Genetic variation in PNPLA3 confers susceptibility to nonalcoholic fatty liver disease. *Nat. Genet.* **40**, 1461–1465 (2008).
10. Ferrero, R. *et al.* A human omentum-specific mesothelial-like stromal population inhibits adipogenesis through IGFBP2 secretion. *Cell Metab.* **36**, 1566-1585.e9 (2024).

11. Kim, H. Y. *et al.* Multi-modal analysis of human hepatic stellate cells identifies novel therapeutic targets for metabolic dysfunction-associated steatotic liver disease. *J. Hepatol.* **82**, 882–897 (2025).

12. Hao, Y. *et al.* Dictionary learning for integrative, multimodal and scalable single-cell analysis. *Nat. Biotechnol.* **42**, 293–304 (2024).

13. Kang, H. M. *et al.* Multiplexed droplet single-cell RNA-sequencing using natural genetic variation. *Nat. Biotechnol.* **36**, 89–94 (2018).

14. Yang, S. *et al.* Decontamination of ambient RNA in single-cell RNA-seq with DecontX. *Genome Biol.* **21**, 57 (2020).
