# Supplementary material for: Decreased degree of adipocyte differentiation in visceral adipose tissue contributes to metabolic dysfunction-associated steatotic liver disease
Source: Nat Commun. 2026 Jun 3;17:7101. doi: 10.1038/s41467-026-73660-6 (PMC13392065; doi:10.1038/s41467-026-73660-6)
Supplement: Supplementary file 2 — Description Of Additional Supplementary File [file 41467_2026_73660_MOESM2_ESM.pdf]

Description of Additional supplementary file

Supplementary Data 1: Clinical characteristics of MAFALDA 1 and 2 VAT and KOBS SAT snRNA-seq cohorts. excel

Supplementary Data 2: Summary statistics of adjusted latent time in adipocytes comparing individuals with MASLD and non-steatotic liver in MAFALDA 1.

Supplementary Data 3: Summary statistics of adjusted latent time in adipocytes comparing individuals with MASH and non-steatotic liver in MAFALDA 1.

Supplementary Data 4: Summary statistics of adjusted latent time in adipocytes comparing individuals with MASLD and non-steatotic liver in MAFALDA 2.

Supplementary Data 5: Summary statistics of adjusted latent time in adipocytes comparing individuals with MASH and non-steatotic liver in MAFALDA 2.

Supplementary Data 6: Assessing clinical features associated with MASLD in MAFALDA 2 using the two-sided Wald test.

Supplementary Data 7: Summary statistics of adjusted latent time in adipocytes comparing individuals with MASLD and non-steatotic liver in KOBS.

Supplementary Data 8: Summary statistics of adjusted latent time in adipocyte subtypes comparing individuals with MASLD and non-steatotic liver in KOBS.

Supplementary Data 9: Summary statistics of adjusted latent time in adipocyte comparing males and females in KOBS.

Supplementary Data 10: Summary statistics of adjusted latent time in adipocyte subtypes comparing males and females in KOBS.

Supplementary Data 11: Summary statistics of adjusted latent time in adipocytes comparing males and females in MAFALDA 2.

Supplementary Data 12: Summary statistics of adjusted latent time in adipocyte subtypes comparing males and females in MAFALDA 2.

Supplementary Data 13: Significant Bonferroni adjusted p0.25 differentially expressed genes by condition in VAT adipose stem and progenitor cells ASPCs in MAFALDA 2 using hurdle model approach in FindMarkers.

Supplementary Data 14: Significant Bonferroni adjusted p0.25 differentially expressed genes by condition in SAT adipose stem and progenitor cells ASPCs in KOBS using hurdle model approach in FindMarkers.

Supplementary Data 15: Significantly FDR0.25 by MASLD in VAT and SAT adipose stem and progenitor cells ASPCs in MAFALDA 2 and KOBS.

Supplementary Data 16: Significantly FDR0.25 by sex in VAT and SAT adipose stem and progenitor cells ASPCs in MAFALDA 2 and KOBS.

Supplementary Data 17: Significant Bonferroni adjusted p0.25 marker genes of adipose stem and progenitor cell ASPC subtype in VAT ASPCs in MAFALDA 2 determined via the two-sided Wilcoxon rank sum test in FindAllMarkers.

Supplementary Data 18: Significant Bonferroni adjusted p0.25 marker genes of Louvain clusters in VAT adipose stem and progenitor cells ASPCs in MAFALDA 2 determined via the two-sided Wilcoxon rank sum test in FindAllMarkers.

Supplementary Data 19: Significantly FDR0.25 marker genes of adipose stem and progenitor cell ASPC subtypes in SAT ASPCs in KOBS determined via the two-sided Wilcoxon rank sum test in FindAllMarkers.

Supplementary Data 20: Significant Bonferroni adjusted p0.25 marker genes of adipose stem and progenitor cell ASPC subtypes in SAT ASPCs in KOBS determined via the two-sided Wilcoxon rank sum test in FindAllMarkers.

Supplementary Data 21: Significantly FDR0.25 differentially expressed genes by MASLD in hepatocytes using hurdle model approach in FindMarkers.

Supplementary Data 22: Summary statistics of adjusted latent time including PNPLA3 in adipocytes comparing individuals with MASLD and non-steatotic liver in MAFALDA 2.

Supplementary Data 23: Summary statistics of adjusted latent time including PNPLA3 in adipocytes comparing individuals with MASH and non-steatotic liver in MAFALDA 2.

Supplementary Data 24: Summary statistics of adjusted latent time including PNPLA3 in adipocytes comparing individuals with MASLD and non-steatotic liver in KOBS.

Supplementary Data 25: Summary statistics of adjusted latent time in adipocytes comparing individuals with blood platelet measurement above and below median in MAFALDA 2.

Supplementary Data 26: Assessing relationship between blood platelet measurement and MASLD, MASH, and fibrosis in MAFALDA 2 using the two-sided Wald test.

Supplementary Data 27: Adipocyte latent time gene set that are differentially expressed in the bulk RNA-sequencing data set of human visceral adipose stem and progenitor cells ASPCs differentiated to adipocytes "subset of adipocyte latent time gene set" determined using two-sided moderated t-test.

Supplementary Data 28: Summary statistics of adjusted latent time using the subset of the latent time gene set n=666 genes in adipocytes, comparing the individuals with MASLD and non-steatotic liver in MAFALDA 2.

Supplementary Data 29: Summary statistics of adjusted latent time using the subset of the latent time gene set n=666 genes in adipocytes, comparing the individuals with MASH and non-steatotic liver in MAFALDA 2.

Supplementary Data 30: Significantly enriched functional pathways FDR0.25 differentially expressed genes by MASLD in hepatocytes using hurdle model approach in FindMarkers.

Supplementary Data 31: Summary statistics of adjusted latent time in hepatocytes, comparing the individuals with MASLD and non-steatotic liver.

Supplementary Data 32: Summary statistics of adjusted latent time in hepatocytes, comparing individuals with MASH and non-steatotic liver.

Supplementary Data 33: Significant Bonferroni adjusted p0.25 differentially expressed genes by MASLD in hepatocytes using hurdle model approach in FindMarkers.

Supplementary Data 34: Significantly FDR0.25 by MASLD that are also part of the hepatocyte latent time gene set.

Supplementary Data 35: Significantly FDR<-0.25 by MASLD that are also part of the hepatocyte latent time gene set.

Supplementary Data 36: Summary statistics of adjusted latent time in VAT adipocyte subtypes, comparing the individuals with MASLD and non-steatotic liver in MAFALDA 2.

Supplementary Data 37: Summary statistics of adjusted latent time in VAT adipocyte subtypes comparing individuals with MASH and non-steatotic liver in MAFALDA 2.

Supplementary Data 38: Significant Bonferroni adjusted p0.25 marker genes of adipocyte subtypes in VAT adipocytes in MAFALDA 2 determined via the two-sided Wilcoxon rank sum test in FindAllMarkers.

Supplementary Data 39: Significant Bonferroni adjusted p0.25 genes of the adipocyte latent time gene set n=2,000 genes that are also marker genes of adipocyte subtypes, assessed via the two-sided Wilcoxon rank sum test in FindAllMarkers, in VAT adipocytes in MAFALDA 2.

Supplementary Data 40: Significantly FDR0.25 differentially expressed genes by condition in VAT adipocytes in MAFALDA 2 using hurdle model approach in FindMarkers.

Supplementary Data 41: Significantly FDR0.25 differentially expressed genes by condition in VAT adipocytes in MAFALDA 2 using hurdle model approach in FindMarkers.

Supplementary Data 42: Gene sets used for gene set enrichment analysis.

Supplementary Data 43: Gene sets used for principal component analysis in correlation of PC1 and adipocyte diameter.

Supplementary Data 44: Genes from the four gene sets of interest.

Supplementary Data 45: LD-score LDSC based MASLD trait partitioned heritability estimates of the two gene sets in the UK Biobank n=76,758.

Supplementary Data 46: LD-score LDSC based partitioned heritability estimates of MASLD traits using the genes in the subset of the adipocyte latent time gene set n=666 genes in the UK Biobank n=76,758.

Supplementary Data 47: Significant Bonferroni adjusted p0.25 differentially expressed genes by condition in VAT adipocytes in MAFALDA 2 using hurdle model approach in FindMarkers.

Supplementary Data 48: Significant FDR<0.05 eGenes assessed by linear model implemented in Matrix eQTL that are also differentially expressed by a condition using hurdle model approach in FindMarkers.

Supplementary Data 49: Nuclei retained following each step in quality control pipeline.
