# Supplementary material for: Decreased degree of adipocyte differentiation in visceral adipose tissue contributes to metabolic dysfunction-associated steatotic liver disease
Source: Nat Commun. 2026 Jun 3;17:7101. doi: 10.1038/s41467-026-73660-6 (PMC13392065; doi:10.1038/s41467-026-73660-6)
Supplement: Supplementary file 4 — Reporting summary [file 41467_2026_73660_MOESM4_ESM.pdf]

nature portfolio

Corresponding author(s): Jussi Pihlajamäki, Stefano Romeo, Päivi Pajukanta

Last updated by author(s): May 11, 2026

Reporting Summary

Nature Portfolio wishes to improve the reproducibility of the work that we publish. This form provides structure for consistency and transparency in reporting. For further information on Nature Portfolio policies, see our Editorial Policies and the Editorial Policy Checklist.

Statistics

For all statistical analyses, confirm that the following items are present in the figure legend, table legend, main text, or Methods section.

n/a	Confirmed	
☐	☒	The exact sample size (*n*) for each experimental group/condition, given as a discrete number and unit of measurement
☐	☒	A statement on whether measurements were taken from distinct samples or whether the same sample was measured repeatedly
☐	☒	The statistical test(s) used AND whether they are one- or two-sided<br>*Only common tests should be described solely by name; describe more complex techniques in the Methods section.*
☐	☒	A description of all covariates tested
☐	☒	A description of any assumptions or corrections, such as tests of normality and adjustment for multiple comparisons
☐	☒	A full description of the statistical parameters including central tendency (e.g. means) or other basic estimates (e.g. regression coefficient) AND variation (e.g. standard deviation) or associated estimates of uncertainty (e.g. confidence intervals)
☐	☒	For null hypothesis testing, the test statistic (e.g. *F*, *t*, *r*) with confidence intervals, effect sizes, degrees of freedom and *P* value noted<br>*Give P values as exact values whenever suitable.*
☒	☐	For Bayesian analysis, information on the choice of priors and Markov chain Monte Carlo settings
☒	☐	For hierarchical and complex designs, identification of the appropriate level for tests and full reporting of outcomes
☐	☒	Estimates of effect sizes (e.g. Cohen's *d*, Pearson's *r*), indicating how they were calculated

Our web collection on statistics for biologists contains articles on many of the points above.

Software and code

Policy information about availability of computer code

Data collection	No software was used to collect the data in this study.

Data analysis

We used PLINK v1.9 for the quality control of all genotype data included in this study and performed the genotype imputation on the Michigan imputation server using Minimac4. We used STAR v2.7.10a to align the visceral adipose tissue (VAT), subcutaneous adipose tissue (SAT), liver snRNA-seq data and bulk human visceral ASPCs differentiated to adipocytes RNA-seq data. We used STAR v2.7.10b to align the VAT bulk RNA-seq. We performed quality control of the snRNA-seq data using DIEM v2.4.1, Seurat v5.0.0, DecontX from celda R package v1.16.1, demuxlet v2, DoubletFinder v2.0.3, and Harmony v1.1.0. The cell-type annotation was performed using SingleR v2.2.0. Seurat v4.3.0 and Harmony v1.1.0 were used for the liver snRNA-seq data integration and clustering. We assessed quality of fastq files using FASTQC v0.11.9. For the bulk human visceral ASPCs differentiated to adipocytes RNA-seq data, we obtained RNA metrics using Picard Tools v2.25.0 and calculated read counts with featureCounts from the subread package v2.1.0. For the bulk VAT RNA-seq, we obtained RNA metrics with PicardTools v2.25.5 and read counts using featureCounts v2.0.3. We used BisqueRNA v1.0.5 to obtain cell-type proportions in the bulk VAT RNA-seq data. Normalization of bulk RNA-seq count data was done using edgeR v3.42.4. We performed functional enrichment analysis using WebGestaltR v0.4.6, differential expression analysis using Seurat v5.0.0, MAST v1.26.0, and limma-voom v3.56.2, latent time analysis using scVelo v0.3.1 in Python v3.9.6, cellular heterogeneity assessment using the R version of CNA v0.0.99, gene overlap analysis using GeneOverlap v1.36.0, gene set enrichment analysis using MAGMA v1.10 and fgsea v1.26.0, and cis-eQTL analysis using Matrix eQTL v2.3. Differential composition analysis was performed using Wilcoxon test in R v4.3.0, linear and logistic regression analyses were performed using the Wald test in R v4.3.0, and correlation analysis was done using the spearman rank-order correlation test in R v4.3.0. We performed LD-based partition heritability analysis using LDSC v1.0.1 and performed genome-wide association studies (GWAS) using BOLT-LMM v2.3.6. No custom code was used, and all codes used for analyses in this study were based on the publicly available source code of the packages listed in the Methods. Additional details are available in the Methods.

For manuscripts utilizing custom algorithms or software that are central to the research but not yet described in published literature, software must be made available to editors and reviewers. We strongly encourage code deposition in a community repository (e.g. GitHub). See the Nature Portfolio guidelines for submitting code & software for further information.

Data

Policy information about availability of data

All manuscripts must include a data availability statement. This statement should provide the following information, where applicable:

- Accession codes, unique identifiers, or web links for publicly available datasets
- A description of any restrictions on data availability
- For clinical datasets or third party data, please ensure that the statement adheres to our policy

The MAFALDA 1 VAT snRNA-seq data are available in the NIH Gene Expression Omnibus (GEO) database under accession code GSE302702 [https://www.ncbi.nlm.nih.gov/geo/query/acc.cgi?acc=GSE302702]. The MAFALDA 2 VAT snRNA-seq data are available in the NIH GEO under accession GSE302599 [https://www.ncbi.nlm.nih.gov/geo/query/acc.cgi?acc=GSE302599]. The KOBS SAT snRNA-seq data are available in the NIH GEO under accession GSE302701 [https://www.ncbi.nlm.nih.gov/geo/query/acc.cgi?acc=GSE302701]. The liver snRNA-seq data(26) are available in the NIH GEO under accession GSE244832 [https://www.ncbi.nlm.nih.gov/geo/query/acc.cgi?acc=GSE244832]. The SAT and VAT reference snRNA-seq data from Emont et al.(19) are available for downloading in the Single Cell Portal under study number SCP1376 [https://singlecell.broadinstitute.org/single_cell/study/SCP1376]. The bulk RNA-sequencing data set of human visceral ASPCs differentiated to adipocytes(89) are publicly available in EMBL's European Bioinformatics Institute BioStudies database under ArrayExpress E-MTAB-12898 [https://www.ebi.ac.uk/biostudies/arrayexpress/studies/E-MTAB-12898?key=be3e491c-ebda-470b-9ecd-4fd6d8fcb726]. Data from the UK Biobank were used in this study under UK Biobank Application Number 33934. UK Biobank data are available for bona fide researchers through the application process [https://www.ukbiobank.ac.uk/learn-more-about-ukbiobank/contact-us]. Source data for all figures is publicly available at Zenodo(109) [https://zenodo.org/records/20127417]. Source data are provided with this paper.

Research involving human participants, their data, or biological material

Policy information about studies with human participants or human data. See also policy information about sex, gender (identity/presentation), and sexual orientation and race, ethnicity and racism.

Reporting on sex and gender

Self-reported sex information was available for all individuals from the MAFALDA, KOBS, and RYSA cohorts and was cross-checked with the genetically inferred sex from the DNA-level genotype data. We included sex as a covariate where appropriate. We also examined the cellular proportions, latent time differences, and cellular heterogeneity by sex in the MAFALDA and KOBS cohorts. Additional details are available in the Methods.

In the UK Biobank, sex was determined based on self-reporting.

In the liver snRNA-seq data, sex was determined based on self-reporting. When comparing latent time differences, we included sex as a covariate. Additional details are available in the Methods.

Reporting on race, ethnicity, or other socially relevant groupings

KOBS and RYSA study participants are from Finland and MAFALDA study participants are from Italy. All three cohorts are European.

The UK Biobank (UKB) participants in this study are individuals of European ancestry.

Population characteristics

The MAFALDA 1 visceral adipose tissue (VAT) single nucleus RNA-sequencing (snRNA-seq) bariatric surgery cohort comprises 11 Italian individuals with obesity (45% female) with a mean age of 42.47 (SD=5.14) and mean BMI of 42.58 (SD=3.82) at the time of operation, paired with histology-based liver MASLD assessment (55% MASLD). For more details, please see Supplementary Table 1.

The MAFALDA 2 VAT snRNA-seq bariatric surgery cohort comprises 63 Italian individuals with obesity (81% female) with a mean age of 43.02 (SD=9.81) and mean BMI of 41.63 (SD=3.79) at the time of operation, paired with histology-based liver MASLD assessment (70% MASLD). For more details, please see Supplementary Table 1.

The KOBS subcutaneous adipose tissue (SAT) snRNA-seq bariatric surgery cohort comprises 59 Finnish individuals with

obesity (54% female) with a mean age of 49.14 (SD=9.66) and mean BMI of 40.87 (SD=4.30) at the time of operation, paired with histology-based liver MASLD assessment (25% MASLD). For more details, please see Supplementary Table 1.

The RYSA VAT bulk RNA-seq bariatric surgery cohort comprises 66 Finnish individuals with obesity (71% female) with a mean age of 46.37 (SD=6.97) and mean BMI of 43.11 (SD=5.02) at the time of operation (Heinonen et al., Obesity (Silver Spring), 2023).

The UKB cohort consists of data collected since 2006 from over 500,000 individuals aged 37 to 73 from the UK, who were profiled across 22 different assessment centers (Bycroft et al., Nature, 2018; Sudlow et al., PLoS Medicine, 2015).

The liver snRNA-seq data consists of deidentified liver biopsies from 18 deceased individuals. Patient history including age (mean age=47.22, SD=14.43), sex (44.4% female), BMI (mean BMI=31.61, SD=7.90), and cause of death, and laboratory tests were provided with informed consent from Lifesharing OPO.

Recruitment

The MAFALDA visceral adipose tissue single nucleus RNA-sequencing (snRNA-seq) cohort comprises 74 participants (n=11 in the discovery cohort MAFALDA 1, and n=63 in the replication cohort MAFALDA 2) from the previously published Molecular Architecture of FAtty Liver Disease in individuals with obesity undergoing bAriatric surgery (MAFALDA) cohort (n=264) (Tavaglione et al., Clin Gastroenterol Hepatol., 2023; Jamialahmadi et al., Nat. Med., 2024). For this study, we obtained visceral adipose tissue (VAT) biopsy samples around the gastric cardia, genotype, and phenotype data. Consecutive individuals with morbid obesity (body mass index, BMI≥35kg/m^2) undergoing bariatric surgery were recruited at Fondazione Policlinico Universitario Campus Bio-Medico, Rome, Italy. Individuals were excluded from the study if they had a history of alcohol abuse, viral hepatitis, or other causes of liver disease.

A total of 59 individuals with severe obesity (BMI ≥35kg/m^2) undergoing bariatric surgery were included from the Kuopio OBesity Surgery study (KOBS) cohort (Benhammou et al., Hepatol Commun., 2019). For this study, we obtained subcutaneous adipose tissue (SAT) biopsy samples, genotype, and phenotype data. Individuals with obesity were recruited in the University of Eastern Finland and Kuopio University Hospital, Kuopio, Finland.

A total of 66 individuals with severe obesity (BMI ≥35kg/m^2) undergoing bariatric surgery were included from the Roux-en-Y versus one-anastomosis gastric bypass (RYSA) cohort (Heinonen et al., Obesity (Silver Spring), 2023). For this study, we obtained VAT biopsy samples, adipocyte size estimates and phenotype data. Individuals were recruited at the Helsinki University Hospital, Helsinki, Finland.

The UKB study recruited individuals of various ages and backgrounds from across the UK from 2006 to 2010, as described in detail previously (Bycroft et al., Nature, 2018; Sudlow et al., PLoS Med., 2015).

Deidentified liver biopsies from deceased individuals were declined for transplantation and obtained via the Lifesharing Organ Procurement Organization (OPO) (Kim et al., Journal of Hepatology, 2025; Liu et al., STAR Protoc, 2023).

Ethics oversight

The Molecular Architecture of FAtty Liver Disease in individuals with obesity undergoing bAriatric surgery (MAFALDA) study was approved by the Local Research Ethics Committee at the Campus Bio-Medico University Hospital of Rome (Italy) (approval no. 16/20) and by the Swedish Ethics Review Authority (Dnr 2025-08073-01 and Dnr 2025-06169-01). The Kuopio OBesity Surgery Study (KOBS) study was approved by the Ethics Committee of the Northern Savo Hospital District (54/2005, 104/2008, and 27/2010). The Roux-en-Y versus one-anastomosis gastric bypass (RYSA) cohort was approved by the Helsinki University Hospital Ethics Committee (HUS/1706/2016). The UK Biobank study was approved by the North West Multi-centre Research Ethics Committee (21/NW/0157). All participants provided written informed consent, and no compensation was provided to the participants. All research conforms to the principles of the Declaration of Helsinki.

Note that full information on the approval of the study protocol must also be provided in the manuscript.

Field-specific reporting

Please select the one below that is the best fit for your research. If you are not sure, read the appropriate sections before making your selection.

☒ Life sciences ☐ Behavioural & social sciences ☐ Ecological, evolutionary & environmental sciences

For a reference copy of the document with all sections, see nature.com/documents/nr-reporting-summary-flat.pdf

Life sciences study design

All studies must disclose on these points even when the disclosure is negative.

Sample size

No statistical method was used to predetermine the sample size. For VAT snRNA-seq data (total n=74; MAFALDA 1 n=11 and MAFALDA 2 n=63) and SAT snRNA-seq data (n=59 in the KOBS cohort), we used all available snRNA-seq data with corresponding liver histology data. The RYSA cohort comprises a total of 66 individuals with both VAT bulk RNA-sequencing data and adipocyte size estimates. The liver snRNA-seq data comprises a total of 18 deceased individuals with liver phenotype data.

The single cell level sample sizes of the main cohorts (MAFALDA and KOBS) are larger than or similar to those reported in previous cell-type level SAT and VAT studies [PMID: 35296864, PMID: 39856219, PMID: 40634602]. Based on our literature search, there are currently no larger VAT snRNA-seq data available in previous studies than the VAT snRNA-seq data used here. We also validated the adipocyte latent time results of MAFALDA 1 in MAFALDA 2, which suggests that the sample sizes are sufficient.

We performed genome-wide association studies (GWASs) for the continuous serum alanine transaminase (ALT) (n=373,241), liver magnetic resonance imaging proton density fat fraction (PDFF) (n=23,606), fatty liver index (FLI) (n=372,830), and the binarized MASLD status using

PDFF (n=17,463) and binarized FLI (n= 275,467) in the unrelated European individuals of the UKB. Partitioned heritability was performed with the UK Biobank on 76,758 randomly selected, unrelated European individuals. Based on our literature search, these sample sizes are similar as in previous genomic studies of UKB data, and anticipated to be adequately powered due to the unprecedented large sample size of the cohort.

Data exclusions — Related individuals were excluded from all cohorts. Additional details are available in the Methods.

Replication — We used VAT single nucleus RNA-sequencing to examine adipocyte latent time results from a discovery cohort of 11 individuals, referred to as MAFALDA 1. We validated the adipocyte latent time results of MAFALDA 1 in a cohort of 63 independent individuals, referred to as MAFALDA 2. We performed all other analyses in the larger cohort of 63 independent individuals, MAFALDA 2, and did not perform further analyses in MAFALDA 1 due to its sample size of 11 individuals.

Results reported from the UKB GWAS analyses were not replicated due to the unprecedented large sample size of the cohort.

Randomization — N/A. This is an observational study, so no randomization was performed.

Blinding — N/A. This is an observational study, so no blinding was performed.

Reporting for specific materials, systems and methods

We require information from authors about some types of materials, experimental systems and methods used in many studies. Here, indicate whether each material, system or method listed is relevant to your study. If you are not sure if a list item applies to your research, read the appropriate section before selecting a response.

Materials & experimental systems

n/a	Involved in the study
☒	☐ Antibodies
☒	☐ Eukaryotic cell lines
☒	☐ Palaeontology and archaeology
☒	☐ Animals and other organisms
☒	☐ Clinical data
☒	☐ Dual use research of concern
☒	☐ Plants

Methods

n/a	Involved in the study
☒	☐ ChIP-seq
☒	☐ Flow cytometry
☒	☐ MRI-based neuroimaging

Plants

Seed stocks — *Report on the source of all seed stocks or other plant material used. If applicable, state the seed stock centre and catalogue number. If plant specimens were collected from the field, describe the collection location, date and sampling procedures.*

Novel plant genotypes — *Describe the methods by which all novel plant genotypes were produced. This includes those generated by transgenic approaches, gene editing, chemical/radiation-based mutagenesis and hybridization. For transgenic lines, describe the transformation method, the number of independent lines analyzed and the generation upon which experiments were performed. For gene-edited lines, describe the editor used, the endogenous sequence targeted for editing, the targeting guide RNA sequence (if applicable) and how the editor was applied.*

Authentication — *Describe any authentication procedures for each seed stock used or novel genotype generated. Describe any experiments used to assess the effect of a mutation and, where applicable, how potential secondary effects (e.g. second site T-DNA insertions, mosiacism, off-target gene editing) were examined.*
