# Supplementary material for: Decreased degree of adipocyte differentiation in visceral adipose tissue contributes to metabolic dysfunction-associated steatotic liver disease
Source: Nat Commun. 2026 Jun 3;17:7101. doi: 10.1038/s41467-026-73660-6 (PMC13392065; doi:10.1038/s41467-026-73660-6)
Supplement: Supplementary file 5 — Transparent Peer Review file [file 41467_2026_73660_MOESM5_ESM.pdf]

nature portfolio

Peer Review File

Decreased degree of adipocyte differentiation in visceral adipose tissue contributes to metabolic dysfunction-associated steatotic liver disease

Corresponding Author: Professor Päivi Pajukanta

This file contains all reviewer reports in order by version, followed by all author rebuttals in order by version.

Version 0:

Reviewer comments:

Reviewer #1

(Remarks to the Author)
In this study, Gelev et al. aimed to explore the relationship between adipogenic capacity driving adipose tissue hyperplastic expansion and ectopic fat accumulation contributing to MASLD pathogenesis. Using single nucleus RNA sequencing data from human adipose tissue, the authors introduced the concept of "adipogenesis differentiation rates" (ADR) to assess adipogenic capacity in individuals with and without MASLD. Their findings revealed an inverse association between adipose tissue ADR and MAFLD, particularly in visceral adipose tissue. They also identified distinct sex-based differences in ADR. Genetic analyses highlighted regional variations in ADR genes that significantly explained MASLD heritability, underscoring the genetic impact of ADR on MASLD.
This well-conducted study utilized state-of-the-art single nucleus analysis and robust bioinformatic techniques to contribute to understanding adipose tissue remodeling in MASFD pathogenesis. Aligning with recent advancements in adipose tissue expandability, the study provides new evidence supporting the emerging concept that enhanced de novo adipogenesis and hyperplastic adipose expansion may confer metabolic protection against metabolic liver diseases.
Several points could strengthen the manuscript upon revision:
1. The core metric used, ADR, evaluates adipogenic capability based on genes associated with adipogenesis, predominantly from the single-nuc seq data of the cluster defined as mature adipocytes and/or terminally differentiating cells. With respect to de novo adipogenesis, it would be beneficial to extend the analysis to include changes in precursor cells, as seen in rodent studies (PMID: 33378646).
2. ADR is composed of over 2000 genes (Supplemental Table 4), many of which are involved in general adipocyte functions rather than specifically adipogenic differentiation. Understanding the relevance of these genes to adipogenesis per se, versus general adipocyte biology influenced by environmental signals, could refine interpretations of differentiation status and functional heterogeneity within adipocytes.
3. Figure 2K highlights enrichment in early progenitor pathways such as ECM and chemotaxis. While these pathways contribute to adipose tissue remodeling and metabolic health, their selective relevance to adipocyte progenitors and adipogenesis merits further clarification.
4. Addressing sex differences beyond mature adipocytes to include adipose progenitors would enhance the understanding of sex dimorphism in adipose tissue expandability, as previous work has suggested (PMID: 27320063,35447091).
5. Further exploration of enriched pathways beyond adipogenesis, such as fibrosis and inflammation, could provide broader insights into MASLD pathophysiology and optimize data interpretation.

Reviewer #2

(Remarks to the Author)
It is well established that defects in adipogenesis (e.g., in lipodystrophies) result in the accumulation of neutral lipids in the liver. In the manuscript under consideration, Gelev et al. hypothesize that analysis of single nucleus gene expression in visceral adipose tissue will uncover cell types and genes that connect defects in “adipogenesis” and “adipocyte differentiation” to the development of MASLD.
While this manuscript is a worthy effort, the main problem is that the computational definition of ‘adipogenesis’ is based on an unproved premise (lacking experimental validation and logically circular), and contradicts accepted notions of

adipogenesis.
The concept of adipogenesis used in the manuscript is based on the ratio of unspliced to spliced mRNAs. While this approach could be applied to clear differentiation trajectories, as detailed in ref 53, its application to mature adipocytes is not validated or clearly explained. The calculation of 'latent time' is taken to comprise several concepts including 'adipogenesis' , 'adipocyte differentiation', 'adipogenesis rate', and a cell's 'biological age'. None of these concepts is experimentally validated or has a coherent biological basis. The authors do not present a trajectory or transition pathway for supposed adipogenesis. As typically understood, the process of adipogenesis is the development of adipocytes from adipo-fibroblast progenitors. Here the authors are evaluating mRNA splicing in fully differentiated adipocytes. At most, the authors show that under some conditions and after extensive normalization, the ratio of unspliced to spliced mRNA is reduced in certain clusters of differentiated cells. Whether this splicing pattern has anything to do with adipocyte differentiation or function remains unclear.
The authors state that they obtained "highly expressed, specific marker genes for each subtype"….
and defined unique marker genes as being only present in one subtype." Presumably, the list of unique genes is presented in Suppl table 3. However, it is difficult to conceive that IRS1, PPARG, PPARA, STAT3, and ABHD5 are unique to hAd2 as seems to be claimed. If these are differentially expressed (not unique), please list the magnitude, % of cells expressing, etc.

Did the authors consider common genetic variants of MASLD that are expressed in adipocytes (PNPLA3, TM6SF2, MBOAT7) between the non-steatotic and MASLD patients? These may confound interpretation of scRNAseq data as variants in these genes are known to promote MASLD through reductions in VLDL TAG secretion in the liver and not necessarily defects due to adipose function.

Other concerns:
• The authors state "Subtype hAd2 comprises pathways specific to lipid metabolism"; Table 3 lists >340 GO terms the vast majority of which have nothing to do with lipid metabolism. While it is true that the top 10 GO terms by fold-enrichment are involved in lipid metabolism, this enrichment is biased by the source (adipocytes), and the same genes are represented in seemingly contradictory pathways (GO:0010888 GO:0010889).
• How is blood platelet count a negative control? How is latent time calculated in cells that don't have a nucleus?
• What about other cell types in the snRNA set, particularly ASPCs.
• Show the Louvain clustering in figure 1d
• The same non-steatotic controls seem to be used in multiple comparisons.
• What genes in Figure 2H, I are DE between subtypes?
• The meaning of figure 2J is unclear.
• The calculation of subtype proportion does not map onto figure 2g. Please provide the list of raw and inverse-normalized subtype proportions.
• How were cells clustered in figure 1 and does this clustering agree with clustering in figure 2?
• The manuscript could use editing from a native English speaker. For examples the first sentence in the abstract and run on statement (pg 23 lines 21) is ambiguous.
• How is a log2FC>0.25 for DEs considered biologically relevant?

Reviewer #3

(Remarks to the Author)
I co-reviewed this manuscript with one of the reviewers who provided the listed reports. This is part of the Nature Communications initiative to facilitate training in peer review and to provide appropriate recognition for Early Career Researchers who co-review manuscripts.

Version 1:

Reviewer comments:

Reviewer #1

(Remarks to the Author)
The reviewer commends the authors for a thoughtful and thorough revision, which has significantly strengthened the manuscript. The clarification of the terms "adipocyte differentiation" and "adipocyte maturation" is appreciated, particularly given the study's focus on cell clusters at the terminal stage of adipogenic differentiation. The additional data analyses have further reinforced the evidence supporting the study's main conclusions. All previously raised concerns have been appropriately addressed, and no major issues remain.

Reviewer #2

(Remarks to the Author)
The authors show that adipocytes of 'non-steatotic' controls have a different splicing pattern ('latent time) than subjects with steatosis, and that this phenomenon has a significant heritable component. This pattern is consistent with the widely held view that defective adipocyte function contributes to hepatosteatosis. The data are interesting and could advance the field.

However, several key problems remain in this revision:
1) Direction of causality. It is unclear whether one would find the same pattern of RNA splicing (latent time) in hepatocytes or other metabolic tissues.

2) Figure 5 implies that hepatosteatosis causes alterations in adipocyte gene expression (e.g., downregulated by MASLD). The point of the paper is the reverse causation, the validity of which is uncertain.
3) The original review raised the central concern that the concept of 'latent time' as it relates to adipocyte biology has no experimental validation or coherent biological basis. Relabeling 'adipogenesis' as "adipocyte maturation" does not address this conceptual flaw. The introductory statement : "We hypothesized that impaired VAT adipocyte maturation impacts metabolic dysfunction associated steatotic liver disease (MASLD)." is circular reasoning. It should be noted that the computational method for latent time was used to address splicing ratios in a very short time frame (hours/days). It is unclear what "adipocyte maturation' means when the temporal sampling domain is essentially random, and the lifespan of a human adipocyte is many months, if not years.
4) The latent time analysis depends heavily on a limited number of control comparisons, and the methods section is unclear as to the source and phenotypes of the non-steatotic controls, and when and how these control samples were obtained and processed. Were the controls part of consecutive bariatric surgeries that were obese, but not steatotic, as implied? The authors must explicitly detail how and when the non-steatotic controls were obtained and processed for sn-RNAseq since all of the comparisons are based on this relatively small set of controls. A reader can't extract the relevant information from references 24,25. It is unclear how the numbers in table 1 align with the description in the methods.
5) What is meant by replication? According to the text and table 1 MAFALDA1 has 11 subjects (10 with steatosis). It is not obvious what controls were used (see comment 4). What specific genes were replicated? Or was it the phenomenon of splicing ratios, without reference to specific genes?

Version 2:

Reviewer comments:

Reviewer #4

(Remarks to the Author)
Review NCOMMS-25-17078B
Gelev et al report on single-nucleus RNA seq from SQ and visceral adipose tissue, and report that gene expression profiles differ in those individuals with MASLD vs. those without. Moreover, individuals with liver disease are more likely to have gene expression patterns suggesting less differentiated adipocytes based on trajectory analysis. The strengths of the manuscript include the 3 distinct cohorts, integration of liver metabolic data from the adipose donors, the robust analysis of gene expression using snRNA-seq, and the integration of DNA sequencing for eQTL analysis. Weaknesses of the manuscript include the lengthy methods section, which is laborious to read and distracts from the results section – it could be substantially shortened and/or some of the methods moved to supplementary files. The authors do not provide information about the metabolic characterization of the biopsy donors beyond age, BMI and presence or absence of MASLD and DM. It would be important to assess relationships between the findings to key clinical variables such as age, BMI and glycemic control because these phenotypic variables could also contribute to both MASLD and the adipose gene expression phenotypes, a key analysis when trying to assess direction of causality. A major concern is that the authors indicate conclusions of causality in many sections of the manuscript, when their cross sectional analysis cannot. This point was raised by reviewer 2 but incompletely addressed.
The authors have been somewhat responsive to the prior critiques, adding the trajectory analysis to the liver samples – finding that the pattern does not mirror that of the adipocytes. Moreover, some of the points in the response to review have not been adequately added to the manuscript itself.
Major comments:
Within the text and figures, it is not very clear that the MASH group is a subset of the MASLD group. This should be clearly spelled out very early in the results section.
The authors use the term "adipocyte maturation rate" – this is misleading. There is no time-dependent variable in this cross-sectional analysis. Likewise, latent time is not clear in this context. A different label would be more precise. As pointed out by the prior reviewer 2, the trajectory analysis is predicting degree of differentiation. In the case of adipocytes, it does not mean that this is a currently ongoing process, just that the adipocytes are in different states at the time of the biopsy sampling. The authors should remove language suggesting this is a dynamic ongoing process throughout the manuscript.
Differences in "adipocyte maturation rate" between non-steatotic and MASLD (box plots of Figures 1-4 (e.g. 1e, 2c, 3b, 4d, 4e, etc.)) are significant due to the large number of nuclei being assessed, but the actual magnitude of difference appears to be small but unclear. Can the authors provide this information e.g. provide difference in units corresponding to the actual trajectory analysis (which is scaled from 0 to 1)? Can the authors graph latent time data for each individual within the group?
The authors indicate (line 548) that the overall reduction in latent time in VAT adipocytes is driven by the hAd2 subtype. I don't think the authors have provided evidence for this – it remains a hypothesis and association. Similarly, the authors conclude that sex is driving the heterogeneity of VAT adipocytes (line 593) and contributes to VAT maturation and functon(line 596) but don't provide sufficient evidence to support these statements beyond association. Likewise, line 635 indicates sex has a profound effect on rate of adipocyte maturation in SAT – association, not cause-effect relationship. Please modify these conclusions.
Can the authors clarify what percentage of adipocyte maturation genes which are differentially expressed in donors with MASLD are also expressed in hepatocytes, and are these differentially expressed in MASLD as well? This is important to interpret the results of the eQTL analysis and assessment of heritability as well.
The authors note (line 616) that VAT adipocyte proportions change with progression of MASLD into MASH. The cross-sectional data do not allow this conclusion. Please revise the description of these results throughout. Association does not mean causation.
Minor comments:

- Why was the MT threshold was set to >10%? Typically, single-cell studies use MT>10%, but most single-nucleus studies set it >5% or even >1%.
- The LISI score should be calculated based on the Harmony integration that was performed.
- Figure 1c colors are not clear – if the purple is just meant to show adipocytes, then it is not a helpful figure.
- The colors in Suppl. Figure 1b, Suppl. Figure 3b-c, and Suppl. Figure 10b-c are too similar and it is not easy to distinguish each sample. A stacked BarPlot per sample should be used instead.
- Point 3 of previous reviewer#2 has not been addressed properly. Indeed, there has been no experimental validation or coherent biological basis in this study, which is required to support the statements of this paper. Specifically, what does reduced latent time mean for these adipocytes? Are single-nuclei datasets appropriate for such kind of analysis that depends on splicing, or would single-cell data be more appropriate? Have the adipocytes stopped expanding and are unable to store more lipids and/or have they reached maximum size? In order to further test this hypothesis using the cross sectional data in the current study, additional information would be needed. Based on transcriptomic markers marking the adipocytes with "reduced latent time", can the authors identify and validate (e.g. via IHC) adipocytes with reduced latent time and make statements regarding their cell size and/or morphology?
- The authors should further expand on the potential functional meaning of the increased latent time for hepatocytes in individuals with MASLD (lines 519-520)? What were the gene expression patterns characteristic of the hepatocytes with increased latent time? I may have missed it but did not see this.
- Since Latent Times of adipocytes between the two groups are different, a question arises whether new adipocytes can be recruited from adipogenic progenitors (ASPCs). Perhaps the authors could mention this as a possibility for future study.
- Page 7, line 167 – what does "final concentrations of nuclei" mean? Please provide # of nuclei loaded.
- Were UMI cutoffs the same for each sample?
- Please provide the number of nuclei removed at each step. It would be helpful to include a diagram of the analysis pipeline – this may also allow the authors to summarize the analysis and move some of the details to supplementary methods.
- Please explain why platelet count was used as a "negative phenotypic control." (line 497). One might anticipate that platelet counts differed in MASLD or fibrosis.
- Volcano plot labels cover up the actual data - please revise.

Version 3:

Reviewer comments:

Reviewer #4

(Remarks to the Author)
In this revision, Gelev et al have responded well to the prior review. They have toned down the description of results to emphasize the potential nature of the putative relationships identified in this cross-sectional analysis. They have added an interesting new analysis investigating adipocyte cell size in relationship to adipocyte differentiation.

Open Access This Peer Review File is licensed under a Creative Commons Attribution 4.0 International License, which permits use, sharing, adaptation, distribution and reproduction in any medium or format, as long as you give appropriate credit to the original author(s) and the source, provide a link to the Creative Commons license, and indicate if changes were made.

In cases where reviewers are anonymous, credit should be given to 'Anonymous Referee' and the source.
The images or other third party material in this Peer Review File are included in the article’s Creative Commons license, unless indicated otherwise in a credit line to the material. If material is not included in the article’s Creative Commons license and your intended use is not permitted by statutory regulation or exceeds the permitted use, you will need to obtain permission directly from the copyright holder.
To view a copy of this license, visit https://creativecommons.org/licenses/by/4.0/

Responses to Reviewers

Responses to Reviewer #1

We thank the Reviewer for the helpful comments and suggestions on the manuscript and have addressed all questions that were raised. We hope that the revisions satisfactorily respond to all of the Reviewer's questions. On behalf of the authors, we feel that the revised manuscript is significantly strengthened based on these astute comments. The numbering of the revised pages we provide below refer to the tracked changes version of the revised manuscript. We also provide a clean manuscript. In our responses, we refer to previous papers using their PubMed IDs.

Reviewer #1:

In this study, Gelev et al. aimed to explore the relationship between adipogenic capacity driving adipose tissue hyperplastic expansion and ectopic fat accumulation contributing to MASLD pathogenesis. Using single nucleus RNA sequencing data from human adipose tissue, the authors introduced the concept of "adipogenesis differentiation rates" (ADR) to assess adipogenic capacity in individuals with and without MASLD. Their findings revealed an inverse association between adipose tissue ADR and MAFLD, particularly in visceral adipose tissue. They also identified distinct sex-based differences in ADR. Genetic analyses highlighted regional variations in ADR genes that significantly explained MASLD heritability, underscoring the genetic impact of ADR on MASLD.
This well-conducted study utilized state-of-the-art single nucleus analysis and robust bioinformatic techniques to contribute to understanding adipose tissue remodeling in MASFD pathogenesis. Aligning with recent advancements in adipose tissue expandability, the study provides new evidence supporting the emerging concept that enhanced de novo adipogenesis and hyperplastic adipose expansion may confer metabolic protection against metabolic liver diseases.
Several points could strengthen the manuscript upon revision:

1. The core metric used, ADR, evaluates adipogenic capability based on genes associated with adipogenesis, predominantly from the single-nuc seq data of the cluster defined as mature adipocytes and/or terminally differentiating cells. With respect to de novo adipogenesis, it would be beneficial to extend the analysis to include changes in precursor cells, as seen in rodent studies (PMID: 33378646).

1. Response: We thank the Reviewer for these helpful and insightful comments on our findings. We recognize that our claim of "delayed adipocyte differentiation (i.e., adipogenesis) rate" may not have been accurate enough. We have now changed it to "delayed adipocyte maturation rate" throughout the manuscript, including the title, to improve accuracy and better highlight our

findings in human VAT adipocytes. Furthermore, as our analysis does not incorporate precursor cells similarly as the previous rodent studies have included [PMID 33378646], this renaming seems to be most appropriate given the context. Nevertheless, we believe that our results in human VAT adipocytes are important and further strengthened by the significant overlap between our adipocyte maturation rate genes and the VAT adipogenesis differentially expressed (DE) genes that we detected in our new analysis (please see our response to point 2 below). Furthermore, our study provides insight into how human adipocyte maturation is related to the pathogenesis of MASLD. The lower maturation rate in adipocytes may reflect an induction of pro-fibrotic behavior that restrains their development into fully differentiated adipocytes. A previous study investigated the preadipocyte-to-adipocyte transition and highlighted non-classical adipocytes as being an intermediate step to fully differentiated classical adipocytes [PMID 39856219]. Based upon their findings, we postulate that we are observing a similar effect, however, now showing that individuals with MASLD remain in this intermediate subcellular stage (i.e., adipocyte subtype hAd1). Importantly, the marker genes of subtype hAd1 are characterized by pathway enrichments of fibrotic behavior, such as angiogenesis, immune response, and extracellular matrix organization [PMID 39856219], rather than fully transitioning to a classical adipocyte phenotype (i.e., adipocyte subtypes hAd2 and hAd6). We have now revised the manuscript to add these insights to the Discussion section (page 37, lines 1-5).

2. ADR is composed of over 2000 genes (Supplemental Table 4), many of which are involved in general adipocyte functions rather than specifically adipogenic differentiation. Understanding the relevance of these genes to adipogenesis per se, versus general adipocyte biology influenced by environmental signals, could refine interpretations of differentiation status and functional heterogeneity within adipocytes.

2. Response: We thank the Reviewer for this helpful comment. We have now performed a differential gene expression analysis leveraging a previously published dataset of human visceral ASPCs differentiated to adipocytes from the omentum [PMID 38729152] to provide more insight into our adipocyte maturation rate gene set (Supplementary Table 2). We found a significant overlap ($p=0.002$) between our VAT adipocyte maturation rate gene set and differentially expressed VAT adipogenesis genes, resulting in 666 overlapping genes. Moreover, taking those 666 genes, we re-performed the analysis with scVelo [PMID 32747759] and found similar significant results as with the full adipogenesis maturation rate gene set (2,000 genes) (new Supplementary Figure 5). Accordingly, the individuals with MASLD have a lower latent time when compared to the individuals without MASLD ($p=4.9x10^{-31}$). This lower latent time was also observed in the individuals with MASH in comparison to the individuals without MASLD ($p=2.1x10^{-14}$). Furthermore, the 666 genes that overlapped between the differentially expressed genes by VAT ASPC differentiation and the adipocyte maturation rate gene set are enriched for pathways in morphogenesis, development, and activin receptor signaling (new Supplementary Figure 5 and new Supplementary Table 3). These new findings further support

the role of delayed adipocyte maturation rate in MASLD progression. Finally, we also found that the 666 genes still explained a significant proportion of heritability in ALT and FLI (new Supplementary Figure 16), further emphasizing the finding that adipocyte maturation is under genetic regulation and contributes to MASLD associated traits. We have now revised the manuscript to add these results to the revised Methods (page 12, lines 2-4, 12-23); page 13, lines 1-6; page 14, lines 17-20; page 18, lines 9-14), Results (page 22, lines 9-22; page 31, lines 2-5), new Supplementary Figures 5 and 16, and a new Supplementary Tables 2 and 3.

3. Figure 2K highlights enrichment in early progenitor pathways such as ECM and chemotaxis. While these pathways contribute to adipose tissue remodeling and metabolic health, their selective relevance to adipocyte progenitors and adipogenesis merits further clarification.

3. Response: We thank the Reviewer for this insightful comment. We have now added additional information from previously published rodent studies [PMID10691039, 27879248] and in vitro studies [PMID 16678100] into the revised Results (page 24, line 23; page 25, lines 1-9) to clarify the role of the extracellular matrix (ECM) and chemotaxis in adipocyte differentiation. Briefly, both ECM organization and chemotaxis have been implicated in adipogenesis [PMID 20107860, 32047213]. For example, chemotaxis refers to the movement of cells in response to a chemical stimulus. In the context of adipogenesis, this may be a result of growth factors such as *FGF2*, *VEGFC*, and *ANGPT1*, which we found as marker genes for subtype hAd1. These genes act as regulators of adipogenesis [PMID 25790378] and modulate angiogenesis [PMID 32616854, 32146252, 14623307]. The ECM is crucial for structure, preadipocyte differentiation, and intercellular communication [PMID 8880414, 20107860, 21366833]. It also plays a part in adipocyte maturation and plasticity [PMID 10508658]. The adipocyte subtype that we highlight for MASH, hAd1, features marker genes involved in both ECM organization and chemotaxis. Considering this previous literature, the enrichment of these pathways suggests a state of underdevelopment, exhibiting more preadipocyte-specific functions. Coupled with the delayed latent time, these adipocytes appear to remain as underdeveloped adipocytes that express more fibroblastic-like markers and are unable to reach their full adipogenic potential. We have now added these points to the revised Results (page 24, line 23; page 25, lines 1-9) and Discussion (page 36, lines 1-2).

4. Addressing sex differences beyond mature adipocytes to include adipose progenitors would enhance the understanding of sex dimorphism in adipose tissue expandability, as previous work has suggested (PMID: 27320063,35447091).

4. Response: We thank the Reviewer for this helpful comment. As suggested by the Reviewer, we have now conducted additional analyses investigating adipose progenitor cells, ASPCs. Although we did not find the proportions of adipose progenitor cell subtypes to differ by sex in

either VAT or SAT, we did perform a differential expression (DE) analysis and assessed whether the marker genes of these ASPC subtypes differed by sex, similar to the animal study mentioned by the Reviewer, which investigated adipose progenitor cells in mice and their functional differences by sex [PMID 35447091]. We identified a total of 123 genes in VAT and 349 genes DE by sex in SAT (Supplementary Table 8, Supplementary Table 9). Of these, in VAT, only 13 DE genes by sex overlapped with the marker genes of the VAT hASPC2 subtype, 15 genes overlapped with the marker genes of the VAT hASPC3 subtype, and 3 genes overlapped with the marker genes of the VAT hASPC4 subtype (Supplementary Table 12). We did, however, find that there are some Louvain clusters within ASPC subtypes that are enriched for sex-specific pathways, such as ovulation cycle and sex differentiation (Supplementary Table 13, Supplementary Figure 14, Supplementary Table 14), suggesting the influence of sex in VAT adipose progenitor cells as well. Furthermore, the presence of these pathways also highlights the potential role of sex hormones in modulating human VAT adipose progenitor cells, in line with the previous mouse study [PMID 27320063].

On the other hand, in SAT, 54 genes DE by sex overlapped with the marker genes of the SAT hASPC4 subtype and 90 genes DE by sex overlapped with the marker genes of the SAT hASPC5 subtype (new Supplementary Table 15). The 90 marker genes in the SAT hASPC5 subtype are enriched for collagen processes and extracellular matrix and structure (Supplementary Figure 15, Supplementary Table 16), implicating modulated adipogenesis by sex in humans, in line with the previous mouse study mentioned by the Reviewer [PMID 35447091]. This larger number of the sex DE genes in SAT ASPC subtypes supports our other SAT findings (Figure 4), showing that SAT adipose tissue is greatly impacted by sex. However, neither the VAT ASPC subtype proportions nor the SAT ASPC subtype proportions differed by the MASLD status (Supplementary Figure 11). We have now added these results to the revised Methods (page 11, line 2; page 17, lines 17-21), Results (page 27, line 23; page 28, lines 1-9, 17-21; page 29, lines 1-17), Discussion (page 37, lines 15-19), new Supplementary Figures 11, 14, and 15, and new Supplementary Tables 8, 9, 12, 13, 14, 15, 16.

5. Further exploration of enriched pathways beyond adipogenesis, such as fibrosis and inflammation, could provide broader insights into MASLD pathophysiology and optimize data interpretation.

5. Response: We thank the Reviewer for this insightful comment. The pathways, such as chemotaxis and ECM, that we found among the marker genes of hAd1, have also been reported as indicators of adipocyte dysfunction [PMID 22947075, 36715281]. Chemotaxis has been implicated in recruiting immune cell-types, such as macrophages, and inducing inflammation in adipose tissue [PMID 32699606]. Chronic inflammation can induce tissue fibrosis. Increased ECM remodeling has also been observed as an indicator of unhealthy expansion of adipocytes, causing hypoxia and fibrosis [PMID 36715281]. Therefore, the presence of these pathways

among the marker genes of hAd1 potentially reveals increased fibrogenesis in visceral adipose tissue, underscoring a pro-inflammatory and hypoxic microenvironment [PMID 23954640] that contributes to the development of MASLD. VAT adipocytes in individuals with MASLD may be reduced in their capacity to become fully differentiated adipocytes due to this microenvironment. Thus, these adipocytes are likely delayed in their maturation, hampering their ability to store fatty acids, exacerbating to the accumulation of triglycerides in the liver and inducing MASLD. Therefore, our findings on the differences in the proportions of adipocyte subtypes hAd1 and hAd2 by the MASH status and the delayed latent time in both MASLD and MASH present a potential mechanism for MASLD pathogenesis originating from visceral adipose tissue. We have now added these additional details to the revised Discussion (page 36, lines 1-10, 21-23; page 37, lines 5-8) to optimize data interpretation as suggested by the Reviewer.

Responses to Reviewer #2

We thank the Reviewer for the helpful comments and suggestions on the manuscript and have addressed all questions that were raised. We hope that the revisions satisfactorily respond to all of the Reviewer's questions. On behalf of the authors, we feel that the revised manuscript is significantly strengthened based on these astute comments. The numbering of the revised pages we provide below refer to the tracked changes version of the revised manuscript. We also provide a clean manuscript. In our responses, we refer to previous papers using their PubMed IDs.

Reviewer 2:

It is well established that defects in adipogenesis (e.g., in lipodystrophies) result in the accumulation of neutral lipids in the liver. In the manuscript under consideration, Gelev et al. hypothesize that analysis of single nucleus gene expression in visceral adipose tissue will uncover cell types and genes that connect defects in "adipogenesis" and "adipocyte differentiation" to the development of MASLD.

Point 1. While this manuscript is a worthy effort, the main problem is that the computational definition of 'adipogenesis' is based on an unproved premise (lacking experimental validation and logically circular), and contradicts accepted notions of adipogenesis.
The concept of adipogenesis used in the manuscript is based on the ratio of unspliced to spliced mRNAs. While this approach could be applied to clear differentiation trajectories, as detailed in ref 53, its application to mature adipocytes is not validated or clearly explained. The calculation of 'latent time' is taken to comprise several concepts including 'adipogenesis', 'adipocyte differentiation', 'adipogenesis rate', and a cell's 'biological age'. None of these concepts is experimentally validated or has a coherent biological basis. The authors do not present a trajectory or transition pathway for supposed adipogenesis. As typically understood, the process of adipogenesis is the development of adipocytes from adipo-fibroblast progenitors. Here the authors are evaluating mRNA splicing in fully differentiated adipocytes. At most, the authors show that under some conditions and after extensive normalization, the ratio of unspliced to spliced mRNA is reduced in certain clusters of differentiated cells. Whether this splicing pattern has anything to do with adipocyte differentiation or function remains unclear.

1. Response: We thank the Reviewer for these helpful and insightful comments on our findings. We recognize that our claim of "delayed adipocyte differentiation (i.e., adipogenesis) rate" may not have been accurate enough. We have now changed it to "delayed adipocyte maturation rate" throughout the manuscript, including the title, to improve accuracy and better highlight our

findings in human VAT adipocytes. We still believe that our results in adipocytes are of importance given the significant overlap of the identified adipocyte maturation rate genes with adipogenesis genes that we have now added to the revised manuscript as follows below. Moreover, our revised results provide insight into how adipocyte maturation is related to the pathogenesis of MASLD.

We have now performed a differential gene expression analysis leveraging a previously published dataset of human visceral ASPCs differentiated to adipocytes from the omentum [PMID 38729152] to provide more insight into our adipocyte maturation rate gene set (Supplementary Table 2). We found a significant overlap (p=0.002) between our VAT adipocyte maturation rate gene set and differentially expressed VAT adipogenesis genes, resulting in 666 overlapping genes. Moreover, taking those 666 genes, we re-performed the analysis with scVelo [PMID 32747759] and found similar significant results as with the full adipogenesis maturation rate gene set (2,000 genes) (new Supplementary Figure 5). Accordingly, the individuals with MASLD have a lower latent time when compared to the individuals without MASLD ($p=4.9x10^{-31}$). This lower latent time was also observed in the individuals with MASH in comparison to the individuals without MASLD ($p=2.1x10^{-14}$). Furthermore, the 666 genes that overlapped between the differentially expressed genes by VAT ASPC differentiation and the adipocyte maturation rate gene set are enriched for pathways in morphogenesis, development, and activin receptor signaling (new Supplementary Figure 5 and new Supplementary Table 3). These new findings further support the role of delayed adipocyte maturation rate in MASLD progression. Finally, we also found that the 666 genes still explained a significant proportion of heritability in ALT and FLI (new Supplementary Figure 16), further emphasizing the finding that adipocyte maturation is under genetic regulation and contributes to MASLD associated traits.

We have now revised the manuscript to add these results to the revised Methods (page 12, lines 2-4, 12-23); page 13, lines 1-6; page 14, lines 17-20; page 18, lines 9-14), Results (page 22, lines 9-22; page 31, lines 2-5), new Supplementary Figures 5 and 16, and a new Supplementary Tables 2 and 3.

Point 2. The authors state that they obtained "highly expressed, specific marker genes for each subtype"…. and defined unique marker genes as being only present in one subtype." Presumably, the list of unique genes is presented in Suppl table 3. However, it is difficult to conceive that IRS1, PPARG, PPARA, STAT3, and ABHD5 are unique to hAd2 as seems to be claimed. If these are differentially expressed (not unique), please list the magnitude, % of cells expressing, etc.

2. Response: We thank the Reviewer for this helpful comment. To improve clarity, we have now renamed these as "marker genes" of the adipocyte subtypes instead of "unique marker genes". We previously defined them as "unique marker genes" because they are the only genes identified

in their respective subtype that passed a Bonferroni p-value of 0.05 and are expressed at a log2FC>0.25, similarly as in previous single cell RNA-seq studies [PMID 40196457, 33915568, 35296864, 37468583, 40258971]. They are generally expressed in all subtypes, but not to this same high degree. We have now clarified this in the revised Methods (page 11, lines 13-18). As suggested by the Reviewer, we have also now included the full output passing Bonferroni p-value of 0.05 from FindAllMarkers with the percentage of cells expressing these genes and their magnitudes for all our annotated adipocyte subtypes into the new Supplementary Table 4.

Point 3. Did the authors consider common genetic variants of MASLD that are expressed in adipocytes (PNPLA3, TM6SF2, MBOAT7) between the non-steatotic and MASLD patients? These may confound interpretation of scRNAseq data as variants in these genes are known to promote MASLD through reductions in VLDL TAG secretion in the liver and not necessarily defects due to adipose function.

3. Response: We thank their Reviewer for this insightful comment. We did not correct for the genotype status of the known *PNPLA3*, *TM6SF2*, and *MBOAT7* GWAS variants, rs738409, rs58542926, and rs641738, in our adipocyte maturation rate analysis of the 2,000 genes. As the *PNPLA3* gene is one of the 2,000 genes in this adipocyte maturation rate gene set used in the latent time analysis, we have now corrected for the genotype status of the *PNPLA3* variant rs738409. <u>We found that the revised results in VAT stay significant after this correction for both MASLD ($p=3.1x10^{-36}$) and MASH ($p=1.7x10^{-27}$) (new Supplementary Figure 4).</u> Furthermore, in SAT, upon adjusting for *PNPLA3* status, we also find the result to remain significant ($p=1.1x10^{-5}$) (new Supplementary Figure 9). We have now included these new results into the revised Methods (page 14, lines 5-7), Results (page 22, lines 1-6; page 26, lines 18-21), and new Supplementary Figures 4 and 9. The other two genes that Reviewer 2 is listing above, *TM6SF2* and *MBOAT7*, are not involved in determining the adipocyte maturation rate, i.e., they are not among the 2,000 genes. Therefore, we did not adjust our analysis for those.

Other concerns:

Other concern 1. • The authors state “Subtype hAd2 comprises pathways specific to lipid metabolism”; Table 3 lists >340 GO terms the vast majority of which have nothing to do with lipid metabolism. While it is true that the top 10 GO terms by fold-enrichment are involved in lipid metabolism, this enrichment is biased by the source (adipocytes), and the same genes are represented in seemingly contradictory pathways (GO:0010888 GO:0010889).

1. Response: We thank the Reviewer for careful attention to Supplementary Table 3 (now Supplementary Table 7). As these are subtypes of adipocytes, we used all genes expressed in adipocytes to define their subtype classifications and determine their functional specificity, in line with previous studies [PMID 35296864, 39856219]. Adipocytes are highly heterogeneous,

as previous studies have observed [PMID 34223880, 35296864, 39694039, 36922516, 33116305, 34380013, 31420514], and therefore, despite their general function for storing excess energy, they exhibit other distinct functions related to their microenvironment. The GO pathways appear to be contradictory; however, GO:0010889 is a more general pathway, e.g. regulation of triglyceride storage, and is defined by any process that regulates the rate or frequency of triglyceride storage. Therefore, it can be involved in both negative and positive regulation. GO:0010888 involves the negative regulation of lipid storage. As this hAd2 is a subtype that is highlighted by its involvement in lipid metabolism, it is still likely that it possesses the ability to negatively or positively regulate lipids, in line with the role of a basal adipocyte subtype.

Other concern 2. • How is blood platelet count a negative control? How is latent time calculated in cells that don't have a nucleus?

Response 2: We thank the Reviewer for this helpful comment. We would like to clarify that we employed blood platelet count as a phenotypic trait, not as single nucleus RNA-seq data set. Thus, we are not claiming to use any single nucleus RNA-seq data from platelets and we did not isolate blood for single cell analysis. Instead, we compared the latent time derived from adipocytes between individuals with a blood platelet count that was lower than the median blood platelet count and those with a blood platelet count above the median blood platelet count, which resulted in a non-significant p-value. We have now clarified this in the revised Methods (page 14, lines 9-10) and Results (page 22, line 6-7). We hope this clarifies the use of blood platelet count as a negative control trait.

Other concern 3. • What about other cell types in the snRNA set, particularly ASPCs.

Response 3: We thank the Reviewer for this helpful comment. We primarily focused on adipocytes due their known heterogeneity and key role in lipid storage [PMID 34223880]. In our analysis of VAT and SAT ASPCs, we did not find any clear differences in adipose stem and progenitor cells (ASPCs), i.e., we did not detect VAT or SAT ASPC subtype proportions to differ by the MASLD status or by sex (new Supplementary Figure 11). To further address Reviewer's comment, we have also now performed VAT and SAT ASPC differential expression (DE) analyses by the MASLD status and by sex.

In the VAT and SAT ASPC DE analyses, we found 261 DE genes by MASLD and 123 DE genes by sex in VAT (new Supplementary Table 8), and 158 DE genes by MASLD and 349 DE genes by sex in SAT (new Supplementary Table 9), respectively. To understand their biological processes, we also tested the identified ASPC MASLD and sex DE genes for functional pathway enrichments using WebGestalt [PMID 31114916].

In VAT, the upregulated DE genes by MASLD were enriched for example for nucleotide-sugar biosynthesis process, complement activation, and regulation of inflammatory responses (new Supplementary Figure 12 and Supplementary Table 10), underscoring a potentially pro-inflammatory microenvironment for adipogenesis.

In SAT, the upregulated genes by MASLD were overrepresented in responses to growth factors, while the downregulated genes by MASLD were enriched for positive regulation of fatty acids (new Supplementary Figure 12 and Supplementary Table 10). The combination of these pathways potentially indicates the inhibition of adipogenesis in SAT.

Among the VAT DE genes by sex, the upregulated genes in females were enriched for collagen processes and extracellular matrix organization, suggesting a greater drive toward adipocyte differentiation in females than in males (new Supplementary Figure 13, Supplementary Table 11). These were similar pathways as the ones seen among the upregulated genes in females for SAT (new Supplementary Figure 13, Supplementary Table 11). We have now included these VAT and SAT ASPC DE results into the revised Methods (page 11, line 2; page 17, lines 17-21), Results (page 27, line 23; page 28, lines 1-21), Discussion (page 37, lines 15-19), and new Supplementary Figures 11, 12, and 13, and Supplementary Tables 8, 9, 10, and 11, respectively.

In addition, we have conducted VAT ASPC *cis*-eQTL analysis to investigate their genetic regulation. We detected 71 eGenes, 19 of which (26.8%) overlap with the VAT adipocyte maturation rate gene set; 6 of which overlap with the VAT DE genes by sex; and 3 of which overlap with the VAT DE genes by MASLD, respectively. We show these VAT ASPC *cis*-eQTL results for the Reviewer here; however, after careful consideration, we chose not to include them into the revised manuscript because we recognize that larger VAT snRNA-seq cohorts are needed for a more powerful ASPC *cis*-eQTL analysis.

Other concern 4. • Show the Louvain clustering in figure 1d

Response 4: We apologize for our oversight and thank the Reviewer for this helpful comment. We have now included a new Supplementary Figure 2, showing the Louvain clustering (resolution=0.6) from Figure 1d.

Other concern 5. • The same non-steatotic controls seem to be used in multiple comparisons.

Response 5: We thank the Reviewer for this helpful comment. Unfortunately, we do not have additional sets of non-steatotic controls to compare our results to. Therefore, we are performing the analyses with the same non-steatotic controls. However, to clarify, we are not using the same controls between VAT and SAT. For VAT, there are 5 non-steatotic controls in MAFALDA 1

and 19 non-steatotic controls in MAFALDA 2, and for SAT, there are 44 non-steatotic controls in KOBS. The controls in MAFALDA 1 and MAFALDA 2 do not overlap. We are not aware of any previous human VAT or SAT snRNA-seq cohorts with multiple sets of controls when comparing cases and controls by a condition. For example, the following previous VAT and SAT snRNA-seq papers did not have multiple sets of human controls within their snRNA-seq datasets: PMID 35296864, 39558077, 39856219, 39694039.

Other concern 6. • What genes in Figure 2H, I are DE between subtypes?

Response 6: We thank the Reviewer for this constructive comment. We have now included a new supplementary table with the marker genes of the subtypes featured in Figure 2h and i (Supplementary Table 4). There are 252 marker genes in adipocyte subtype hAd1, 246 in hAd2, 506 in hAd3, 141 in hAd6, and 195 in hAd7, respectively. Furthermore, we have also included the genes that are differentially expressed across subtypes that are part of the adipocyte maturation rate gene set (new Supplementary Table 5). Of these, 144 were marker genes for adipocyte subtype hAd1, 143 in hAd2, 150 in hAd3, 46 in hAd6, and 40 in hAd7, respectively. In addition, we have revised the Results section (page 24, line 11-17) to include these new results.

Other concern 7. • The meaning of figure 2J is unclear.

Response 7: We thank the Reviewer for this helpful comment. We would like to clarify that figure 2j was used to emphasize that certain groupings of cells, determined by the nearest neighbor graph, are correlated with specific phenotypic traits. We used it as an additional tool to check if there are abundant cellular neighborhoods correlated with MASH. The global p-value is used to test whether the null hypothesis (i.e., that there is no association between the neighborhood and the phenotypic trait) can be rejected or not. The phenotypic trait of interest in the cohort was permuted to obtain a null distribution for this analysis. Based upon the permuted p-value of 0.01, the results show that there is an association between groups of cells and MASH. We have now revised the Methods (page 15, lines 18-21) and Results (page 24, lines 4-9) to better explain and clarify this.

Other concern 8. • The calculation of subtype proportion does not map onto figure 2g. Please provide the list of raw and inverse-normalized subtype proportions.

Response 8. We thank the Reviewer for this helpful comment. We would like to point out that using the inverse-normalized subtype proportions, the data in fact does map fully onto figure 2g. To verify that for the Reviewer, we have now included a table below with the raw and the inverse-normalized subtype proportions for figure 2g.

Table for Reviewer 2, showing the list of raw and inverse-normalized subtype proportions of Figure 2g.

	MASH		Non-steatotic liver	
Adipocyte subtype	Raw proportions	Normalized proportions	Raw proportions	Normalized proportions
hAd1	0.714, 0.727, 0.330, 0.639, 0.348, 0.534, 0.706, 0.500, 0.849, 0.460, 0.114, 0.107, 0.500, 0.341, 0.214, 0.373, 0.609, 0.359, 0.268, 0.063, 0.256, 0.813, 0.159, 0.694, 0.336, 0.750	2.287, 1.164, -0.225, 0.803, 0.112, -0.168, 0.657, 0.341, 1.282, 1.834, -0.461, -0.524, 0.282, 0.225, -0.341, 0.524, 1.593, 0.589, -0.657, -0.882, -0.400, 1.420, -1.420, 0.461, 0, 0.728	0.273, 0.017, 0.186, 0.348, 0.471, 0.068, 0.605, 0.038, 0.039, 0.337, 0.710, 0.067, 0.256, 0.118, 0, 0.500, 0.222, 0.160, 0.442	-0.056, -1.282, -1.593, -0.112, 0.967, -0.803, 0.882, -1.061, -0.728, 0.168, 0.400, -2.287, -0.967, -0.589, -1.164, 1.061, -0.282, -1.834, 0.056
hAd2	0.143, 0.006, 0.085, 0.014, 0.004, 0.017, 0.013, 0.031, 0.014, 0.004, 0.053, 0, 0.143, 0.015, 0.349, 0.060, 0.085, 0.333, 0, 0.625, 0.011, 0, 0.076, 0.012, 0.109, 0.063	-1.164, -1.593, -0.400, 0, -1.061, 0.341, -0.524, -0.168, -0.728, -1.834, -0.803, -1.420, 1.061, -0.967, -0.112, -0.056, -0.657, 0.728, -0.225, 1.282, -1.282, -0.882, 0.657, 0.400, 0.524, 0.282	0.182, 0.915, 0.421, 0.240, 0, 0.739, 0.013, 0.087, 0.731, 0.066, 0.013, 0.363, 0.239, 0.030, 0.857, 0.111, 0.129, 0.017, 0.078	-0.589, 1.834, 1.164, -0.461, -2.287, 1.420, -0.341, 0.225, 1.593, 0.168, 0.589, 0.967, 0.803, -0.282, 2.287, 0.112, 0.882, 0.461, 0.056
hAd3	0.143, 0.042, 0.392, 0.038, 0.033, 0.017, 0.196, 0.250, 0.014, 0.106, 0.735, 0.143, 0.095, 0.473, 0.230, 0.209, 0.110, 0.218, 0.561, 0.063, 0.533, 0, 0.378, 0.118, 0.316, 0.063	0.461, -0.282, 1.164, -0.341, -0.803, -0.657, 0.803, -0.168, -0.589, 0, 2.287, -0.112, -1.834, 1.061, 0.728, -0.728, -0.461, 0.112, 1.593, -1.420, 1.420, -0.882, 0.882, 0.524, 0.225, -0.056	0.091, 0, 0.025, 0.073, 0.176, 0.010, 0, 0.803, 0, 0.245, 0.103, 0.380, 0.106, 0.003, 0, 0.111, 0.040, 0.087, 0.091	0.056, -0.967, 0.657, 0.168, 0.400, -0.524, -1.061, 1.834, -1.164, 0.967, 0.341, 1.282, -0.225, -1.282, -1.593, -0.400, -2.287, 0.589, 0.282
hAd6	0, 0.224, 0.193, 0.293, 0.615, 0.431, 0.085, 0.219, 0.123, 0.431, 0.091, 0.750, 0.262, 0.170, 0.195, 0.343, 0.196, 0.058, 0.146, 0.250, 0.189, 0.188, 0.382, 0.176, 0.233, 0.125	-1.164, 0.282, -0.461, -0.282, 1.420, 1.164, -0.882, 0.400, -0.524, -0.168, -2.287, 1.834, -0.341, 0.112, 0.657, 0.589, -0.967, -1.282, -1.420, 0.524, 0, -0.225, 1.061, -0.589, 0.168, -0.657	0.455, 0.068, 0.361, 0.334, 0.353, 0.181, 0.382, 0.067, 0.228, 0.347, 0.168, 0.187, 0.389, 0.842, 0.143, 0.278, 0.609, 0.734, 0.377	0.882, -0.112, 0.225, 0.803, 0.728, -0.056, 0.341, -1.593, 0.056, -0.728, -0.803, -0.400, 1.282, 2.287, -1.834, -1.061, 0.967, 1.593, 0.461
hAd7	0, 0, 0, 0.014, 0, 0, 0, 0, 0, 0, 0.008, 0, 0, 0, 0.013, 0.015, 0, 0.032, 0.024, 0, 0.011, 0, 0.004, 0, 0.006, 0	-0.589, 0, -0.524, 2.287, -1.061, -0.803, -0.657, 0.341, -0.282, 0.112, 0.803, -0.225, -0.056, -0.341, 0.728, 0.589, -0.967, 1.834, 1.593, -1.164, 1.282, 0.056, 0.400, -1.420, 0.657, -1.282	0, 0, 0.007, 0.003, 0, 0.003, 0, 0.005, 0.002, 0.005, 0.006, 0.003, 0.010, 0.007, 0, 0, 0, 0.003, 0.013	-0.112, -0.461, 1.420, 0.524, -1.593, 0.225, -0.882, 1.164, -0.400, 0.282, 0.461, 0.168, 0.967, 0.882, -0.168, -0.728, -1.834, -2.287, 1.061

Other concern 9. • How were cells clustered in figure 1 and does this clustering agree with clustering in figure 2?

Response 9. We thank the Reviewer for this insightful comment. Due to the small sample size of the pilot dataset, MAFALDA 1 (n=11), we did not perform the same annotation on MAFALDA 1. However, to showcase the potential presence of all adipocyte subtypes, we took the marker genes of the subtypes from MAFALDA 2 and calculated their module scores using Seurat [PMID 31178118], which highlights the presence of these subtypes in MAFALDA 1 as well based upon gene expression in this smaller cohort (Supplementary Figure 6). We have now included these results into the revised Methods (page 12, lines 6-10) and Results (page 23, lines 6-10) and the new Supplementary Figure 6.

Other concern 10. • The manuscript could use editing from a native English speaker. For example the first sentence in the abstract and run on statement (pg 23 lines 21) is ambiguous.

Response 10. We thank the Reviewer for this constructive comment. We have now corrected the manuscript throughout for any grammatical errors, including the first sentence in the abstract and the run on statement that the Reviewer mentioned (page 31, lines 11-15).

Other concern 11. • How is a log2FC>0.25 for DEs considered biologically relevant?

Response 11. We thank the Reviewer for this helpful comment. This cut-point of $\log_2$FC>0.25 is on logarithmic scale, and thus indicates a 1.2-fold change. Within human transcriptomics data, this is considered a significant difference in expression, and it has commonly been used to determine upregulated and downregulated DE genes in multiple previous single cell papers [PMID 37845689, 39578645, 38862487, 33915568, 39694039, 40316535, 40044687]. Furthermore, for example, in a previous paper [PMID 39578645] that compared gene expression using brain snRNA-seq data between individuals with and without Alzheimer's disease, while employing an absolute $\log_2$FC>0.25, the DE genes had a mean absolute $\log_2$FC of 0.38 and SD of 0.14. This mean absolute $\log_2$FC is in fact even lower than the mean (0.61) absolute $\log_2$FC (SD 0.40) of the DE genes in adipocytes in our study.

In addition, the cut-point of $\log_2$FC>0.25 is employed in the FindAllMarkers function of the commonly used single cell tool, Seurat [PMID 31178118], to identify differentially expressed (DE) marker genes in one cell-type when compared to other cell-types [PMID 31178118, 34062119]. Moreover, we and others have shown that the cell-type marker genes defined to be DE using the cut-point of $\log_2$FC>0.25 show cell-type-specific functional pathway enrichments [PMID 37409528, 39515300, 35581624, 35296864, 39856219, 34857782], thus testifying for the biological relevance of this cut-point. This is also in line with the functional pathway

enrichments of the marker genes that we found using this cut-point in our study (Figure 2k, Figure 2l, now Supplementary Table 6, now Supplementary Table 7), further implying biological relevance of the genes determined using the cut-point of $log_2FC>0.25$. Furthermore, we also found functional pathway enrichments of the MASLD and sex DE genes in ASPCs using this cut-point, further implying biological relevance of the DE genes determined employing the cut-point of $log_2FC>0.25$ (Supplementary Figure 12, Supplementary Table 10, Supplementary Figure 13, Supplementary Table 11). Again, our results are similar with the functional pathway enrichments of the marker genes defined to be DE this way in the previous papers as well [PMID 39515300, 37845689, 39578645, 33915568, 39694039]. Taken together, we consider that $log_2FC>0.25$ for DEs has been shown to be biologically relevant.

Reviewer 1:
Response: We thank Reviewer 1 for the favorable comments.

Reviewer 2:
We thank the Reviewer for the helpful comments and suggestions on the manuscript and have addressed all questions that were raised. We hope that the revisions satisfactorily respond to all of the Reviewer's questions. On behalf of the authors, we feel that the revised manuscript is significantly strengthened based on these astute comments. The numbering of the revised pages we provide below refer to the tracked changes version of the revised manuscript. We also provide a clean manuscript. In our responses, we refer to previous papers using their PubMed IDs.

Reviewer #2 (Remarks to the Author):
The authors show that adipocytes of 'non-steatotic' controls have a different splicing pattern ('latent time) than subjects with steatosis, and that this phenomenon has a significant heritable component. This pattern is consistent with the widely held view that defective adipocyte function contributes to hepatosteatosis. The data are interesting and could advance the field.

However, several key problems remain in this revision:
1) Direction of causality. It is unclear whether one would find the same pattern of RNA splicing (latent time) in hepatocytes or other metabolic tissues.

Response to point 1: We thank the Reviewer for this constructive comment. We have now used scVelo (PMID: 32747759) to obtain the latent time of hepatocytes from a previously published MASLD case/control cohort with liver histology assessments (PMID: 39522884). In our latent time analysis of the hepatocytes, we found that the individuals with MASLD and MASH had a significantly higher latent time compared to the controls with non-steatotic liver ($p=2x10^{-82}$ for MASLD and $p=1.8x10^{-7}$ for MASH, respectively) (new Supplementary Figure 6). Next, we investigated to which zonation the hepatocytes with a higher latent time correspond. The rationale for this investigation was that in hepatocytes, previous papers have found that hepatocytes exhibit distinct functions based upon zonation (PMID: 39747812, PMID: 39847812, PMID: 30348985, PMID: 38199298). In this analysis, we found that the hepatocytes with the higher latent time coincided with zone 3 (Supplementary Figure 7), in line with a previous study that also observed the same finding while using pseudotime analysis (PMID: 38182825). Zone 3 is known to be involved in lipogenesis, while the other zones, such as zone 1 are involved in beta-oxidation (PMID: 39747812). Thus, individuals with MASLD and MASH have **a higher latent time in their hepatocytes, which resemble zone 3 hepatocytes involved in lipogenesis. The latent time analysis in VAT adipocytes in turn demonstrated that the individuals with MASLD and MASH**

have a lower adipocyte latent time than the controls with non-steatotic livers. This suggests that the VAT adipocytes from individuals with MASLD and MASH may be dysfunctional in their capacity to properly store fat, therefore promoting the ectopic fat accumulation, i.e., increased lipogenesis in the liver. Thus, the VAT latent time results of the individuals with MASLD and MASH may reflect the inadequate lipogenesis of their VAT adipocytes. We have now added these liver hepatocyte latent time results to the revised Methods (page 12, lines 270-281; page 13, lines 283, 287-289; pages 14, lines 310, 313-314, 322, 328; page 15, lines 329, 348-350) and Results (page 24, lines 515-524) as well as discussed them in the revised Discussion (page 36, lines 799-807).

2) Figure 5 implies that hepatosteatosis causes alterations in adipocyte gene expression (e.g., downregulated by MASLD). The point of the paper is the reverse causation, the validity of which is uncertain.

Response to point 2: We thank the Reviewer for the opportunity to clarify Figure 5. We disagree that Figure 5 would imply that hepatosteatosis causes alterations in adipocyte gene expression because differential expression (DE) analysis, regardless of whether it shows up- or downregulation by MASLD/MASH, cannot show a causal direction, and thus there is no causal direction in Figure 5c-f. Furthermore, Figure 5b in fact shows that regional variants in the adipocyte maturation rate gene sets are enriched for heritability of MASLD traits in the large UK Biobank, an independent cohort from the MAFALDA cohorts, indicating that genetic variants in these VAT adipocyte maturation rate genes contribute to MASLD traits, most likely through changing the adipocyte expression of these genes. Thus, the heritability results actually support the genetic role of the adipocyte maturation rate gene set in MASLD traits, not the other way round (i.e., reverse causal). Figures 5e-f also show that many of the DE genes by MASLD and MASH and most of the genes that are both DE by MASLD or MASH and regulated by adipocyte *cis*-eQTL variants (eGenes) are part of the adipocyte maturation gene set, which along with the heritability results in Figure 5b further supports their role in MASLD pathogenesis through adipocyte dysfunction. We have also now revised Figure 5e-f figure legends and the Results (page 32, lines 711-712; page 34, lines 746-747, lines 759-761) to more clearly indicate that we do show the DE genes by MASLD and MASH that are also eGenes (i.e., their adipocyte expression is *cis* regulated by their regional genetic variants).

To provide further support for adipocyte maturation impacting the development of MASLD, we have also now performed a Mendelian Randomization (MR) analysis using MR-Eggers tool (PMID: 28527048) and inverse variance weighting (IVW) method (PMID: 24114802) through TwoSampleMR (PMID: 29846171). In this MR analysis, we tested all independent (R^2=0.001) *cis*-eQTL variants that we identified to regulate adipocyte expression of adipocyte maturation genes (n=114 variants shown in Supplementary Table 21) as the

instrumental variables (IVs) to assess the MASLD outcome in the large UK Biobank, independent from the MAFALDA cohorts. Outcomes included the following MASLD traits: ALT, FLI, and PDFF and MASLD (PMID: 35047847). At nominal significance ($p<0.05$), we found between 11 to 17 SNPs (depending on the investigated MASLD trait), showing a putative causal path from the adipocyte maturation gene to the MASLD trait. Correcting for multiple testing (Bonferroni $p<0.05$), 3 adipocyte *cis*-eQTL SNPs were implicated for putative causality with the MASLD outcome. These include rs10889098 regulating VAT adipocyte expression of the *DAB1* gene, rs7464506 regulating the *LONRF1* gene, and rs7681503 regulating the *RN150* gene from the adipocyte maturation gene set, respectively. Even though these initial MR results further support the putative causality of these VAT adipocyte genes in MASLD, we recognize that future larger VAT snRNA-seq cohorts are needed to identify all *cis*-regulatory variants of the adipocyte maturation genes for additional MR testing. Thus, as larger study samples are needed to more comprehensively identify MR signals among the adipocyte maturation gene set, we prefer to include these initial MR results only for the Reviewer.

DAB1 interacts with members of the LDL receptor family in the brain (PMID: 10380922, PMID: 10827173); however, little is known about its behavior in adipocytes. Given the interaction with the LDL receptor family and its presence in the adipocyte maturation gene set, *DAB1* may be involved in lipid metabolism of adipocytes. *LONRF1* has previously been identified as a marker gene in adipocytes from subcutaneous adipose tissue (SAT) (PMID: 38991381). Within VAT adipocytes, we also identified it as an adipocyte marker gene. Furthermore, *LONRF1* has been suggested to be linked to tissue remodeling and wound healing (PMID: 36888978). Its presence among the adipose maturation rate gene set and the putative causal relationship of the *LONRF1 cis*-eQTL variant in MASLD traits provide support for its genetic effect on adipose tissue remodeling and its impact on MASLD pathogenesis. *RNF150* has been attributed to the release of free fatty acids in cell lines (PMID: 22384002), which may contribute to the balance of free fatty acids going to the liver versus being stored by adipocytes. In our study, adipocyte expression of *RNF150* is also significantly upregulated in individuals with MASLD and MASH (Supplementary Table 20), which along with the MR result further suggests its potential role in impaired adipocyte maturation, contributing to the development of MASLD.

3) The original review raised the central concern that the concept of 'latent time' as it relates to adipocyte biology has no experimental validation or coherent biological basis. Relabeling 'adipogenesis' as "adipocyte maturation" does not address this conceptual flaw. The introductory statement: "We hypothesized that impaired VAT adipocyte maturation impacts metabolic dysfunction associated steatotic liver disease (MASLD)." is circular reasoning. It should be noted that the computational method for latent time was used to address splicing ratios in a very short time frame

(hours/days). It is unclear what "adipocyte maturation' means when the temporal sampling domain is essentially random, and the lifespan of a human adipocyte is many months, if not years.

Response to point 3: We thank the Reviewer for these helpful comments. Previous studies have highlighted the biological basis of pseudotime and latent time in adipocyte biology (PMID: 35882069, PMID: 37337126, PMID: 37398933, PMID: 37716088, PMID: 40196457, PMID: 38729152), including the experimental adipogenesis data (PMID: 38729152) that we used to perform our overlap assessment of adipocyte maturation genes with experimentally generated adipogenesis gene set in our first revised version of this manuscript. Thus, this significant overlap between the VAT adipogenesis genes, generated in the previous VAT adipogenesis experiment (PMID: 38729152), and our VAT adipocyte maturation gene set validates the fact that our gene list contains 666 important VAT adipogenesis genes (see Results, page 23, lines 500-513 for details of the overlap).

We would also like to clarify that with our hypothesis that "impaired VAT adipocyte maturation impacts MASLD" we meant that impaired VAT adipocyte maturation contributes to the development of MASLD in the liver. Therefore, the inability of VAT adipocytes to become fully matured adipocytes, contributes to the accumulation of ectopic fat in the liver, inducing MASLD. We do not consider this hypothesis to be circular given that our heritability (Figure 5b) and MR results (see above) suggest a putative causal path from VAT to liver and not the other way around (i.e., reverse causal). However, we recognize that showing causality requires further experimental work, e.g., animal studies, and we have now discussed this limitation in the revised Discussion (page 39, lines 877-880; page 40, lines 881-883) as well as revised the Abstract (page 2, lines 43, 55) and the Results (pages 21, lines 450-454) and Discussion sections (pages 35, line 784; page 37, line 812; page 40, line 896) to clarify our hypothesis and not to overstate the putative causal direction.

Please note that very relevant to our study, a recent Nature Genetics paper published this year (PMID: 39856219) used SAT and VAT single nucleus RNA-seq data and immunofluorescence to identify and validate "classical adipocytes" and "nonclassical adipocytes". Then employing pseudotime analysis, the authors also identified "nonclassical adipocytes" as an intermediate stage between progenitor cells and the "classical adipocytes", which have not reached full maturity yet (PMID: 39856219). **In line with that paper, we implicate the VAT adipocytes with delayed maturation in MASLD and MASH.**

We would also like to clarify that we are not only taking cells at random, but also from tens of individuals in our largest to date VAT snRNA-seq cohort (total n=74 individuals) to capture cells at different moments from many people in a human lifespan. The very idea that

snRNA-seq is random allows us to capture cells at any stage of maturation as each cell has an equal chance of being captured (PMID: 26544934). Therefore, our results are not necessarily limited to hours/days. We are taking adipocytes from 74 individuals to showcase the heterogeneous spectrum of an adipocyte maturation cycle, which highlighted a potential mechanism of MASLD pathogenesis through VAT adipocyte dysfunction. Noteworthy, our results are also in line with previous papers, showing that adipocytes are highly heterogeneous, with sex (PMID: 32967914, PMID: 32418585, PMID: 36367881, PMID: 36624843, PMID: 30544158) and obesity (PMID: 38086922, PMID: 19017614) now known to contribute to their heterogeneity. Furthermore, the concept of comparing different stages of maturation to infer how a disease state might develop has also been used in previous papers (PMID: 33907320, PMID: 36968070, PMID: 37095364), including one paper about immune responses in COVID-19 that compared the unspliced ratio in different subpopulations of monocytes between COVID-19 cases and healthy controls (PMID: 37095364). Importantly, these previously published papers have shown the validity of using pseudotime and splicing to better understand differentiation and disease development, similar to what we have presented in our manuscript.

4) The latent time analysis depends heavily on a limited number of control comparisons, and the methods section is unclear as to the source and phenotypes of the non-steatotic controls, and when and how these control samples were obtained and processed. Were the controls part of consecutive bariatric surgeries that were obese, but not steatotic, as implied? The authors must explicitly detail how and when the non-steatotic controls were obtained and processed for sn-RNAseq since all of the comparisons are based on this relatively small set of controls. A reader can't extract the relevant information from references 24,25. It is unclear how the numbers in table 1 align with the description in the methods.

Response to point 4: We thank the Reviewer for these helpful comments. We would first like to clarify that the controls and MASLD and MASH cases were all individuals with obesity undergoing bariatric surgery. **Please also note that the non-steatotic controls are individuals with obesity without MASH or MASLD, and that the MASH cases are included in the number of the MASLD cases. Thus, in our study, all individuals with MASH are already included in the MASLD group. Accordingly, in MAFALDA 1, there are 6 cases with MASLD, 4 of whom also have MASH, whereas the 5 controls have neither MASLD nor MASH, resulting in a total of 11 individuals. In MAFALDA 2, there are 44 cases with MASLD, 26 of whom also have MASH, whereas the 19 controls have neither MASLD nor MASH, resulting in a total of 63 individuals. We have now fully revised Supplementary Table 1 to clearly show the numbers of individuals in the three groups: MASLD, MASH, and controls.** Finally, all samples were sequenced together to avoid sequencing batches. We have now clarified the numbers of cases and

controls as well as the collection of the samples and processing in the revised Methods section (page 5, lines 105, 109-110, 115-121).

5) What is meant by replication? According to the text and table 1 MAFALDA1 has 11 subjects (10 with steatosis). It is not obvious what controls were used (see comment 4). What specific genes were replicated? Or was it the phenomenon of splicing ratios, without reference to specific genes?

Response to point 5: We thank the Reviewer for these very helpful comments. As described in detail above, we would like to first clarify that **in MAFALDA 1 there are 11 subjects, of whom 6 have MASLD and 5 have non-steatotic livers. In our study, all individuals with MASH are already included in the MASLD group, and thus we have more controls than the Reviewer thought (please also see revised Supplementary Table 1 that is now fully clarified to indicate the numbers of individuals in the three groups: MASLD, MASH, and controls).** Regarding replication, we tested the finding that cases with MASLD and MASH have a lower latent time than the controls with non-steatotic livers for replication. So, no individual genes were replicated, rather we assessed the significance and the direction of effect of the latent time for replication as follows: First, we observed a lower latent time in the cases with MASLD and MASH than the controls with non-steatotic livers in MAFALDA 1 and then we tested this result for replication in MAFALDA 2, while considering $p<0.05$ and the same direction of effect as a replicated result. Using this standard replication procedure, the latent time results were replicated (Figure 2c, 2d). Please also note that MAFALDA 1 and MAFALDA 2 comprise independent individuals with no overlap. We have now clarified the replications in the revised Results (page 22, lines 488-490). As explained in our response to Reviewer's comment 4, we have also now revised Supplementary Table 1 to clearly show the numbers of cases and controls and revised the Methods section (page 5, lines 105, 109-110, 115-121).

Reviewer #4 (Remarks to the Author):
Response: We thank the Reviewer for the helpful comments and suggestions on the manuscript and have addressed all questions that were raised. We hope that the revisions satisfactorily respond to all of the Reviewer's questions. On behalf of the authors, we feel that the revised manuscript is significantly improved with this round of revision. The numbering of the revised pages we provide below refers to the tracked changes version of the revised manuscript. We also provide a clean manuscript. In our responses, we refer to previous papers using their PubMed IDs.

Gelev et al report on single-nucleus RNA seq from SQ and visceral adipose tissue, and report that gene expression profiles differ in those individuals with MASLD vs. those without. Moreover, individuals with liver disease are more likely to have gene expression patterns suggesting less differentiated adipocytes based on trajectory analysis. The strengths of the manuscript include the 3 distinct cohorts, integration of liver metabolic data from the adipose donors, the robust analysis of gene expression using snRNA-seq, and the integration of DNA sequencing for eQTL analysis. Weaknesses of the manuscript include the lengthy methods section, which is laborious to read and distracts from the results section – it could be substantially shortened and/or some of the methods moved to supplementary files. The authors do not provide information about the metabolic characterization of the biopsy donors beyond age, BMI and presence or absence of MASLD and DM. It would be important to assess relationships between the findings to key clinical variables such as age, BMI and glycemic control because these phenotypic variables could also contribute to both MASLD and the adipose gene expression phenotypes, a key analysis when trying to assess direction of causality. A major concern is that the authors indicate conclusions of causality in many sections of the manuscript, when their cross sectional analysis cannot. This point was raised by reviewer 2 but incompletely addressed.
The authors have been somewhat responsive to the prior critiques, adding the trajectory analysis to the liver samples – finding that the pattern does not mirror that of the adipocytes. Moreover, some of the points in the response to review have not been adequately added to the manuscript itself.

We thank the Reviewer for these insightful and helpful comments.

First, as suggested by the Reviewer, we have now shortened the Methods and moved parts of the Methods to **Supplementary Note**. We believe that the revisions helped improve readability.

Second, we have now performed a logistic regression analysis to investigate the relationships between the latent time, MASLD, and the suggested additional clinical variables (please see **Table A** below, **Supplementary Table 7**). We found that after multiple test correction, only insulin (corrected for the diabetes status) remained significantly associated with MASLD.

Considering insulin was the only clinical feature associated with MASLD, we then ran a linear regression analysis to assess the relationship between latent time and insulin. We found that insulin was inversely associated with the latent time (beta=-0.051, p-value=$2.67x10^{-8}$), i.e., higher insulin level is associated with a lower latent time, in line with the MASLD and MASH findings. This indicates that insulin levels also associate with the latent time. We have now included this finding in the revised Methods (page 21, lines 446-454) and Results (page 29, lines 615-623).

Table A. Insulin is the only clinical feature associated with MASLD after multiple testing correction.

Predictor	Beta	SE	Z	P	Padj
**Age (years)**	0.032	0.033	0.974	0.330	1.000
**BMI (kg/m^2)**	0.057	0.083	0.683	0.494	1.000
**Glucose (mg/dL) (adjusted for DM status)**	0.680	0.330	2.061	0.039	0.354
**Insulin (uUI/mL) (adjusted for DM status)**	1.481	0.481	3.077	0.002	0.019
**HbA1c (mmol/mol)**	0.030	0.044	0.692	0.489	1.000
**Cholesterol (mgd/L)**	-0.011	0.011	-1.007	0.314	1.000
**HDL-C (mg/dL)**	-0.043	0.030	-1.463	0.143	1.000
**LDL-C (mg/dL)**	-0.011	0.011	-1.037	0.300	1.000
**TG (mg/dL)**	0.017	0.007	2.294	0.022	0.196

Table A footnote: A logistic regression analysis was performed, in which the MASLD status was the outcome. SE indicates standard error; Z, z-value; P, p-value using the Wald test; Padj, Bonferroni [illegible]

Third, we recognize that our results do not show causality and accordingly, and we have carefully revised the entire manuscript to avoid any causality claims, including rephrasing “adipocyte maturation” to “predicted degree of adipocyte differentiation”, as suggested by the Reviewer in major comment 2.

Major comments:

1. Within the text and figures, it is not very clear that the MASH group is a subset of the MASLD group. This should be clearly spelled out very early in the results section.

Response to major comment 1: We thank the Reviewer for this helpful comment. As suggested by the Reviewer, we have now revised the early part of the Results (page 27, line 584; page 28, lines 585-588) and the figure legends to clearly spell that the MASH group is a subset of the MASLD group.

2. The authors use the term “adipocyte maturation rate” – this is misleading. There is no time-dependent variable in this cross-sectional analysis. Likewise, latent time is not clear in this context. A different label would be more precise. As pointed out by the prior reviewer 2, the trajectory analysis is predicting degree of differentiation. In the case of adipocytes, it does not mean that this is a currently ongoing process, just that the adipocytes are in different states at the time of the biopsy sampling. The authors should remove language suggesting this is a dynamic ongoing process throughout the manuscript.

Response to major comment 2: We thank the Reviewer for this constructive comment. We recognize the issue related to the term “adipocyte maturation rate” and have now thoroughly revised the entire manuscript to remove this wording and use “predicted degree of adipocyte differentiation” instead to better describe our findings and avoid misleading wording. We also modified the title. In addition, we clarified both in the Methods (page 20, lines 434-435) and Results (page 41, lines 889-891; page 47, lines 1012-1015) that with the adipocyte latent time gene set we mean the predicted degree of adipocyte differentiation gene set.

3. Differences in “adipocyte maturation rate” between non-steatotic and MASLD (box plots of Figures 1-4 (e.g. 1e, 2c, 3b, 4d, 4e, etc.)) are significant due to the large number of nuclei being assessed, but the actual magnitude of difference appears to be small but unclear. Can the authors provide this information e.g. provide difference in units corresponding to the actual trajectory analysis (which is scaled from 0 to 1)? Can the authors graph latent time data for each individual within the group?

Response to major comment 3: We thank the Reviewer for this helpful comment. As suggested by the Reviewer, we have now adjusted all pertinent **Figures (1e, 2c, 3b, 4d, 4e, etc.)** to match the initial scaling of 0 to 1, while adjusting for the covariates (please see page 20 of the Methods and **Supplementary Note 1.7**). We also now include new **Supplementary Tables 3-6, 8-10, 13, 14, 16, 17, 21, 22, 29-35** with summary statistics for each group of the latent time results, corresponding to their respective latent time in **Figures 1e, 2c, 3b, 4d, 4e, etc**. For the Reviewer, we also graph the latent time data for each individual within the groupings in MAFALDA 1, MAFALDA 2, and KOBS cohorts below.

Figure 1. Graphing each individual's latent time data in the MASLD phenotype groups for the three used cohorts. Boxplots show individuals' latent time (adjusted for covariates) per cohort. (**a**) MAFALDA 1 (top) and (**b**) MAFALDA 2 (middle) show individuals latent time as boxplots, colored by non-steatotic liver, MASLD, or MASH. (**c**) KOBS (bottom) shows individuals' latent time as boxplots, colored by non-steatotic liver or MASLD.

a

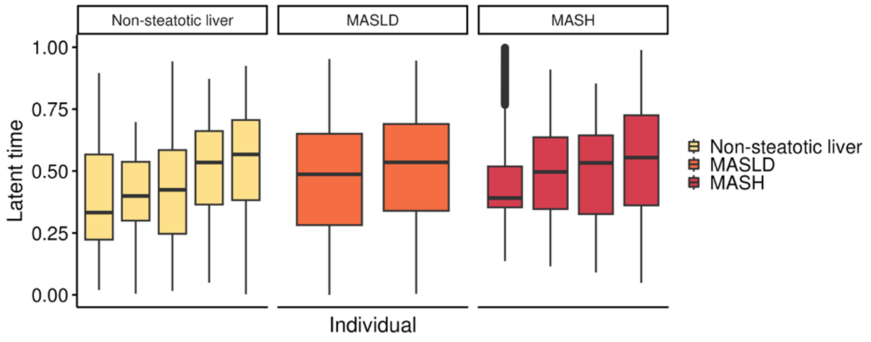

b

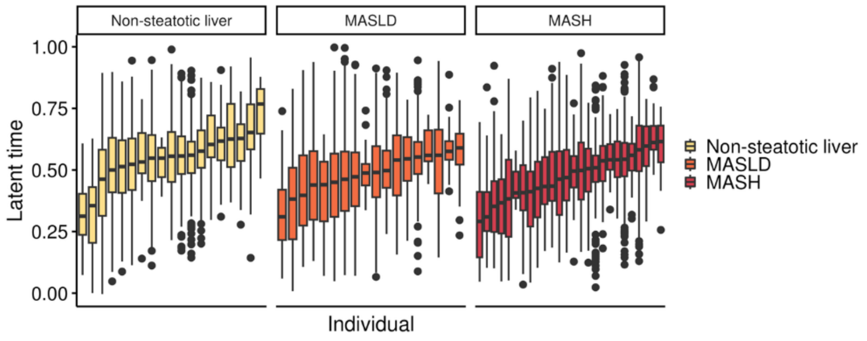

c

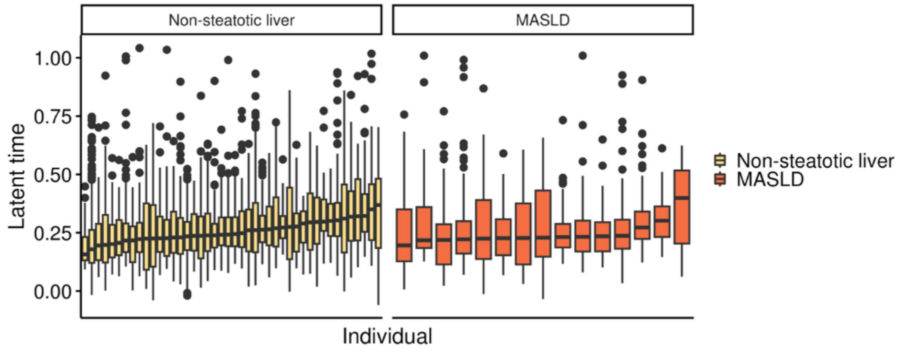

4. The authors indicate (line 548) that the overall reduction in latent time in VAT adipocytes is driven by the hAd2 subtype. I don't think the authors have provided evidence for this – it remains a hypothesis and association. Similarly, the authors conclude that sex is driving the heterogeneity of VAT adipocytes (line 593) and contributes to VAT maturation and function(line 596) but don't provide sufficient evidence to support these statements beyond association. Likewise, line 635 indicates sex has a profound effect on rate of adipocyte maturation in SAT – association, not cause-effect relationship. Please modify these conclusions.

Response to major comment 4: We thank the Reviewer for this constructive comment. We recognize that our data do not provide evidence beyond associations, and accordingly we have adjusted the entire manuscript throughout to avoid overstatements and replaced all previous wording implying causality, such as derivatives of "drive" and other phrasing that implies causality to wording that does not imply causality such as "associated" and "linked".

5. Can the authors clarify what percentage of adipocyte maturation genes which are differentially expressed in donors with MASLD are also expressed in hepatocytes, and are these differentially expressed in MASLD as well? This is important to interpret the results of the eQTL analysis and assessment of heritability as well.

Response to major comment 5: We thank the Reviewer for this helpful comment. Among all expressed genes in hepatocytes (27,200 genes), 143 genes overlap with the 169 adipocyte DE genes by MASLD that are also part of the adipocyte latent time gene set. Among these 143 overlapping genes (143/169; 84.6%), there are 46 genes that are also DE in hepatocytes (46/169; 27.2%). However, only 25 of these 46 DE genes match directionally (25/169; 14.8%) (i.e., the gene is upregulated both in adipocytes and hepatocytes or downregulated in both cell-types) (**Supplementary Table 18**). These relatively small overlaps of the DE genes in the same direction in both adipocytes and hepatocytes support and are in line with the different direction of the latent time by MASLD observed in adipocytes versus hepatocytes. We have now added these new comparisons to the Results (page 44, lines 959-967). Details on the DE by MASLD analysis in hepatocytes can be found in **Supplementary Note 1.13**.

6. The authors note (line 616) that VAT adipocyte proportions change with progression of MASLD into MASH. The cross-sectional data do not allow this conclusion. Please revise the description of these results throughout. Association does not mean causation.

Response to major comment 6: We thank the Reviewer for this helpful comment. We recognize that this is an overstatement and accordingly, we have revised the entire manuscript throughout to avoid any causation claims. Please also see our response to major point 4.

Minor comments:
- Why was the MT threshold was set to >10%? Typically, single-cell studies use MT>10%, but most single-nucleus studies set it >5% or even >1%.

Response to minor comment 1: We thank the Reviewer for this helpful comment. We would like to clarify that we chose the 10% cutoff to follow the same MT threshold as used in the previous adipose atlas paper by Emont et al., published in Nature [PMID: 35296864] that we used as reference for annotating cell-types and subtypes in our study. We have now included this citation for MT% in the manuscript to provide a rationale for using this cut point. We also now included new **Supplementary Figures 2, 3,** and **11** that clearly show comparable mitochondrial percentage across the VAT and SAT cell-types of the MAFALDA 1, MAFALDA 2, and KOBS cohorts when compared to the mitochondrial percentage across the VAT and SAT cell-types of this previous adipose atlas [PMID: 35296864]. We also found several previous papers that performed single nucleus RNA-sequencing instead of single cell RNA-sequencing that used this same MT 10% threshold [PMID: 38741048, PMID: 39515300, PMID: 38087243].

- The LISI score should be calculated based on the Harmony integration that was performed.
Response to minor comment 2: The LISI scores for adipocytes (ciLISI), computed using the harmony embeddings and via scIntegrationMetrics v1.2.0 are 4.85 for MAFALDA 1, 5.35 for MAFALDA 2, and 5.32 for KOBS. Their median iLISI scores computed using the harmony embeddings and via lisi v1.0 are 4.07 for MAFALDA 1, 4.45 for MAFALDA 2, and 4.50 for KOBS. These median iLISI scores are comparable to iLISI scores of other previously published data (**Table B**). Please note that LISI scoring is sensitive to the number of cells/nuclei per batch [PMID: 31740819].

Table B. iLISI scores are comparable between previously published cohorts and MAFALDA 1, MAFALDA 2, and KOBS.

Cohort	Embedding	Integration by	Number of samples/batches	Median iLISI score
**Emont et al. adipocytes [PMID: 35296864]**	PCA	Sample	23	5.76
**Natri et al. [PMID: 38548990]**	PCA	Flowcell_ID	24	3.77
**Mareckova et al. [PMID: 39198675]**	scVI	Library_genotype	68	3.53
**MAFALDA 1 adipocytes**	Harmony	Sample_ID	11	4.07

**MAFALDA 2 adipocytes**	Harmony	Batch	8	4.45
**KOBS adipocytes**	Harmony	Batch	8	4.50

Table B footnote: iLISI indicates integration local inverse Simpson's Index; PCA, principal component analysis; and scVI single-cell variational inference.

- Figure 1c colors are not clear – if the purple is just meant to show adipocytes, then it is not a helpful figure.

Response to minor comment 3: We have now removed Figure 1c to address the Reviewer's comment.

- The colors in Suppl. Figure 1b, Suppl. Figure 3b-c, and Suppl. Figure 10b-c are too similar and it is not easy to distinguish each sample. A stacked BarPlot per sample should be used instead.

Response to minor comment 4: As suggested by the Reviewer, we have now replaced these figures with the stacked barplots.

- Point 3 of previous reviewer#2 has not been addressed properly. Indeed, there has been no experimental validation or coherent biological basis in this study, which is required to support the statements of this paper. Specifically, what does reduced latent time mean for these adipocytes? Are single-nuclei datasets appropriate for such kind of analysis that depends on splicing, or would single-cell data be more appropriate? Have the adipocytes stopped expanding and are unable to store more lipids and/or have they reached maximum size? In order to further test this hypothesis using the cross sectional data in the current study, additional information would be needed. Based on transcriptomic markers marking the adipocytes with "reduced latent time", can the authors identify and validate (e.g. via IHC) adipocytes with reduced latent time and make statements regarding their cell size and/or morphology?

Response to minor comment 5: We thank the Reviewer for this insightful comment. We have interpreted reduced latent time to mean that these adipocytes are incapacitated in their ability to store lipids, essentially not reaching their full adipogenic potential. This is based upon the finding that the adipocytes from individuals with MASLD appear to have a lower predicted degree of adipocyte differentiation than the adipocytes from individuals without MASLD. Unfortunately, we are unable to perform IHC due to lack of tissue samples available. However, to validate this, we have now leveraged VAT bulk RNA-seq from another Finnish bariatric surgery cohort, Roux-en-Y versus one-anastomosis gastric bypass (RYSA) (n=66) [PMID:

37987183] that unlike MAFALDA <u>also has adipocyte size/morphology data available</u>. The adipocyte size in RYSA was measured as described in detail before [PMID: 39515300].

To investigate whether reduced adipocyte latent time correlates with adipocyte size, we used the adipocyte size measurements and VAT bulk RNA-seq data in RYSA and performed a gene set enrichment analysis using fgsea [preprint: https://www.biorxiv.org/content/10.1101/060012v3]. In more detail, we first used limma-voom [PMID: 25605792] to identify genes, the VAT expression of which is associated with the adipocyte diameter in RYSA, adjusting for VAT cell-types proportions, age, sex, BMI, and technical factors. We used the following VAT bulk expressed genes among the predicted degree of VAT adipocyte differentiation gene set for the pathway parameters: the 263 genes that are DE by MASLD or MASH; the 145 genes that are upregulated by MASLD or MASH; and the 118 genes that are downregulated by MASLD or MASH (**Supplementary Table 27**). Following this, we found that there is a negative normalized enrichment score (NES) (NES=-1.77, p-value=9.17×10^{-7}) between the gene list that was ranked based on the correlation of the VAT expression with adipocyte diameter in RYSA and the 263 genes DE by MASLD or MASH in VAT adipocytes in MAFALDA. Furthermore, we found that this negative NES is mostly reflecting the 145 DE genes upregulated by MASLD or MASH (NES=-2.19, p-value=3.27×10^{-10}). This shows that there is a negative association between the expression of the VAT MASLD DE genes predicting the differentiation degree and adipocyte diameter in the RYSA cohort.

To further investigate this finding, we then built the first principal component (PC1) of the VAT bulk expression of the genes upregulated by MASLD or MASH and contributing most to the enrichment (n=48 genes) (**Supplementary Table 28**) (for selection of these genes, see **Supplementary Note 1.5**). Next, we tested this PC1 for association with adipocyte size in the RYSA cohort. We identified a negative trend (rho=-0.242, p-value=0.057) between PC1 of the VAT bulk expression (n=48 genes) and the adipocyte diameter in RYSA. Given that only bulk VAT RNA-seq data are available in RYSA, we then focused more on adipocyte expression by testing the adipocyte marker genes among the 48 genes (n=13 genes, **Supplementary Table 28**) and found that there is also a significant negative correlation (rho=-0.263, p-value=0.039) between PC1 of their VAT bulk expression and the adipocyte diameter in RYSA. Finally, when we investigated the marker genes of VAT adipocyte subtype hAd1 among the 48 genes (n=14 genes, **Supplementary Table 28**), we observed that there is a significant negative correlation (rho=-0.31, p-value=0.013) between PC1 of their VAT bulk expression and the adipocyte diameter in RYSA. This coincides with our findings that the latent time in hAd1 is lower (FDR<0.05) in the individuals with MASLD livers vs non-steatotic livers (**Figure 2h**, **Supplementary Table 21**) and that the marker genes of the hAd1 adipocyte subtype are enriched for preadipocyte specific pathways (**Figure 2k**, **Supplementary Table 25**), suggesting them to be earlier in their differentiation. Overall, these new adipocyte diameter results suggest

that the MASLD DE genes predicting the differentiation degree are also linked to smaller adipocyte size.
We have now added this information to the revised Abstract (page 3, lines 67-68), Methods (page 8, lines 154-161; page 17, lines 353-367; page 18, lines 368-381), Results (page 28, lines 586-588; page 34, lines 740-745; page 35, lines 746-768; page 36, lines 769-777) and Discussion (page 47, lines 1012-1015). We also include **Supplementary Tables 27** and **28** showing the genes in the gene sets tested.

Regarding the use of scVelo [PMID: 32747759] for single nucleus data, we would like to clarify that the transition from unspliced to spliced transcripts is indicative of the transformation from pre-mRNA to mature mRNA, which is applicable within single nucleus RNA-sequencing data. ScVelo attempts to ascertain gene-wise transcriptional dynamics and splicing kinetics, meaning that it takes into account the rate of transcription, splicing, and degradation. In single nucleus RNA-sequencing data, however, "degradation" might be better referred to as "export out of the nucleus". As splicing also occurs within the nucleus, the quantity of unspliced mRNA to mature, spliced mRNA levels can still be leveraged to determine whether a gene is being actively transcribed or repressed and predict the degree of differentiation for the overall cell (based upon the nuclear transcripts). We also acknowledge that to fully confirm this, a comparison between single nucleus and single cell RNA-sequencing of adipose tissue data would be needed. However, performing single cell RNA-seq in frozen human VAT samples is not technically possible as we cannot extract intact cells from fat-enriched frozen tissue. Furthermore, previous papers with single nucleus data have also utilized this same tool to better understand RNA splicing dynamics and uncover gene expression differences underlying disease-specific dysregulation, explore developmental progression of subtypes, and assess differentiation potential under specific conditions across various tissue types [PMID: 38538816, PMID: 36539501, PMID: 38934400, PMID: 40945691].

- The authors should further expand on the potential functional meaning of the increased latent time for hepatocytes in individuals with MASLD (lines 519-520)? What were the gene expression patterns characteristic of the hepatocytes with increased latent time? I may have missed it but did not see this.

Response to minor comment 6: We thank the Reviewer for giving us the opportunity to further explore these results. To address this comment and better characterize the hepatocytes with increased latent time, we overlapped the genes used to estimate hepatocyte latent time, i.e., the predicted degree of hepatocyte differentiation gene set (n=2,000), with the genes (n=4,122 genes) that were DE by MASLD in hepatocytes (**Supplementary Table 18**). Among those, 1,040 genes were DE by MASLD and contributed to the predicted degree of hepatocyte differentiation rate. We found that the genes that were upregulated by MASLD and involved in predicting the differentiation degree for hepatocytes (i.e., hepatocyte latent time gene set) (n=417

genes) were enriched in cholesterol and sterol esterification pathways (**Supplementary Figure 8, Supplementary Table 19**). This also matches with the characterization of zone 3 hepatocytes that we predicted to have an increased latent time. Both cholesterol esterification and lipogenesis are linked to tissue remodeling in the liver as a response to the influx of lipids in individuals with MASLD. This is in contrast with the genes downregulated by MASLD from the hepatocyte latent time gene set (n=623 genes) that were enriched for other liver-specific processes such as regulation of copper and zinc (**Supplementary Figure 8, Supplementary Table 20**). Based upon this, the hepatocytes with a higher latent time corresponding to individuals with MASLD seem to be involved in hepatocyte remodeling to accommodate the increase in lipids.
We have now added these new analyses and results to **Supplementary Note 1.4** and **1.13** and Results (page 31, lines 666-676; page 32, lines 677-681).

- Since Latent Times of adipocytes between the two groups are different, a question arises whether new adipocytes can be recruited from adipogenic progenitors (ASPCs). Perhaps the authors could mention this as a possibility for future study.

Response to minor comment 7: We thank the Reviewer for this helpful suggestion. We find this as a valuable future study and have now added this point to the revised Discussion (page 51, lines 1115-1117).

- Page 7, line 167 – what does "final concentrations of nuclei" mean? Please provide # of nuclei loaded.

Response to minor comment 8: As suggested by the Reviewer, we have replaced "final concentrations of nuclei" with the number of nuclei targeted in **Supplementary Table 2.**

- Were UMI cutoffs the same for each sample?
Response to minor comment 9: We confirm that the same UMI cutoff of 500 was used for all samples. We have now revised the Methods (page 13, lines 259-260) to clearly state this.

- Please provide the number of nuclei removed at each step. It would be helpful to include a diagram of the analysis pipeline – this may also allow the authors to summarize the analysis and move some of the details to supplementary methods.

Response to minor comment 10: We have now included a new diagram (**Supplementary Figure 1**) detailing our quality control (QC) pipeline. We have also added a new **Supplementary Table 2** showing the number of nuclei following each step of the QC process.

- Please explain why platelet count was used as a "negative phenotypic control." (line 497). One might anticipate that platelet counts differed in MASLD or fibrosis.

Response to minor comment 11: We thank the Reviewer for this helpful comment. We would like to clarify that we had performed a logistic regression analysis and found that platelet count did not differ between the individuals with and without MASLD or MASH or between the individuals with and without fibrosis in our data. We show this output below for the Reviewer (**Table C**). Therefore, based on this, to ensure that the latent time results were specific to MASLD, we used the platelet count as a negative control as it would not have been impacted by the MASLD status. We have now included this in the revised Methods (page 21, lines 452-454), Results (page 30, lines 631-634) and added **Supplementary Table 11**.

Table C. Blood platelet count is not significantly associated with MASLD or fibrosis in the MAFALDA 2 cohort.

	**Beta**	**SE**	**Z**	**P**
**MASLD**	0.0020	0.0043	0.46	0.64
**MASH**	0.0021	0.0053	0.39	0.70
**Fibrosis**	-0.0049	0.0048	-1.0	0.31

Footnote to Table C. A logistic regression analysis was performed to assess the relationship between blood platelet level and MASLD, MASH or fibrotic status. Blood platelet level was the predictor for both analyses. SE indicates standard error; Z, z-value; and P, p-value using the Wald test.

- Volcano plot labels cover up the actual data - please revise.

Response to minor comment 12: As suggested by the Reviewer, we have now revised the volcano plot. We removed the gene labels and replaced them with triangles to highlight the eGenes.
